# U‐shaped association between sleep duration and biological aging: Evidence from the UK Biobank study

**DOI:** 10.1111/acel.14159

**Published:** 2024-03-31

**Authors:** Xuanyang Wang, Xuemin Yan, Mengdi Li, Licheng Cheng, Xiang Qi, Jia Zhang, Sijia Pan, Xiaoqing Xu, Wei Wei, Ying Li

**Affiliations:** ^1^ Department of Nutrition and Food Hygiene, School of Public Health, Key Laboratory of Precision Nutrition and Health, Ministry of Education Harbin Medical University Harbin Heilongjiang China; ^2^ Department of Endodontics, The First Hospital Harbin Medical University Harbin China; ^3^ Department of Pharmacology, College of Pharmacy, Key Laboratory of Cardiovascular Research, Ministry of Education Harbin Medical University Harbin China

**Keywords:** aging, cystatin C, gamma glutamyltransferase, predicted age, sleep duration, UK Biobank study

## Abstract

Previous research on sleep and aging largely has failed to illustrate the optimal dose–response curve of this relationship. We aimed to analyze the associations between sleep duration and measures of predicted age. In total, 241,713 participants from the UK Biobank were included. Habitual sleep duration was collected from the baseline questionnaire. Four indicators, homeostatic dysregulation (HD), phenoAge (PA), Klemera–Doubal method (KDM), and allostatic load (AL), were chosen to assess predicted age. Multivariate linear regression models were utilized. The association of sleep duration and predicted age followed a U‐shape (All *p* for nonlinear <0.05). Compared with individuals who sleep for 7 h/day, the multivariable‐adjusted beta of ≤5 and ≥9 h/day were 0.05 (95% CI 0.03, 0.07) and 0.03 (95% CI 0.02, 0.05) for HD, 0.08 (95% CI 0.01, 0.14) and 0.36 (95% CI 0.31, 0.41) for PA, and 0.21 (95% CI 0.12, 0.30) and 0.30 (95% CI 0.23, 0.37) for KDM. Significant independent and joint effects of sleep and cystatin C (CysC) and gamma glutamyltransferase (GGT) on predicted age metrics were future found. Similar results were observed when conducting stratification analyses. Short and long sleep duration were associated with accelerated predicted age metrics mediated by CysC and GGT.

## Graphical abstract

U‐shaped association between sleep duration and biological aging, especially in populations that are not considered elderly.

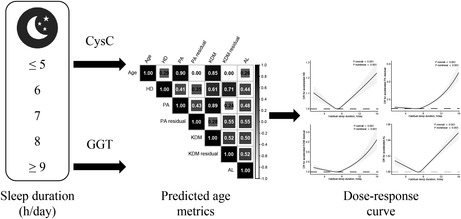

**Abbreviations**
AAs: age accelerations
AL: allostatic load
BMI: body mass index
CI: confidence interval
CVD: cardiovascular diseases
CysC: cystatin C
GGT: gamma glutamyltransferase
HD: homeostatic dysregulation
KDM: Klemera–Doubal method
NHANES: National Health and Nutrition Examination Survey
PA: phenoAge
PRS: polygenic risk score
RCS: restricted cubic spline
SNPs: single nucleotide polymorphisms
SWS: slow‐wave sleep
TDI: Townsend deprivation index
TL: telomere length

## 1 INTRODUCTION

Aging is a multifaceted process encompassing various biological changes over time, such as the gradual accumulation of molecular and cellular damage, which can result in a decline in function, an increased vulnerability to chronic diseases, and eventually mortality (Moqri et al., 2023). Throughout our lives, various factors including genetic predispositions, environmental exposures, and lifestyle choices contribute to this accumulation of damage (Gladyshev et al., 2021). This means that the strategies emphasizing various aspects of daily life are essential for aging risk management.

Currently, various quantitative parameters of aging have been developed to identify and evaluate interventions for human longevity (Galkin et al., 2020). Instead of comprehensively capturing the complexity of biological aging, different biomarkers provide unique windows or insights through unique lenses. Recent research has illustrated that clinical assessments of biological aging developed from multiple biomarkers and a control group can predict chronological age with reasonably good precision (Chaney & Wiley, 2023). Such predictions characterize the internal aging processes, reflecting an individual's health and functionality concerning their chronological age (Ferrucci et al., 2020), such as HD (Cohen et al., 2013), PA (Levine et al., 2018), KDM (Earls et al., 2019), and AL (Shirazi et al., 2020). However, many of these predictions simply forecast chronological age but fail to capture the outcomes once the standard confounding variables are adjusted (Bell et al., 2019; Bernabeu et al., 2023; Zhang et al., 2019). Aging measures from biomarkers to clocks serve as mere indications of predicted age rather than instruments that comprehensively capture biological aging (Moqri et al., 2024).

Sleep is a physiological state in which the body is resting and recovering, usually occurring at night. So far, the reciprocal causal association of sleep with accelerated aging is still a subject of debate. Alterations in sleep characteristics, including quality, quantity, and architecture, have been discovered to be associated with age‐related health outcomes and disorders (Fernandez‐Mendoza & Vgontzas, 2013; Gao et al., 2022). Meanwhile, sleep is also one of the biological processes most affected by aging, especially in the elderly. As individuals age, notable changes occur in sleep patterns and quality as well as the duration of sleep (Tempaku et al., 2015). Given the coexistence of irreversible aging process and age‐associated sleep modifications, an increased incidence of inadequate and extended duration of sleep and age‐related ailments as well as notable discrepancies in cellular senescence indicators, we believe it is important to use the optimal dose–response curve to accurately characterize the relationship between sleep duration and aging.

Notably, most of the published investigations with age‐related health outcomes as endpoints have focused on sleep quality (Koshmanova et al., 2023), behavior (Kianersi et al., 2023), patterns (Zhou et al., 2022), disorders (Overton et al., 2023), and environment (de Leeuw et al., 2023), and have not yet explored an association of sleep duration with biological aging. In the present study, we investigated the associations of habitual sleep duration with four predicted age metrics, including HD, PA, KDM, and AL among those enrolled in the UK Biobank study.

## 2 METHODS

### 2.1 Study population

The UK Biobank is a sizable, population‐oriented investigation encompassing over 500,000 individuals between the ages of 37 and 73 years residing within the United Kingdom (Sudlow et al., 2015). The methodology of this study has been previously outlined in other literature (Fry et al., 2017). The ethical clearance for the UK Biobank was granted by the North West Multi‐Center Research Ethics Committee (REC reference: 11/NW/0,3820). Before being enrolled in the study, all participants provided written consent after being fully informed, adhering to the principles outlined in the Declaration of Helsinki.

**FIGURE 1:**
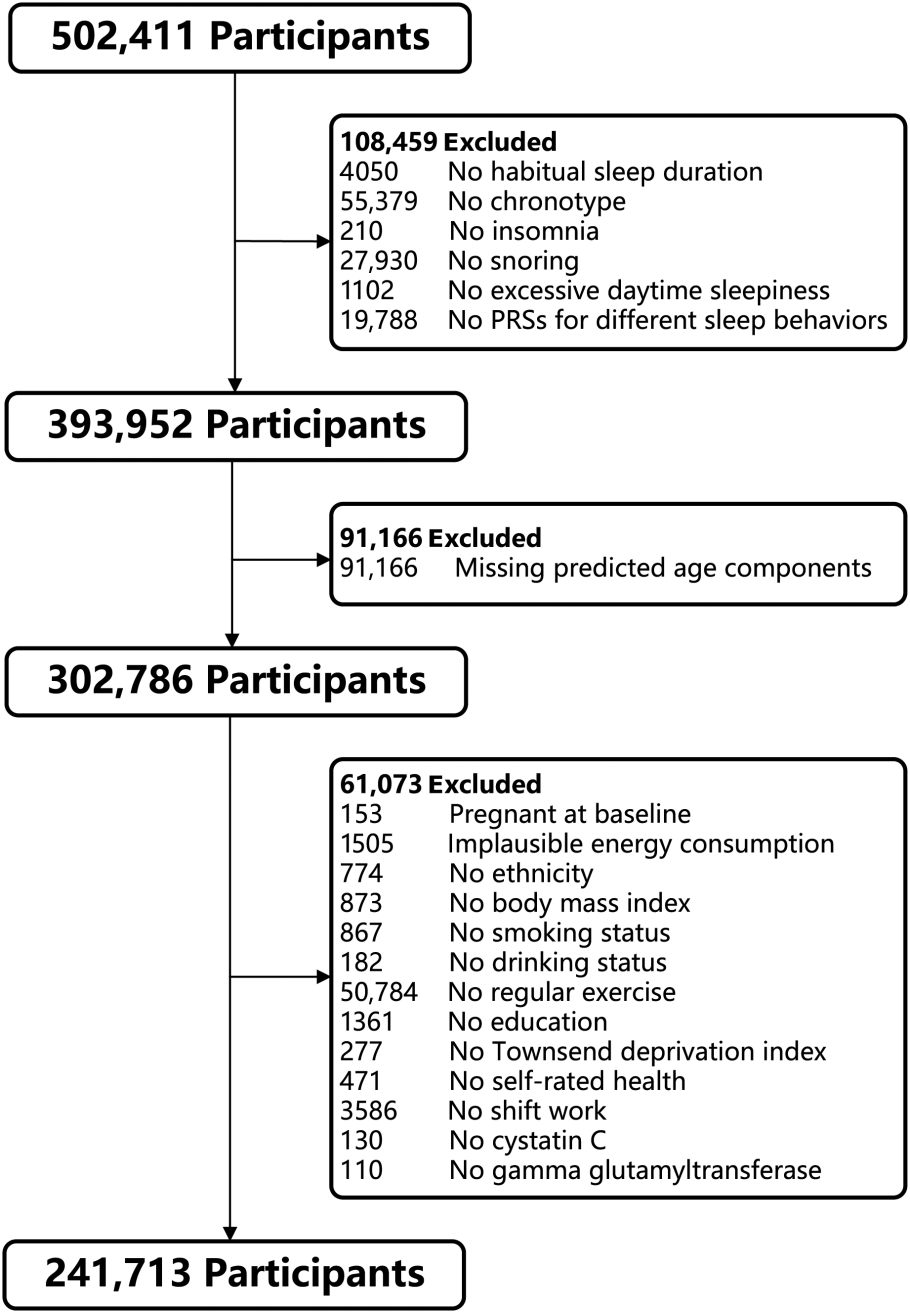
Flowchart illustrating the criteria for inclusion.

In the current study, the criteria for exclusion were as outlined below: (1) not available data on habitual sleep duration, sleep behaviors, and polygenic risk scores (PRSs) for different sleep behaviors; (2) missing data with molecular and physiological biomarkers necessary to develop predicted age metrics; (3) pregnant at baseline, implausible energy consumption (Chen, Cao, et al., 2023), and missing data with covariates. Following the exclusion of the previously mentioned participants, a cohort of 241,713 individuals was considered for further analysis in this cross‐sectional study (Figure 1).

### 2.2 Assessment of habitual sleep duration

The baseline questionnaire includes a self‐reported question regarding habitual sleep duration, which asked participants, “About how many hours sleep do you get in every 24 hours? (please include naps).” If the duration of sleep varies significantly, individuals provide the average sleep duration for a 24‐h day over the past 4 weeks. We categorized the participants into five groups (≤5, 6, 7, 8, ≥9 h/day) and considered the 7 h/day as the reference category (Han et al., 2023).

### 2.3 Ascertainment of predicted age metrics

By utilizing the most credible algorithms, HD, PA, KDM, and AL were developed based on 10 blood chemistry parameters (Table S1) (Kwon & Belsky, 2021). Following the protocols established by Nakazato et al. (2020), Levine et al. (2018), and Klemera and Doubal (2006), the blood‐chemistry‐based measures for HD, PA, and KDM were initially trained utilizing data obtained from the 1988–1994 National Health and Nutrition Examination Survey (NHANES III). The algorithms and R code utilized for the analysis were made accessible through the “BioAge” R package, which can be found at https://github.com/dayoonkwon/BioAge. The Mahalanobis distance metric was used in HD to assess the deviation of an individual from a healthy population (Shirazi et al., 2020). PA was constructed by analyzing various factors related to mortality risks by applying elastic‐net Gompertz regression, enabling the estimation of the likelihood of death (Levine, 2013). Quantitative assessment of system integrity decline measured by KDM was accomplished by performing a sequence of regression analyses between certain biomarkers and chronological age within the reference population (Kwon & Belsky, 2021). AL represented the cumulative effects of chronic stress and life experiences on an individual's physical health and its determination involved assessing the percentage of biomarkers indicating an elevated risk (McEwen & Stellar, 1993). In this study, the risk level was established by assessing individuals positioned within the top 25% of a particular biomarker's distribution for 9 out of the 10 biomarkers. Regarding albumin, earlier research findings deemed individuals in the lowest quartile to be at risk (Shirazi et al., 2020). The final AL, which varied from 0 to 1, was considered as the ratio of biomarkers classified as “at risk” among the 10 selected biomarkers (Duong et al., 2017).

The residual differences between the estimated predicted age metrics and chronological age were identified as age accelerations (AAs), as this approach sought to mitigate inconsistencies among the measurement platforms of each constituent of predicted age metrics (Hägg et al., 2019; Horvath & Raj, 2018). To determine the predicted age and chronological age, residuals were computed using a linear regression analysis with either PA or KDM as the response variable and chronological age as the predictor (Mak et al., 2023). No residuals were computed for HD and AL, given that they were not age metrics by definition and already accounted for deviation from a reference population (Graf et al., 2022; Kwon & Belsky, 2021). Higher HD, PA residuals, KDM residuals, and AL values signify accelerated aging (Chaney & Wiley, 2023; Obeng‐Gyasi et al., 2023; Ye et al., 2023). Subsequently, the focus in subsequent analyses was on HD, PA residuals, KDM residuals, and AL as the primary outcomes.

### 2.4 Assessment of mediation variables

The UK Biobank measured the biochemical markers in samples from roughly 480,000 participants at baseline, with about 18,000 samples collected during subsequent evaluations. Serum CysC was examined using a latex‐enhanced immunoturbidimetric assay conducted by Siemens (Erlangen, Germany) on the Siemens Advia 1800, with a 1.1% interassay coefficient of variation (Chen, Lees, et al., 2023). Serum GGT concentration was assessed using an enzymatic rate technique on a Beckman Coulter AU5800 analyzer, with a detection limit spanning from 5 to 1200 U/L (Liao et al., 2023). Sample quality control was performed using third‐party Internal Quality Control material provided by Randox Laboratories and Technopath, and externally verified through WEQAS Mainline Chemistry. Additional details regarding assay performance have been documented on the UK Biobank website (www.biobank.ac.uk/).

### 2.5 Definition of the PRSs

Comprehensive explanations of the genotyping, imputation, and quality assessment procedures conducted by the UK Biobank can be found elsewhere (Bycroft et al., 2018). The single nucleotide polymorphisms (SNPs) selected to calculate PRSs for chronotype (351) (Jones et al., 2019), habitual sleep duration (112) (Doherty et al., 2018), insomnia (248) (Gao et al., 2020), daytime sleepiness (123) (Dashti et al., 2021), snoring (41) (Campos et al., 2020), KDM (16) (Gao et al., 2022), and PA (29) (Kuo et al., 2021) were well‐documented in research pertaining to the genetic variants associated with the respective phenotypes (Tables S2–S8). We utilized a weighted approach, employing the subsequent formula, calculated using PLINK and R‐project, while also considering 10 genetic principal components for adjustment:

$${\mathrm{PRS}}_{j}=\frac{\sum_{i}^{N}S_{i}\times G_{\mathrm{ij}}}{P\times M_{j}}.$$

PLINK was employed to retrieve details regarding the magnitude of effect for each SNP, the count of observed effect alleles, and the overall count of SNPs encompassed within the PRS. Following that, the R‐project was leveraged to execute the effective calculation of the PRS utilizing a weighted approach. The magnitude of SNP_*i*_ is denoted as *S*_*i*_; the count of observed effect alleles in sample_*j*_ is *G*_*ij*_; the sample's ploidy is represented by *P* (typically 2 for humans); the overall count of SNPs included is *N*; the count of non‐missing SNPs observed in sample_*j*_ is *M*_*j*_. Eventually, the PRS varies between 0 and 0.1 (Choi et al., 2020). The variances explained by the resulting PRSs in the odds of different sleep behaviors and predicted age metrics were showed in Table S9.

### 2.6 Assessment of covariates

The covariates were chosen from a set of traditional variables or possible factors that may impact sleep or biological aging, including age (years), sex (male/female), ethnicity (White/Mixed/Asian or Asian British/Black or Black British/Chinese/Another ethnic group), body mass index (BMI) (kg/m^2^), smoking status (never/previous/current), drinking status (never/previous/current), physical activity (low/moderate/high), education level (below high school/high school/above high school), Townsend deprivation index (TDI), diet quality score, overall health rating (excellent/good/fair/poor), self‐reported type 2 diabetes, hypertension, cardiovascular disease (CVD) and cancer (yes/no), medication for cholesterol, blood pressure or diabetes (yes/no), family history of type 2 diabetes, hypertension, CVD and cancer (yes/no), sleep disorder (yes/no), depression (yes/no), and shift work (yes/no). For comprehensive variable definitions, see Table S10 and the UK Biobank website (www.biobank.ac.uk/).

### 2.7 Statistical analysis

Baseline characteristics were presented as means (95% CI) or *N* (%) for continuous variables and categorical variables within the various categories of habitual sleep duration. Multivariate linear regression models were performed using the lm function and controlling for the main effects of the variables adjusted to examine the associations between habitual sleep duration and CysC and GGT and the associations of habitual sleep duration with predicted age metrics. The linear trend of habitual sleep duration was evaluated by replacing it with the median of the amount and treating the variable as continuous in the analysis. The shaping of the dose–response curves, depicting the associations between habitual sleep duration and predicted age metrics, was accomplished using restricted cubic spline (RCS) regression with three knots (10%, 50%, and 90% of habitual sleep duration) and rms package (version 6.7.1). The reference values were the habitual sleep duration at odd ratio estimates of 1.0 (Johannesen et al., 2020). The evaluation of the mediation effects of CysC and GGT in the associations of habitual sleep duration with predicted age metrics was conducted through mediation analysis using mediation package (version 4.5.0).

To validate the reliability of our findings, multiple sensitivity analyses were conducted. First, we excluded individuals engaged in shift work to minimize any potential misclassification of the effects of work during different periods. Second, we excluded cases diagnosed with depression at baseline to circumvent the potential impact of depression on sleep duration. Third, we excluded participants with sleep disorders to approximate sleep duration to that of the general population. Fourth, we eliminated subjects who rated their health as poor to investigate the possible effects of subpar health conditions on sleep duration. Fifth, given the significance of other sleep characteristics in sleep studies, we further performed previous analyses on individuals with additional sleep characteristics, including excessive daytime sleepiness (yes/no), snoring (yes/no), insomnia (yes/no), chronotype (morning person/evening person), and sleep patterns (healthy/poor). Sleep characteristics, as determined by a particular question for each trait, were documented via self‐reporting using a touchscreen questionnaire Table S11 (Fan et al., 2020). Each sleep factor was categorized as a binary variable, with “1” indicating low risk and “0” indicating high risk. Finally, we were able to calculate a sleep score on a scale of 0–5. A poor sleep pattern was defined as a sleep score ≤2, and a healthy sleep pattern was indicated by a score of 3–5. Sixth, we conducted stratified analyses on individuals grouped by median of PRS (chronotype/habitual sleep duration/insomnia/daytime sleepiness/snoring/KDM/PA) to assess whether genetic predisposition to sleep characteristics or indicators of aging can influence the associations of habitual sleep duration with predicted age metrics. Seventh, we conducted multiple stratified analyses to assess potential modifying impacts of the following factors: age (>60 or ≤60), sex (male or female), BMI (>30 or ≤30), smoking status (yes or no), drinking status (yes or no), physical activity (high or moderate or low), education level (above high school or high school and below), and TDI (≤−3.27 or −3.26 to −0.99 or >−0.98).

All statistical analyses were performed using R 4.1.1, with a *p*‐value based on Bonferroni correction considered statistically significant when *p* < 0.01 (0.05/5 outcomes = 0.01).

## 3 RESULTS

### 3.1 Baseline characteristics

The characteristics of the subjects based on their sleep duration are documented in Table 1. Out of the 241,713 individuals, 4.4%, 17.9%, 39.5%, 30.5%, and 7.8% indicated a sleep duration of ≤5, 6, 7, 8, and ≥9 h/day, respectively. In comparison with individuals who had a habitual sleep duration of 7 h/day, those with a shorter or longer duration of sleep were more likely to be older, female, smokers, non–drinkers, poor health, sleep disorders, depression, and excessive daytime sleepiness; have higher BMI, socioeconomic status, use of medication for cholesterol, blood pressure or diabetes, prevalence and family history of chronic diseases, CysC, and GGT; as well as were of lower education.

**TABLE 1:** Baseline characteristics of participants by sleep duration (*n* = 241,713)^a^.

	Habitual sleep duration, h/day					*p*	*p*_test_
	≤5 (*n* = 10,641)	6 (*n* = 43,154)	7 (*n* = 95,469)	8 (*n* = 73,640)	≥9 (*n* = 18,809)		
Age, years	56.89 (56.74, 57.03)	56.17 (56.09, 56.24)	55.64 (55.59, 55.69)	56.86 (56.80, 56.91)	58.56 (58.45, 58.67)	<0.001	<0.001
Male, *n* (%)	4871 (45.8)	21,449 (49.7)	47,060 (49.3)	33,672 (45.7)	8691 (46.2)	<0.001	<0.001
White, *n* (%)	10,274 (96.6)	41,949 (97.2)	93,438 (97.9)	72,124 (97.9)	18,383 (97.7)	<0.001	0.010
BMI, kg/m^2^	28.39 (28.29, 28.49)	27.68 (27.64, 27.73)	27.00 (26.97, 27.03)	27.05 (27.02, 27.08)	27.86 (27.79, 27.93)	<0.001	<0.001
Current smoker, *n* (%)	1571 (14.8)	5058 (11.7)	8943 (9.4)	6701 (9.1)	2104 (11.2)	<0.001	<0.001
Current drinker, *n* (%)	9396 (88.3)	40,077 (92.9)	90,211 (94.5)	69,002 (93.7)	17,090 (90.9)	<0.001	<0.001
Regular exercise, *n* (%)	4398 (41.3)	17,793 (41.2)	38,525 (40.4)	30,267 (41.1)	7164 (38.1)	<0.001	0.013
Above high school, *n* (%)	5414 (50.9)	26,169 (60.6)	63,712 (66.7)	46,222 (62.8)	10,298 (54.8)	<0.001	<0.001
Townsend deprivation index	−0.73 (−0.79, −0.67)	−1.34 (−1.37, −1.31)	−1.67 (−1.69, −1.66)	−1.73 (−1.76, −1.71)	−1.41 (−1.45, −1.36)	<0.001	0.021
Diet quality	1.95 (1.93, 1.96)	1.94 (1.93, 1.94)	1.95 (1.94, 1.95)	1.97 (1.97, 1.98)	1.94 (1.93, 1.95)	<0.001	<0.001
Poor self‐rated health, *n* (%)	1305 (12.3)	1975 (4.6)	2045 (2.1)	1939 (2.6)	1591 (8.5)	<0.001	<0.001
Self‐reported diabetes, *n* (%)	1323 (12.4)	3552 (8.2)	6040 (6.3)	5305 (7.2)	2297 (12.2)	<0.001	<0.001
Self‐reported hypertension, *n* (%)	5076 (47.7)	16,885 (39.1)	32,778 (34.3)	26,952 (36.6)	8638 (45.9)	<0.001	<0.001
Self‐reported cardiovascular diseases, *n* (%)	6051 (56.9)	20,435 (47.4)	40,152 (42.1)	33,216 (45.1)	10,317 (54.9)	<0.001	<0.001
Self‐reported cancer, *n* (%)	2412 (22.7)	9476 (22.0)	20,399 (21.4)	17,254 (23.4)	5030 (26.7)	<0.001	<0.001
Family history of diabetes, *n* (%)	2530 (23.8)	9621 (22.3)	20,235 (21.2)	15,663 (21.3)	4251 (22.6)	<0.001	0.016
Family history of hypertension, *n* (%)	5337 (50.2)	21,682 (50.2)	47,929 (50.2)	35,743 (48.5)	9009 (47.9)	<0.001	<0.001
Family history of cardiovascular diseases, *n* (%)	5082 (47.8)	19,596 (45.4)	42,279 (44.3)	33,084 (44.9)	8785 (46.7)	<0.001	<0.001
Family history of cancer, *n* (%)	4083 (38.4)	15,850 (36.7)	34,129 (35.7)	27,115 (36.8)	7103 (37.8)	<0.001	<0.001
Sleep disorders, *n* (%)	832 (7.8)	1676 (3.9)	2331 (2.4)	1800 (2.4)	853 (4.5)	<0.001	<0.001
Depression, *n* (%)	1841 (17.3)	3769 (8.7)	5431 (5.7)	4788 (6.5)	2425 (12.9)	<0.001	<0.001
Shift work, *n* (%)	2021 (19.0)	9213 (21.3)	19,710 (20.6)	13,468 (18.3)	2720 (14.5)	<0.001	<0.001
Cystatin C, mg/L	0.94 (0.93, 0.94)	0.91 (0.91, 0.91)	0.89 (0.89, 0.89)	0.90 (0.90, 0.90)	0.94 (0.94, 0.94)	<0.001	<0.001
Gamma glutamyltransferase, U/L	43.12 (42.09, 44.15)	38.41 (38.02, 38.81)	35.62 (35.39, 35.85)	36.34 (36.06, 36.63)	41.40 (40.66, 42.14)	<0.001	<0.001
Homeostatic dysregulation	2.77 (2.75, 2.79)	2.64 (2.63, 2.64)	2.58 (2.57, 2.58)	2.64 (2.64, 2.65)	2.78 (2.77, 2.80)	<0.001	<0.001
PhenoAge	53.04 (52.87, 53.20)	51.84 (51.76, 51.93)	51.00 (50.94, 51.06)	52.31 (52.24, 52.38)	54.77 (54.63, 54.91)	<0.001	<0.001
PhenoAge residual	0.59 (0.50, 0.67)	0.15 (0.11, 0.19)	−0.15 (−0.17, −0.12)	−0.11 (−0.14, −0.08)	0.58 (0.51, 0.64)	<0.001	<0.001
Klemera–Doubal method	55.93 (55.76, 56.10)	54.66 (54.58, 54.74)	53.87 (53.81, 53.93)	55.28 (55.22, 55.35)	57.46 (57.32, 57.59)	<0.001	<0.001
Klemera–Doubal method residual	0.66 (0.56, 0.75)	0.09 (0.04, 0.13)	−0.18 (−0.21, −0.15)	0.04 (0.00, 0.07)	0.54 (0.46, 0.61)	<0.001	<0.001
Allostatic load	0.27 (0.27, 0.27)	0.25 (0.25, 0.25)	0.23 (0.23, 0.23)	0.24 (0.24, 0.24)	0.27 (0.27, 0.27)	<0.001	<0.001
Morning person, *n* (%)	6942 (65.2)	27,718 (64.2)	59,819 (62.7)	46,840 (63.6)	10,908 (58.0)	<0.001	0.002
No insomnia, *n* (%)	883 (8.3)	7179 (16.6)	24,302 (25.5)	22,983 (31.2)	5709 (30.4)	<0.001	<0.001
No excessive daytime sleepiness, *n* (%)	10,081 (94.7)	41,781 (96.8)	93,608 (98.1)	72,239 (98.1)	17,865 (95.0)	<0.001	<0.001
No snoring, *n* (%)	3578 (33.6)	16,132 (37.4)	35,368 (37.0)	27,652 (37.6)	7632 (40.6)	<0.001	<0.001
Medication for cholesterol, blood pressure or diabetes, *n* (%)	1777 (16.7)	5439 (12.6)	10,113 (10.6)	8955 (12.2)	2967 (15.8)	<0.001	<0.001

^a^ Continuous variables were presented as mean (95% CI). Categorical variables were listed as *N* (%), and *p*_test_ was the result of Bonferroni correction.

As for the various quantitative parameters of aging selected in this study, participants' predicted age metrics and their chronological ages were highly correlated (Figure S1). Those participants were more prone to have higher HD, PA residuals, KDM residuals, and AL.

### 3.2 Associations of sleep duration with predicted age metrics

**FIGURE 2:**
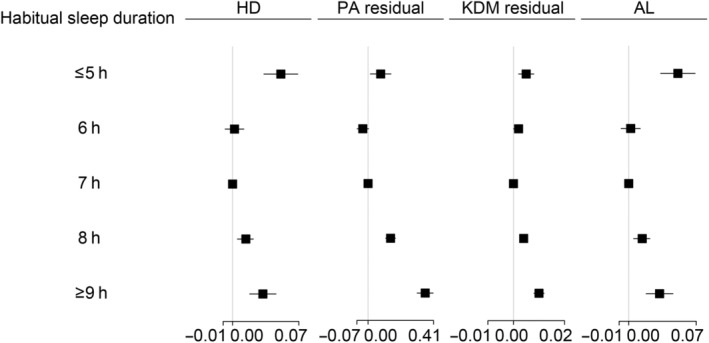
Associations between sleep duration and predicted age metrics. Data were beta estimates and 95% confidence intervals (CI) calculated using multivariate linear regression models. Results were adjusted for age, sex, ethnicity, BMI, smoking, drinking, regular exercise, education, Townsend deprivation index, diet score, overall health rating, self‐reported diabetes, hypertension, cardiovascular diseases (CVD), cancer, medication for cholesterol, blood pressure or diabetes, family history of diabetes, hypertension, CVD, cancer, sleep disorders, depression, and shift work. Those who slept for 7 h/day were the reference.

**FIGURE 3:**
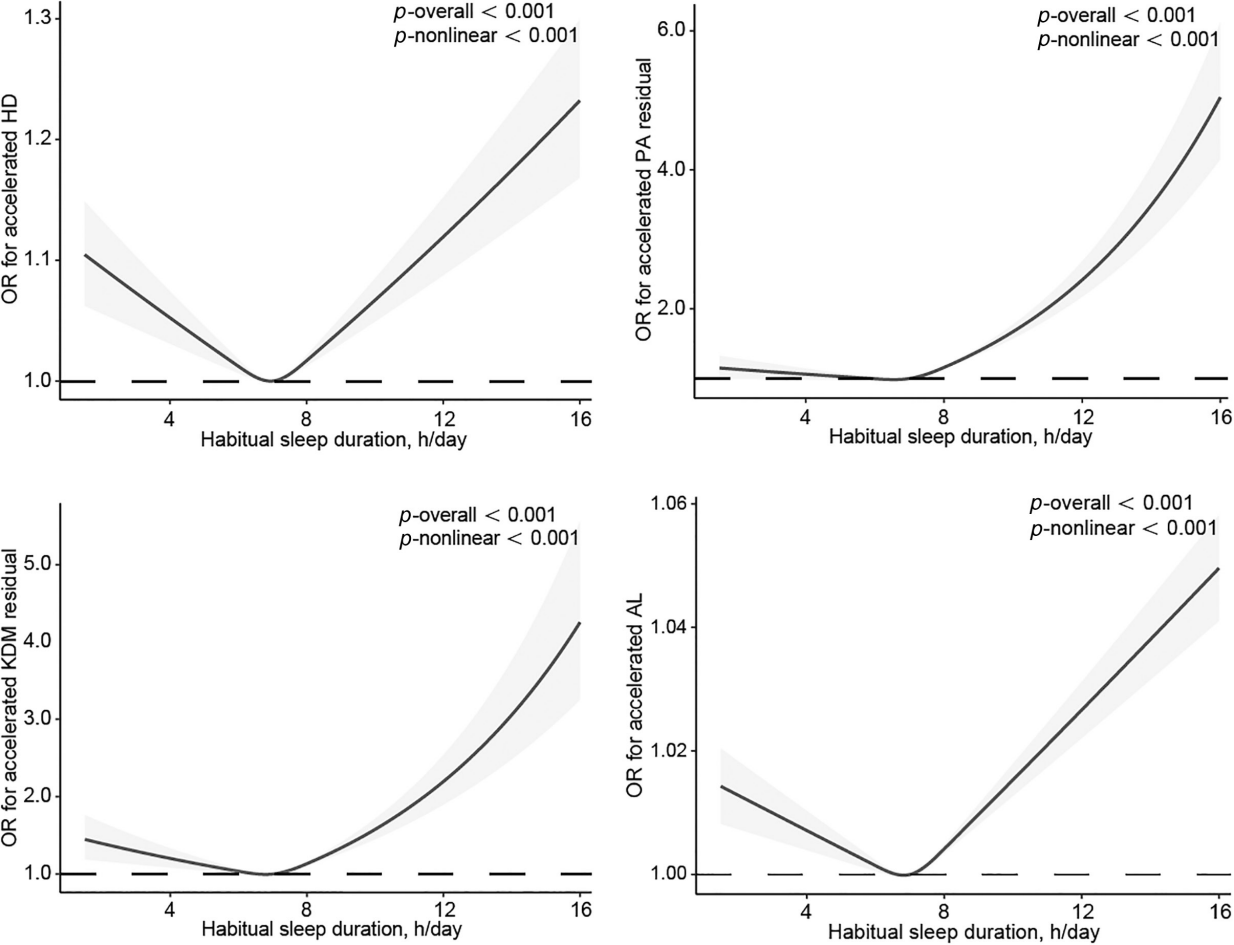
Smoothing curve for the associations of sleep duration expressed as h/day with predicted age metrics. Results were adjusted for age, sex, ethnicity, BMI, smoking, drinking, regular exercise, education, Townsend deprivation index, diet score, overall health rating, self‐reported diabetes, hypertension, CVD, cancer, medication for cholesterol, blood pressure or diabetes, family history of diabetes, hypertension, CVD, cancer, sleep disorders, depression, and shift work. *p* for overall was calculated using the joint Wald test for both the linear and nonlinear terms of RCS, whereas the *p* for nonlinear was obtained through the Wald test specifically for the nonlinear term of RCS.

Straying from the habitual sleep duration of 6–7 h/day was independently associated with an elevated risk of accelerated HD, PA, and KDM (Figure 2, Table S12). Upon conducting additional adjustments for sleep disorders, depression, and shift work (model 4), the associations were marginally attenuated. Compared with sleeping for 7 h/day, the multivariable‐adjusted beta estimates of accelerated HD were 0.05 (0.03, 0.07) for ≤5 h/day, 0.01 (0.01, 0.02) for 8 h/day, and 0.03 (0.02, 0.05) for ≥9 h/day, the beta estimates of accelerated PA were 0.08 (0.01, 0.14) for ≤5 h/day, 0.14 (0.11, 0.17) for 8 h/day, and 0.36 (0.31, 0.41) for ≥9 h/day, and beta estimates of higher KDM were 0.21 (0.12, 0.30) for ≤5 h/day, 0.15 (0.10, 0.19) for 8 h/day, and 0.30 (0.23, 0.37) for ≥9 h/day. For AL, a habit sleep duration of ≥9 h/day was associated with a 1.0% higher risk for higher AL 0.01 (0.01, 0.01). Figure 3 illustrated the results of RCS regression utilized to accurately represent the associations between habitual sleep duration and predicted age metrics (Figure 3). U‐shaped patterns were observed in the associations of sleep duration with higher HD (*p*_overall_ < 0.001, *p*_nonlinearity_ < 0.001), KDM (*p*_overall_ < 0.001, *p*_nonlinearity_ < 0.001), and AL (*p*_overall_ < 0.001, *p*_nonlinearity_ < 0.001). Moreover, the association of sleep duration with accelerated PA (*p*_overall_ < 0.001, *p*_nonlinearity_ < 0.001) displayed U‐shaped patterns, although statistical significance was not achieved on one side of this smooth curve.

### 3.3 Joint effects of predicted age accelerations and sleep behaviors or patterns

**FIGURE 4:**
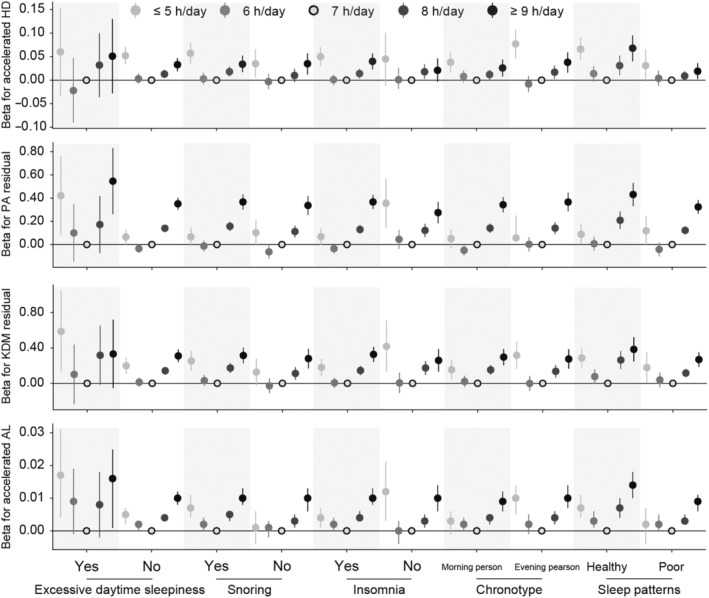
Associations between sleep duration and predicted age metrics among participants with different sleep behaviors and patterns at baseline. Data were beta estimates and 95% CI calculated using multivariate linear regression models. Results were adjusted for age, sex, ethnicity, BMI, smoking, drinking, regular exercise, education, Townsend deprivation index, diet score, overall health rating, self‐reported diabetes, hypertension, CVD, cancer, medication for cholesterol, blood pressure or diabetes, family history of diabetes, hypertension, CVD, cancer, sleep disorders, depression, and shift work. Those who slept for 7 h/day were the reference.

After stratifying the analysis based on other sleep characteristics, we observed similar associations between habitual sleep duration and predicted age metrics in each subgroup (*p*_interaction_ > 0.05) (Figure 4). Long sleepers with healthy sleep pattern had higher PA 0.43 (0.33, 0.53), KDM 0.38 (0.25, 0.52), and AL 0.01 (0.01, 0.02) (*p*_interaction_ < 0.05). No significant association of additional sleep characteristics with predicted age metrics was detected among participants with varying sleep durations.

### 3.4 Joint effects of predicted age accelerations and genetic susceptibility

**FIGURE 5:**
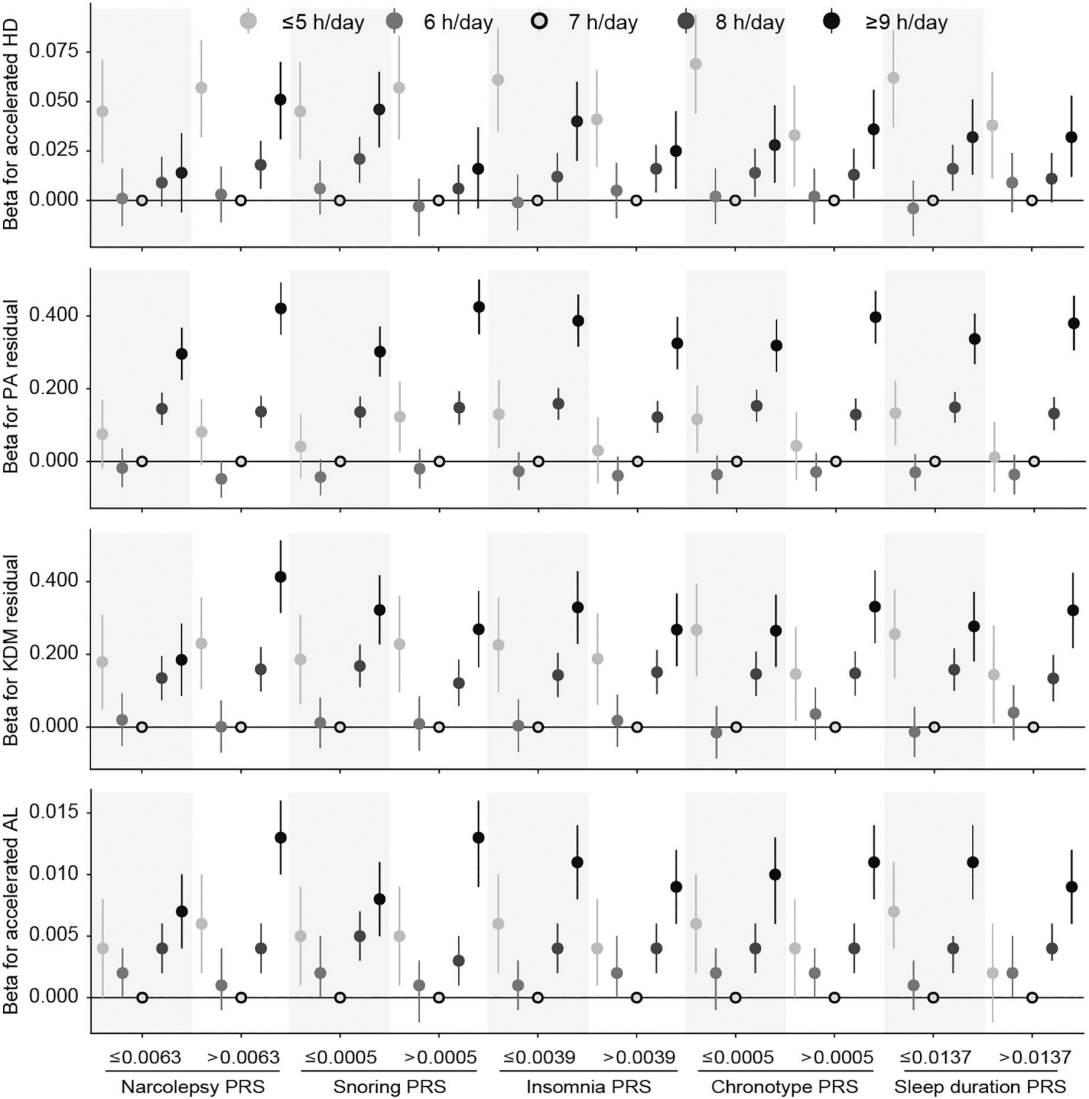
Associations between sleep duration and predicted age metrics among participants with PRSs for different sleep behaviors at baseline. Data were beta estimates and 95% CI calculated using multivariate linear regression models. Results were adjusted for age, sex, ethnicity, BMI, smoking, drinking, regular exercise, education, Townsend deprivation index, diet score, overall health rating, self‐reported diabetes, hypertension, CVD, cancer, medication for cholesterol, blood pressure or diabetes, family history of diabetes, hypertension, CVD, cancer, sleep disorders, depression, and shift work. Those who slept for 7 h/day were the reference.

When the analysis was segmented based on the background genetic risk for five sleep characteristics (chronotype/sleep duration/insomnia/daytime sleepiness/snoring), consistent associations were observed (*p*_interaction_ > 0.05) (Figure 5). No significant association of genetic susceptibility for PA/KDM with predicted age metrics was found among participants with different sleep durations (*p*_interaction_ > 0.05) (Figure S2).

### 3.5 Effects mediated by CysC and GGT on the associations between habitual sleep duration and predicted age metrics

The associations of sleep duration with CysC and GGT displayed U‐shaped patterns (*p*‐nonlinear < 0.001) (Figure S3). Accordingly, when grouped by sleep duration, the multivariable‐adjusted beta estimates of CysC were 0.01 (0.01, 0.01) for ≤5 h/day and 0.02 (0.01, 0.02) for ≥9 h/day, and the beta estimates of GGT were 3.57 (2.77, 4.37) for ≤5 h/day and 3.08 (2.46, 3.70) for ≥9 h/day (Table S13). Mediation analyses were conducted to examine whether there were mediation effects of CysC and GGT on the associations (Figure S4). The total effects of habitual sleep duration on HD (*β*_Tot_ = 0.021, *p* < 0.001), PA (*β*_Tot_ = 0.055, *p* < 0.001), KDM (*β*_Tot_ = 0.009, *p* < 0.001), and AL (*β*_Tot_ = 0.015, *p* < 0.001) were estimated using standardized regression coefficients. Furthermore, the mediated indirect effects attributed to CysC and GGT accounted for a distinct proportion of the total effects on HD (10.5, 3.9%), PA (12.6, 0.6%), KDM (37.6, 7.0%), and AL (35.9, 5.0%).

### 3.6 Other sensitivity analyses

The findings were not substantially changed when the analysis was limited to participants who were not employed or employed without night shifts, depression, sleep disorders, or self‐report poor health at baseline (Tables S14–S17). Individuals with longer sleep duration and lower BMI (≤30) appeared to exhibit higher levels of HD and KDM (*p*_interaction_ < 0.05). The associations of sleep duration with accelerated PA and KDM were particularly evident among participants with higher TDI (>−0.98) (*p*_interaction_ < 0.05). Short and long sleepers aged 60 or below showed accelerated PA, KDM, and increased HD, AL compared to other subgroups (*p*_interaction_ < 0.05). Similar findings were found in the analyses analyzed separately based on sex, ethnicity, smoking, drinking, exercise, and education (Tables S18–S21).

## 4 DISCUSSION

In this relatively large‐scale, nationwide, cross‐sectional study among general Britons, there were U‐shaped associations of sleep durations with predicted age metrics. Sleep duration of 5 h or less had at least a 5% higher risk of larger HD, and accelerated KDM, PA. Conversely, individuals who slept for 9 h or more had a minimum of 1% increased risk of accelerated HD, KDM, PA, and AL. Both insufficient and excessive sleep durations were associated with poorer self‐reported health and various lifestyle outcomes, implying U‐shaped associations of sleep duration with these factors. The same findings were also found after controlling for confounding factors and almost independent of other sleep characteristics and relevant genetic susceptibility. The detrimental impacts of insufficient or excessive sleep duration on biological aging were partially mitigated by CysC and GGT, emphasizing the crucial role of preserving appropriate sleep.

The consistent findings aligned with previous research conducted among elderly individuals in China, which suggested a greater incidence of successful aging among participants who slept approximately 7 h/day, while shorter (<6 h/day) and longer (≥9 h/day) sleep durations were associated with reduced prevalences of successful aging (Liu et al., 2016). A study conducted among Koreans showed that the faster longitudinal shortening of telomere length (TL) was associated with subpar sleep quality, including short sleep duration, prolonged time to fall asleep, and inefficient sleep (Jin et al., 2022).

Sleep patterns have been thought to be influenced by age in previous studies, particularly changes in sleep duration (Neikrug & Ancoli‐Israel, 2010). There may be a bidirectional relationship between sleep duration and aging; however, our results do not provide evidence that short and long sleepers aged 60 or younger experienced accelerated PA, KDM, and increased HD, AL, but not in older adults. Moreover, the aging effects are almost independent of the genetic susceptibility for PA/KDM. According to a review of sleep disorders among elderly individuals, sleep disturbances are rare in healthy older adults (Neikrug & Ancoli‐Israel, 2010). Although it is not solely attributable to aging, the majority of sleep problems and disorders among the elderly coincide with medical and psychiatric illnesses and the associated health burdens (Vitiello et al., 2002).

Additionally, our study observed noteworthy distinctions in the association of long sleep duration with accelerated HD, KDM, PA, and AL. Just like epidemiological research that has consistently demonstrated an elevated risk of mortality associated with prolonged sleep. A systematic review of 17 studies also reported a significant relationship between longer sleep duration and higher HD (Christensen et al., 2022). Moreover, participants who sleep for longer tend to be associated with various other aging‐related phenotypes, which highlight the potential mechanisms that have been reported in previous studies for the detrimental health implications of excessive sleep, such as sleep fragmentation (Youngstedt & Kripke, 2004), persistent tiredness (Grandner & Kripke, 2004), or depressive symptoms (Tamakoshi & Ohno, 2004).

One additional revelation is that the associations of sleep duration with CysC and GGT displayed U‐shaped patterns, which illustrates the association between the imbalance of sleep duration and the body's susceptibility to external surroundings and internal fluctuations. The findings imply that controlling the impaired kidney and liver function that drives shorter and longer sleep duration may serve as a potential preventive measure against accelerated aging. Nonetheless, additional investigations are required to estimate other mechanisms.

The subgroup analyses particularly highlighted accelerated PA, KDM, and increased HD, AL in short and long sleepers aged 60 years and younger. This suggests the importance of ensuring adequate sleep for non‐elderly people. The cause could be a mixture of factors such as alterations to the circadian rhythm (Monk, 2005; Tranah et al., 2011), the impact of health conditions (Beard et al., 2016; Stenholm et al., 2015), and shifts in sleep requirements (Crowley, 2011; Scullin, 2013).

Even though aging can be influenced by diabetes (Lu et al., 2009), high blood pressure (Peng et al., 2017), and depression (Alexopoulos et al., 2002), the core relationship between imbalanced sleep duration and accelerated aging remained consistent even after considering these chronic conditions. Slow‐wave sleep (SWS) is believed to be responsible for various beneficial physiological processes during sleep. Epidemiological studies have demonstrated significant associations between both shorter and longer sleep durations and aberrant SWS, potentially accelerating cellular aging and diminishing tissue functionality (Landsness et al., 2009; Mander et al., 2017; Winer et al., 2021). Drastic changes in sleep may result in modifications in neurophysiological processes (Mander et al., 2017), including depression (Franzen & Buysse, 2008), Alzheimer's disease (Lim et al., 2013), and consequently, accelerated aging. Moreover, both short and long durations of sleep may be associated with disruptions in the endocrine system (Spiegel et al., 1999), encompassing human growth hormone (Takahashi et al., 1968), thyroid hormone, which are related to insufficient sleep, and sex hormones like testosterone (Luboshitzky et al., 2001), and estrogen, which are impacted by excessive sleep.

The major strengths of our study comprise the substantial sample size and sufficiently comprehensive phenotype and biochemistry data for the determination of predicted age metrics. Several noteworthy limitations should be considered when examining the findings. Firstly, sleep duration was provided by participants at baseline, with a single self‐reported measure. Consequently, it was unavoidable that there would be misclassification, and any alterations or enduring routines in sleep duration might not have been collected. Secondly, the blood‐chemistry‐based measures for HD, PA, and KDM developed through NHANES III data training may be constrained by sample and method, possibly affecting the application of these models for measuring parameters in the UK Biobank cohort. Thirdly, researchers were unable to completely exclude the potential for reverse causation. Although we performed various sensitivity analyses, including stratified analyses of genetic susceptibility for PA/KDM, a Mendelian Randomization analysis has the potential to yield more accurate findings regarding the relationship between aging and abnormal duration. Fourthly, the UK Biobank does not accurately reflect the population sample (Fry et al., 2017), which may have biases originating from “voluntarily participating and relatively healthy individuals.” Fifthly, there was a lack of available data on the utilization of medications that may influence the duration of sleep and potentially be associated with accelerated biological aging (Virta et al., 2013). Finally, it is crucial to acknowledge that the design limitations of observational studies preclude us from establishing a direct causal relationship between sleep duration and the onset of biological aging.

## 5 CONCLUSIONS

Both short and long sleep duration are associated with accelerated biological aging mediated by CysC and GGT, especially in populations that are not considered elderly. Our findings emphasize the independent role of appropriate sleep duration in the process of aging and advocate for effective and safe approaches to sleep‐related problems.

## AUTHOR CONTRIBUTIONS

X.W., X.Y., and Y.L. were accountable for the study's design and were granted complete access to all the data. X.W., M.L., and L.C. conducted the statistical analysis and contributed to the interpretation of the findings. W.W. and X.Q. took part in the preparation of the data and reviewed the manuscript. X.W. and J.Z. authored the manuscript and took primary responsibility for its final content. X.W., S.P., and X.X. visualized the results and conducted the comprehensive repetition and validation of the analysis. All authors were actively involved in and carefully examined and authorized the final draft of the manuscript.

## FUNDING INFORMATION

All authors were funded by the National Natural Science Foundation (82030100) and the National Key Research and Development Program of China (2022YFC2010105).

## CONFLICT OF INTEREST STATEMENT

All authors disclosed no conflicts of interest.

## Supporting information


Appendix S1

## Data Availability

The data can be found in a public, open‐access repository. This investigation utilized the UK Biobank resource with application number 103547. Access to the UK Biobank data is possible by applying through the UK Biobank website (www.biobank.ac.uk/).
