# Supplementary material for: U‐shaped association between sleep duration and biological aging: Evidence from the UK Biobank study
Source: Aging Cell. 2024 Mar 31;23(7):e14159. doi: 10.1111/acel.14159 (PMC11258478; doi:10.1111/acel.14159)
Supplement: Supplementary file 1 — Appendix S1 [file ACEL-23-e14159-s001.docx]

**Supplementary files**

**Supplementary table 1.** Components of predicted age metrics

**Supplementary table 2.** Genome-wide association signals for chronotype in participants of European ancestry from UK Biobank (n = 452,633)

**Supplementary table 3.** Genome-wide association signals for sleep duration in participants of European ancestry from the UK Biobank (n = 452,633)

**Supplementary table 4.** Genome-wide association signals for insomnia in participants of European ancestry from the UK Biobank (n = 452,633)

**Supplementary table 5.** Genome-wide association signals for daytime sleepiness in participants of European ancestry from the UK Biobank (n = 452,633)

**Supplementary table 6.** Genome-wide association signals for snoring in participants of European ancestry from the UK Biobank (n = 452,633)

**Supplementary table 7.** Genome-wide association signals for Klemera-Doubal method in participants of European ancestry from the UK Biobank (n = 452,633)

**Supplementary table 8.** Genome-wide association signals for phenoage in participants of European ancestry from the UK Biobank (n = 452,633)

**Supplementary table 9.** Associations of the odds of different sleep behaviors and predicted age metrics with polygenic risk scores at baseline

**Supplementary table 10.** Original questions and definitions for covariates

**Supplementary table 11.** Original questions for self-reported sleep characteristics

**Supplementary table 12.** Associations between sleep duration and predicted age metrics

**Supplementary table 13.** Associations between sleep duration and cystatin C (CysC) and gamma glutamyltransferase (GGT)

**Supplementary table 14.** Associations between sleep duration and predicted age metrics among participants who were not employed or employed without night shifts at baseline

**Supplementary table 15.** Associations between sleep duration and predicted age metrics among participants who were not depression at baseline

**Supplementary table 16.** Associations between sleep duration and predicted age metrics among participants without sleep disorder at baseline

**Supplementary table 17.** Associations between sleep duration and predicted age metrics among participants who did not self-report poor health at baseline

**Supplementary table 18.** Stratified analysis of the associations between sleep duration and homeostatic dysregulation (HD)

**Supplementary table 19.** Stratified analysis of the associations between sleep duration and PA residual

**Supplementary table 20.** Stratified analysis of the associations between sleep duration and KDM residual

**Supplementary table 21.** Stratified analysis of the associations between sleep duration and allostatic load (AL)

**Supplementary figure 1.** Correlation matrix of chronological age and predicted age metrics (Pearson correlation)

**Supplementary figure 2.** Associations between sleep duration and predicted age metrics among participants with PRSs for different predicted age metrics at baseline

**Supplementary figure 3.** Smoothing curve for the association of sleep duration expressed as hours/day with CysC and GGT

**Supplementary figure 4.** Effects mediated by CysC and GGT on the relationship between sleep duration and predicted age metrics

**Supplementary table 1.** Components of predicted age metrics^a^

|  | Habitual sleep duration, h/day | | | | | *P* | P*_test_* |
| --- | --- | --- | --- | --- | --- | --- | --- |
|  | ≤ 5 (n = 10,641) | 6 (n = 43,154) | 7 (n = 95,469) | 8 (n = 73,640) | ≥ 9 (n = 18,809) |  |  |
| Albumin, g/L | 45.11 (45.06, 45.16) | 45.30 (45.27, 45.32) | 45.40 (45.38, 45.41) | 45.28 (45.26, 45.30) | 45.05 (45.01, 45.09) | < 0.001 | < 0.001 |
| Alkaline phosphatase, U/L | 87.32 (86.75, 87.88) | 83.71 (83.47, 83.94) | 81.43 (81.28, 81.58) | 82.43 (82.25, 82.62) | 85.48 (85.08, 85.87) | < 0.001 | < 0.001 |
| C-reactive protein, mg/L | 3.07 (2.98, 3.16) | 2.59 (2.55, 2.63) | 2.29 (2.27, 2.31) | 2.47 (2.44, 2.50) | 3.03 (2.96, 3.10) | < 0.001 | < 0.001 |
| Total cholesterol, mmol/L | 5.69 (5.67, 5.71) | 5.71 (5.70, 5.72) | 5.70 (5.69, 5.71) | 5.71 (5.71, 5.72) | 5.67 (5.65, 5.68) | < 0.001 | 0.746 |
| Creatinine, umol/L | 72.19 (71.76, 72.61) | 72.47 (72.32, 72.62) | 72.58 (72.48, 72.68) | 72.61 (72.48, 72.75) | 73.99 (73.63, 74.36) | < 0.001 | < 0.001 |
| Glycated hemoglobin, % | 5.53 (5.51, 5.54) | 5.45 (5.44, 5.46) | 5.40 (5.40, 5.40) | 5.42 (5.41, 5.42) | 5.52 (5.51, 5.53) | < 0.001 | < 0.001 |
| Systolic blood pressure, mmHg | 138.40 (138.05, 138.75) | 137.45 (137.28, 137.62) | 136.97 (136.85, 137.09) | 137.87 (137.74, 138.01) | 139.20 (138.93, 139.47) | < 0.001 | < 0.001 |
| Uric acid, umol/L | 313.60 (312.02, 315.18) | 313.59 (312.83, 314.34) | 308.91 (308.41, 309.42) | 308.06 (307.48, 308.64) | 315.42 (314.23, 316.60) | < 0.001 | < 0.001 |
| Lymphocyte percent, % | 28.39 (28.25, 28.54) | 28.79 (28.72, 28.86) | 28.91 (28.86, 28.96) | 28.82 (28.77, 28.87) | 28.23 (28.12, 28.33) | < 0.001 | < 0.001 |
| White blood cell count, 10^9 cells/L | 7.13 (7.09, 7.17) | 6.92 (6.90, 6.93) | 6.79 (6.78, 6.80) | 6.86 (6.85, 6.88) | 7.10 (7.08, 7.13) | < 0.001 | < 0.001 |

^a^All variables were listed as mean (95% CI). One-way ANOVA was conducted to compare components of predicted age metrics. And *P*_test_ was the result of Bonfreni correction.

**Supplementary table 2.** Genome-wide association signals for chronotype in participants of European ancestry from UK Biobank (n = 452,633)^a^

| CHR | BP | SNP | A1 | A2 | N | SE | P | OR |
| --- | --- | --- | --- | --- | --- | --- | --- | --- |
| 1 | 4850823 | rs909757 | T | C | 449734 | 0.00278157 | 0.00000046 | 1.013756258 |
| 1 | 7884525 | rs61773390 | T | G | 449734 | 0.00337553 | 1.2E-23 | 1.034666549 |
| 1 | 14507831 | rs12065331 | T | C | 449734 | 0.002934 | 0.000000067 | 0.984203888 |
| 1 | 15966713 | rs17448682 | T | C | 449734 | 0.00318247 | 2.2E-12 | 1.022399995 |
| 1 | 20006887 | rs10917513 | T | C | 449734 | 0.00282417 | 6.7E-10 | 0.982256896 |
| 1 | 21201325 | rs10916892 | T | C | 449734 | 0.00278123 | 6.1E-13 | 0.980202104 |
| 1 | 24321935 | rs2506089 | T | G | 449734 | 0.00270899 | 0.000064 | 1.010573509 |
| 1 | 62579891 | rs12140153 | T | G | 449734 | 0.00468803 | 1.6E-13 | 0.966560969 |
| 1 | 66851147 | rs11208844 | A | G | 449734 | 0.00389365 | 0.0000013 | 0.981076148 |
| 1 | 77705365 | rs12040629 | A | G | 449734 | 0.00364815 | 6.7E-27 | 1.039932804 |
| 1 | 79963816 | rs11588913 | A | G | 449734 | 0.00273856 | 0.000000015 | 0.984666277 |
| 1 | 81672013 | rs5016898 | T | C | 449734 | 0.00272868 | 0.0000058 | 0.987822157 |
| 1 | 91191582 | rs72720396 | A | G | 449734 | 0.00318521 | 8.4E-18 | 0.973238508 |
| 1 | 93469865 | rs481214 | A | T | 449734 | 0.00274208 | 0.00000087 | 1.013312229 |
| 1 | 96959104 | rs11165655 | A | G | 449734 | 0.00268854 | 4.1E-09 | 0.984406458 |
| 1 | 110086451 | rs17575798 | A | G | 449734 | 0.00339377 | 4.2E-11 | 0.977697169 |
| 1 | 113188419 | rs6690292 | T | C | 449734 | 0.00301214 | 0.0000045 | 0.986636693 |
| 1 | 115061584 | rs11102807 | A | G | 449734 | 0.00270365 | 0.00000021 | 0.987018595 |
| 1 | 150467753 | rs9436119 | A | G | 449734 | 0.00273835 | 4E-18 | 1.023767961 |
| 1 | 153756083 | rs6665637 | A | G | 449734 | 0.00304959 | 0.00000062 | 0.984914937 |
| 1 | 174215858 | rs115073088 | A | G | 449734 | 0.00893134 | 3.1E-09 | 0.948873488 |
| 1 | 179338327 | rs975025 | T | C | 449734 | 0.00502525 | 1.3E-10 | 0.968403732 |
| 1 | 182569626 | rs1144566 | T | C | 449734 | 0.00783578 | 5.8E-47 | 1.120042913 |
| 1 | 190095126 | rs146820337 | D | I | - | - | - | - |
| 1 | 193276975 | rs1221502 | A | C | 449734 | 0.00305065 | 0.00000083 | 1.01510738 |
| 1 | 195454557 | rs4657983 | A | G | 449734 | 0.00282135 | 0.0000015 | 0.986537146 |
| 1 | 241137033 | rs6429233 | A | G | 449734 | 0.00270164 | 0.0000021 | 1.013000481 |
| 2 | 4651923 | rs13011556 | C | G | 449734 | 0.00316075 | 1.1E-12 | 0.977514259 |
| 2 | 12822995 | rs62124718 | A | G | 449734 | 0.00436084 | 0.00000012 | 0.977497153 |
| 2 | 24180078 | rs72796401 | A | T | 449734 | 0.00343008 | 5.9E-11 | 1.022200953 |
| 2 | 25122324 | rs6718511 | A | G | 449734 | 0.00269385 | 0.000015 | 1.012026544 |
| 2 | 32563426 | rs11678584 | A | T | 449734 | 0.00389371 | 0.000028 | 0.983105048 |
| 2 | 36700580 | rs848552 | C | G | 449734 | 0.00268579 | 0.000000007 | 0.984794883 |
| 2 | 41385367 | rs7602499 | T | C | 449734 | 0.00283306 | 0.000032 | 1.011986064 |
| 2 | 44271496 | rs75120545 | T | C | 449734 | 0.00828587 | 1.1E-13 | 1.063315333 |
| 2 | 46863872 | rs6544906 | A | C | 449734 | 0.00270644 | 0.000011 | 1.011991832 |
| 2 | 48252311 | rs17396357 | T | C | 449734 | 0.00277137 | 0.00061 | 1.009363819 |
| 2 | 50532840 | rs12470914 | A | T | 449734 | 0.00443933 | 2.2E-09 | 1.026462782 |
| 2 | 53736362 | rs4672458 | T | C | 449734 | 0.00268601 | 1.1E-09 | 0.983860756 |
| 2 | 54275162 | rs13414393 | T | C | 449734 | 0.002697 | 0.00000081 | 0.9867102 |
| 2 | 59429807 | rs10175975 | T | C | 449734 | 0.00351376 | 1.8E-10 | 1.023107025 |
| 2 | 60477461 | rs359248 | T | G | 449734 | 0.00270985 | 1.5E-09 | 0.983977941 |
| 2 | 61680993 | rs812925 | C | G | 449734 | 0.00280786 | 6.9E-13 | 0.979645899 |
| 2 | 66750564 | rs113851554 | T | G | 449734 | 0.00598996 | 0.000000001 | 0.964895668 |
| 2 | 70488470 | rs2706762 | T | C | 449734 | 0.00375432 | 0.000000004 | 0.978316736 |
| 2 | 75445544 | rs12464387 | A | G | 449734 | 0.0026961 | 0.00000047 | 0.986424391 |
| 2 | 76361783 | rs6727752 | A | G | 449734 | 0.00277464 | 0.000000014 | 1.015818509 |
| 2 | 77217310 | rs10520176 | T | C | 449734 | 0.00268932 | 1.4E-17 | 1.023419836 |
| 2 | 88901732 | rs11681299 | T | C | 449734 | 0.00296094 | 0.00027 | 1.011171035 |
| 2 | 101591710 | rs34509802 | A | G | 449734 | 0.00349675 | 0.0000053 | 1.015483953 |
| 2 | 125438641 | rs76064513 | T | C | 449734 | 0.00405038 | 0.00015 | 1.015475017 |
| 2 | 136490731 | rs77248969 | A | G | 449734 | 0.00470922 | 0.0000018 | 0.978424064 |
| 2 | 144232491 | rs28380327 | A | T | 449734 | 0.00277698 | 2.9E-13 | 1.020721469 |
| 2 | 149551658 | rs2166559 | T | C | 449734 | 0.00387088 | 9.1E-09 | 0.978403028 |
| 2 | 161916409 | rs747003 | T | C | 449734 | 0.00275473 | 0.00000027 | 1.014701926 |
| 2 | 174037347 | rs13004345 | T | C | 449734 | 0.00281783 | 0.000002 | 0.98684825 |
| 2 | 175241482 | rs6433478 | T | C | 449734 | 0.00271259 | 0.0000053 | 0.988065587 |
| 2 | 186203743 | rs4666682 | A | G | 449734 | 0.00351886 | 0.000001 | 0.982841316 |
| 2 | 191578172 | rs11677484 | T | G | 449734 | 0.00309256 | 1.7E-09 | 1.01872964 |
| 2 | 198950240 | rs1064213 | A | G | 449734 | 0.00268602 | 1.3E-12 | 1.019325363 |
| 2 | 206956138 | rs184033703 | A | G | 449734 | 0.00576165 | 8.1E-11 | 1.038516965 |
| 2 | 239311505 | rs80271258 | T | C | 449734 | 0.00476547 | 2.6E-30 | 0.946564277 |
| 2 | 240267305 | rs62182135 | A | C | 449734 | 0.00284795 | 3.7E-10 | 0.982216133 |
| 3 | 2521322 | rs35346733 | A | G | 449734 | 0.00341396 | 0.00000045 | 0.982569402 |
| 3 | 7189617 | rs111261826 | A | C | 449734 | 0.00287969 | 0.00000048 | 0.985756611 |
| 3 | 8817423 | rs149611468 | T | C | 449734 | 0.0125983 | 8.5E-10 | 1.080730715 |
| 3 | 14383632 | rs6794796 | A | G | 449734 | 0.00296536 | 5.3E-09 | 1.017366677 |
| 3 | 18246870 | rs9817910 | A | G | 449734 | 0.00270846 | 3.1E-10 | 0.983107211 |
| 3 | 23224684 | rs73050286 | T | C | 449734 | 0.00326167 | 0.000000026 | 1.017969035 |
| 3 | 24924421 | rs2362775 | T | C | 449734 | 0.0027019 | 2.8E-11 | 0.982142764 |
| 3 | 36859494 | rs114848860 | A | T | 449734 | 0.00872612 | 3.6E-10 | 0.945932846 |
| 3 | 46986452 | rs78580841 | T | C | 449734 | 0.0053556 | 3.7E-09 | 1.031422791 |
| 3 | 50003323 | rs12636669 | T | C | 449734 | 0.00497499 | 5.8E-12 | 1.034941393 |
| 3 | 70594975 | rs17007397 | C | G | 449734 | 0.00272422 | 0.000072 | 1.010910894 |
| 3 | 71575177 | rs7626335 | A | C | 449734 | 0.0028667 | 0.00003 | 0.988600275 |
| 3 | 77205438 | rs7429614 | T | G | 449734 | 0.00272259 | 9.2E-10 | 1.016552601 |
| 3 | 82591379 | rs112201801 | T | C | - | - | - | - |
| 3 | 83804561 | rs12631477 | T | C | 449734 | 0.00336338 | 0.00000079 | 1.017025408 |
| 3 | 85591467 | rs1449403 | A | G | 449734 | 0.00406748 | 0.00000061 | 1.019864525 |
| 3 | 104778430 | rs34967119 | A | G | 449734 | 0.00268499 | 0.0000038 | 1.012800433 |
| 3 | 110271943 | rs1398346 | T | C | 449734 | 0.00402359 | 0.000015 | 1.017688928 |
| 3 | 113891549 | rs1800828 | C | G | 449734 | 0.00308462 | 0.000000017 | 1.01777971 |
| 3 | 116103275 | rs72950188 | T | C | 449734 | 0.00508928 | 0.000002 | 1.024378513 |
| 3 | 123149816 | rs72966564 | T | C | 449734 | 0.00312692 | 0.000000025 | 0.982714046 |
| 3 | 132971327 | rs13065394 | T | G | 449734 | 0.00296404 | 0.000000003 | 0.982648305 |
| 3 | 138132393 | rs4550782 | T | G | 449734 | 0.00285144 | 0.000000022 | 1.015922432 |
| 3 | 150788032 | rs7649164 | T | G | 449734 | 0.00277539 | 0.0000023 | 1.013526567 |
| 3 | 152646244 | rs6440833 | A | G | 449734 | 0.00270216 | 0.00001 | 1.012097793 |
| 3 | 157721819 | rs111867612 | A | C | 449734 | 0.00442418 | 0.0000019 | 0.979549213 |
| 3 | 160891727 | rs1599374 | A | G | 449734 | 0.00270337 | 0.000000015 | 1.015499592 |
| 3 | 172364093 | rs3850174 | A | T | 449734 | 0.00309938 | 6.8E-10 | 0.980740775 |
| 3 | 176096919 | rs301218 | A | G | 449734 | 0.00274639 | 0.00001 | 0.987876292 |
| 3 | 182096311 | rs9836621 | T | C | 449734 | 0.00269338 | 0.0000048 | 0.987377441 |
| 3 | 185990392 | rs1468945 | A | G | 449734 | 0.00326992 | 4.2E-12 | 0.977667252 |
| 4 | 1349602 | rs3796618 | A | T | 449734 | 0.00269667 | 0.00000055 | 0.986819632 |
| 4 | 2697300 | rs4690085 | A | G | 449734 | 0.00269058 | 0.000000039 | 0.985369482 |
| 4 | 18260776 | rs4698678 | C | G | 449734 | 0.00298441 | 0.0000027 | 1.014142472 |
| 4 | 27495379 | rs1502249 | A | G | 449734 | 0.0026996 | 0.0000048 | 1.012272294 |
| 4 | 66520667 | rs6838677 | A | C | 449734 | 0.00285778 | 0.0000028 | 0.986723126 |
| 4 | 67096904 | rs4860734 | A | G | 449734 | 0.00301611 | 0.0000068 | 1.013783529 |
| 4 | 80206272 | rs6816922 | A | C | 449734 | 0.00269893 | 0.000012 | 0.988509822 |
| 4 | 83279041 | rs6846730 | T | C | 449734 | 0.00317897 | 5.6E-12 | 0.978238572 |
| 4 | 102094764 | rs2850979 | T | C | 449734 | 0.00315303 | 0.000000079 | 0.983471028 |
| 4 | 105317995 | rs10610420 | D | I | - | - | - | - |
| 4 | 114439894 | rs7700110 | A | G | 449734 | 0.00306615 | 0.000013 | 1.013452785 |
| 4 | 130903511 | rs17455138 | T | C | 449734 | 0.00319267 | 0.000035 | 1.01311445 |
| 4 | 132512118 | rs9991917 | A | T | - | - | - | - |
| 4 | 137053959 | rs4241964 | T | G | 449734 | 0.00270449 | 1.1E-14 | 0.979478492 |
| 4 | 139939653 | rs938836 | A | G | 449734 | 0.00269582 | 0.0000025 | 0.987039717 |
| 4 | 147296930 | rs72729847 | T | C | 449734 | 0.00338436 | 0.0000017 | 0.984268257 |
| 4 | 163704083 | rs9997394 | A | G | 449734 | 0.00296075 | 0.0000083 | 0.986452505 |
| 5 | 35220404 | rs10058356 | T | C | 449734 | 0.00293612 | 6.8E-09 | 0.983161676 |
| 5 | 59027048 | rs67169439 | D | I | - | - | - | - |
| 5 | 63861475 | rs7701529 | A | T | 449734 | 0.00317247 | 3.6E-09 | 0.981331653 |
| 5 | 76581258 | rs7721608 | T | G | 449734 | 0.00269597 | 0.000042 | 1.011444796 |
| 5 | 86630284 | rs66507804 | T | C | 449734 | 0.00332753 | 0.00000093 | 0.983523645 |
| 5 | 87701223 | rs4269995 | T | C | 449734 | 0.00309061 | 5.9E-12 | 0.978953632 |
| 5 | 103964585 | rs77960 | A | G | 449734 | 0.00286325 | 4.2E-11 | 1.018933712 |
| 5 | 106657015 | rs1559253 | A | G | 449734 | 0.00282129 | 0.00077 | 1.009758931 |
| 5 | 115939896 | rs17140201 | A | G | 449734 | 0.00362764 | 0.00000012 | 0.980799033 |
| 5 | 122990902 | rs13172141 | A | T | 449734 | 0.00271044 | 0.0000082 | 1.012239801 |
| 5 | 152204741 | rs67988891 | C | G | 449734 | 0.00288939 | 3.7E-15 | 0.977027871 |
| 5 | 163330708 | rs2901796 | A | G | 449734 | 0.00275148 | 0.0000007 | 1.013587583 |
| 5 | 166408788 | rs42210 | C | G | 449734 | 0.0029834 | 0.000003 | 0.986394306 |
| 5 | 173539588 | rs12518401 | A | G | 449734 | 0.00284697 | 0.000007 | 0.987545507 |
| 5 | 175339984 | rs7735794 | A | G | 449734 | 0.00340433 | 4.7E-09 | 1.020318364 |
| 5 | 176877624 | rs465670 | T | C | 449734 | 0.00269725 | 0.000000063 | 1.014616491 |
| 6 | 11574374 | rs9394154 | C | G | 449734 | 0.00271526 | 0.000000013 | 0.984616848 |
| 6 | 12155114 | rs34125199 | D | I | - | - | - | - |
| 6 | 13183998 | rs9381812 | A | G | 449734 | 0.00294635 | 4.6E-15 | 0.977209713 |
| 6 | 14878060 | rs1811899 | T | C | 449734 | 0.00331758 | 0.00004 | 0.986258883 |
| 6 | 19102247 | rs9465253 | T | C | 449734 | 0.00300229 | 0.0000014 | 1.014497281 |
| 6 | 26319588 | rs766406 | T | G | 449734 | 0.00278612 | 0.000071 | 0.988587918 |
| 6 | 31856070 | rs486416 | A | G | 449734 | 0.00277852 | 0.000000023 | 0.984690008 |
| 6 | 37630133 | rs13203140 | T | C | 449734 | 0.00279966 | 0.000011 | 0.987254915 |
| 6 | 38440970 | rs3923809 | A | G | 449734 | 0.00292127 | 0.00011 | 0.98863636 |
| 6 | 41517457 | rs12206814 | C | G | 449734 | 0.00273257 | 0.000000028 | 1.015290725 |
| 6 | 43355851 | rs2396004 | A | G | 449734 | 0.00270757 | 0.0000043 | 1.012550606 |
| 6 | 50938247 | rs3857599 | A | C | 449734 | 0.00363894 | 1.4E-09 | 1.022092708 |
| 6 | 55142337 | rs2653349 | A | G | 449734 | 0.00327448 | 5.2E-32 | 1.039484482 |
| 6 | 57767576 | rs9476310 | T | C | 449734 | 0.00269436 | 0.000000075 | 1.014757228 |
| 6 | 62589167 | rs1931814 | A | G | 449734 | 0.00268976 | 0.000000016 | 1.0155067 |
| 6 | 72479263 | rs2881955 | T | C | 449734 | 0.00300229 | 0.000000043 | 1.016252254 |
| 6 | 98705295 | rs12195792 | A | T | 449734 | 0.00303318 | 0.00000067 | 1.015011254 |
| 6 | 99592404 | rs11154718 | T | C | 449734 | 0.00272294 | 0.0000058 | 0.987938629 |
| 6 | 110244765 | rs60616179 | A | G | 449734 | 0.00594669 | 1.1E-10 | 1.038295992 |
| 6 | 115699280 | rs4535583 | T | C | 449734 | 0.00291927 | 0.000039 | 1.012278267 |
| 6 | 143751625 | rs9496623 | A | G | 449734 | 0.00316234 | 0.0000023 | 0.984948129 |
| 6 | 147936781 | rs2050185 | A | G | 449734 | 0.00279246 | 0.000000089 | 1.015036426 |
| 6 | 153135339 | rs9479402 | T | C | 449734 | 0.0131937 | 3.2E-16 | 0.898578688 |
| 6 | 165195547 | rs9347926 | A | T | 449734 | 0.00269893 | 7.3E-09 | 1.0158308 |
| 6 | 166263488 | rs9348050 | T | C | 449734 | 0.00269048 | 7.5E-10 | 1.016777182 |
| 7 | 14093914 | rs4027217 | A | C | 449734 | 0.00328423 | 0.000003 | 0.985076575 |
| 7 | 24085405 | rs10237162 | T | C | 449734 | 0.00301354 | 3.5E-09 | 1.017773807 |
| 7 | 32265545 | rs10951325 | T | C | 449734 | 0.00280238 | 7.9E-14 | 1.02081048 |
| 7 | 50642701 | rs6967481 | T | C | 449734 | 0.00269925 | 6.9E-15 | 1.020857847 |
| 7 | 69936477 | rs4236237 | A | C | 449734 | 0.00273991 | 0.0000028 | 0.987243759 |
| 7 | 71779635 | rs2944831 | A | G | 449734 | 0.00295094 | 0.000000011 | 1.017390891 |
| 7 | 77823771 | rs3807651 | A | T | 449734 | 0.00269908 | 0.0000014 | 1.012993998 |
| 7 | 96468077 | rs10254050 | C | G | 449734 | 0.00344351 | 1.2E-12 | 0.975669185 |
| 7 | 102383663 | rs4729854 | A | T | 449734 | 0.00275036 | 3.3E-25 | 0.97168986 |
| 7 | 113893884 | rs2396719 | A | G | 449734 | 0.00312251 | 0.000000066 | 1.017185806 |
| 7 | 115673079 | rs17302081 | T | C | 449734 | 0.00270471 | 6.9E-09 | 1.015780518 |
| 7 | 121942674 | rs6968240 | A | C | 449734 | 0.00272987 | 0.000018 | 1.011880823 |
| 7 | 132294312 | rs62465218 | A | C | 449734 | 0.00377821 | 0.000042 | 0.984873867 |
| 7 | 133585794 | rs6958557 | T | G | 449734 | 0.00275105 | 0.0000017 | 1.012792432 |
| 7 | 148564367 | rs113161209 | A | G | 449734 | 0.00531193 | 0.0000045 | 1.024853833 |
| 7 | 150647969 | rs2072413 | T | C | 449734 | 0.00303599 | 0.0000035 | 0.985909809 |
| 8 | 3654320 | rs62479736 | T | G | 449734 | 0.00295184 | 0.00000012 | 1.015313265 |
| 8 | 4823608 | rs35524253 | A | G | 449734 | 0.00281259 | 5.8E-09 | 1.015988469 |
| 8 | 8268313 | rs2979139 | A | G | 449734 | 0.00269315 | 7.4E-10 | 0.983340038 |
| 8 | 27164449 | rs2322605 | A | G | 449734 | 0.00268932 | 0.0000025 | 0.987494749 |
| 8 | 31817493 | rs71523448 | C | G | 449734 | 0.00505523 | 0.00000002 | 0.972363766 |
| 8 | 33729200 | rs6993892 | T | C | 449734 | 0.00275662 | 8.4E-14 | 0.979692825 |
| 8 | 35237788 | rs6468316 | T | C | 449734 | 0.00268825 | 0.000000059 | 0.985290064 |
| 8 | 53129069 | rs7845620 | A | C | 449734 | 0.00363166 | 5.2E-10 | 0.977763655 |
| 8 | 59800446 | rs10109566 | A | G | 449734 | 0.00270003 | 0.0000015 | 0.986973686 |
| 8 | 65015659 | rs34054660 | A | G | 449734 | 0.00271775 | 0.0022 | 1.008768601 |
| 8 | 73459513 | rs187028 | A | T | 449734 | 0.00289662 | 0.00000031 | 0.984992256 |
| 8 | 76653156 | rs16939162 | A | G | 449734 | 0.00358356 | 2.2E-12 | 1.025303535 |
| 8 | 91535686 | rs6988733 | T | C | 449734 | 0.00282228 | 0.00002 | 1.0122232 |
| 8 | 93283578 | rs7006885 | A | G | 449734 | 0.00297063 | 0.00094 | 1.010071075 |
| 8 | 101967139 | rs3100052 | A | G | 449734 | 0.00276058 | 6.9E-10 | 1.017226189 |
| 8 | 116658583 | rs2737245 | T | G | 449734 | 0.00300138 | 4.5E-13 | 1.02173984 |
| 8 | 136223702 | rs1871729 | A | G | 449734 | 0.00288364 | 0.0000072 | 0.987526645 |
| 9 | 8450638 | rs6477309 | T | C | 449734 | 0.00286276 | 8E-10 | 1.017592863 |
| 9 | 24582747 | rs2844016 | T | C | 449734 | 0.00294187 | 0.000013 | 1.013257917 |
| 9 | 37367094 | rs308521 | T | C | 449734 | 0.00275427 | 3.7E-13 | 1.020172571 |
| 9 | 38010085 | rs4878734 | A | T | 449734 | 0.00268872 | 0.00000037 | 1.01399168 |
| 9 | 74245426 | rs6560218 | T | C | 449734 | 0.00271548 | 0.0000013 | 0.98725689 |
| 9 | 76679777 | rs62553781 | T | C | 449734 | 0.00734656 | 1.2E-11 | 0.950848628 |
| 9 | 83196097 | rs12378543 | T | C | 449734 | 0.00276985 | 0.00000066 | 0.986616269 |
| 9 | 85318704 | rs555784 | A | T | 449734 | 0.00276767 | 0.0000018 | 0.987050673 |
| 9 | 86429305 | rs295268 | T | C | 449734 | 0.00307939 | 0.000000041 | 0.983548528 |
| 9 | 92219000 | rs3138490 | A | T | 449734 | 0.00271195 | 0.00000019 | 1.013988131 |
| 9 | 109806199 | rs10759208 | T | C | 449734 | 0.00276525 | 0.0000006 | 0.986853974 |
| 9 | 116767656 | rs11788633 | C | G | 449734 | 0.00282602 | 0.00004 | 1.011737146 |
| 9 | 126317324 | rs10818834 | T | C | 449734 | 0.00304915 | 1.1E-09 | 1.019046412 |
| 9 | 131943440 | rs10988239 | T | C | 449734 | 0.00273304 | 1.6E-11 | 0.981539129 |
| 9 | 139310187 | rs12380242 | T | C | 449734 | 0.0026864 | 1.6E-09 | 0.984328103 |
| 9 | 140257189 | rs28458909 | T | C | 449734 | 0.00407749 | 2.6E-26 | 0.957642925 |
| 10 | 804315 | rs497338 | T | C | 449734 | 0.00298801 | 0.00000069 | 1.015044141 |
| 10 | 56699338 | rs66617308 | T | C | 449734 | 0.00286063 | 0.00000023 | 1.014522035 |
| 10 | 60567937 | rs9416744 | A | C | 449734 | 0.00308075 | 1.3E-09 | 1.019165648 |
| 10 | 61794146 | rs11597421 | A | G | 449734 | 0.00273785 | 0.000025 | 0.988124181 |
| 10 | 64301941 | rs12249410 | T | G | 449734 | 0.00438012 | 0.000000064 | 0.976584401 |
| 10 | 69623271 | rs17712705 | A | G | 449734 | 0.00286003 | 0.000014 | 0.987482899 |
| 10 | 70346740 | rs2298117 | T | C | 449734 | 0.00270147 | 0.000000051 | 0.985026534 |
| 10 | 73044413 | rs10762434 | C | G | 449734 | 0.00323034 | 0.00011 | 1.012440345 |
| 10 | 93026996 | rs2648721 | T | G | 449734 | 0.00293525 | 0.00000091 | 0.985456198 |
| 10 | 93888810 | rs61875203 | T | C | 449734 | 0.00300062 | 0.0000016 | 1.014609288 |
| 10 | 104943993 | rs1163238 | A | G | 449734 | 0.00275048 | 0.00000061 | 0.986548886 |
| 10 | 119145774 | rs7900191 | T | C | 449734 | 0.00276251 | 0.0000008 | 0.986165982 |
| 10 | 123553392 | rs11200159 | A | C | 449734 | 0.00283041 | 0.00000047 | 0.985946486 |
| 10 | 125426627 | rs3808964 | T | G | 449734 | 0.00279601 | 0.000000027 | 1.015833644 |
| 10 | 126710791 | rs9664044 | T | C | 449734 | 0.00318672 | 0.00000016 | 0.983291069 |
| 10 | 129304075 | rs10830107 | A | G | 449734 | 0.00339169 | 0.0000072 | 1.014975019 |
| 10 | 131149976 | rs76518095 | T | C | 449734 | 0.00505367 | 0.0000042 | 1.024061005 |
| 10 | 133749294 | rs12771973 | A | G | 449734 | 0.00309542 | 0.000013 | 0.986530339 |
| 11 | 13314102 | rs60521023 | D | I | - | - | - | - |
| 11 | 16618307 | rs10832648 | A | C | 449734 | 0.00338271 | 3.2E-09 | 0.980389831 |
| 11 | 27650524 | rs10742179 | A | G | 449734 | 0.00306465 | 1.5E-09 | 1.01846511 |
| 11 | 28479535 | rs4923541 | T | C | 449734 | 0.00268691 | 0.0000046 | 1.01250079 |
| 11 | 30405914 | rs621421 | T | C | 449734 | 0.00277678 | 7.5E-11 | 0.982416919 |
| 11 | 33759092 | rs11032362 | A | G | 449734 | 0.00467579 | 4.8E-17 | 1.040346468 |
| 11 | 43893222 | rs7111582 | A | G | 449734 | 0.00442599 | 5.2E-10 | 0.972628187 |
| 11 | 47312892 | rs10838687 | T | G | 449734 | 0.00329533 | 0.00000038 | 1.016720448 |
| 11 | 58373221 | rs12808544 | A | C | 449734 | 0.00314754 | 3E-11 | 0.979461351 |
| 11 | 66342691 | rs662094 | A | G | 449734 | 0.00268945 | 0.00000071 | 1.013668167 |
| 11 | 82972097 | rs1278402 | A | G | 449734 | 0.0030443 | 0.00028 | 1.011077001 |
| 11 | 92893825 | rs1508608 | A | G | 449734 | 0.00287547 | 0.0000032 | 1.013395223 |
| 11 | 95120372 | rs4121878 | C | G | 449734 | 0.00268747 | 0.0000048 | 1.012182813 |
| 11 | 99152801 | rs17577073 | A | C | 449734 | 0.0027333 | 0.0000013 | 1.013515317 |
| 11 | 113395329 | rs2514214 | A | G | 449734 | 0.00303738 | 0.00000059 | 1.015419878 |
| 11 | 114009255 | rs4936290 | A | C | 449734 | 0.00282752 | 4.6E-10 | 0.982450027 |
| 11 | 122093090 | rs3867239 | A | G | 449734 | 0.00277918 | 0.00000087 | 1.014065501 |
| 11 | 122811822 | rs74357745 | A | G | 449734 | 0.00412156 | 8.7E-11 | 1.026901484 |
| 11 | 126734319 | rs7943634 | T | C | 449734 | 0.00291003 | 0.000000014 | 0.983679644 |
| 12 | 361996 | rs3782860 | T | C | 449734 | 0.00270465 | 0.00000072 | 1.01329551 |
| 12 | 16286082 | rs1799464 | A | G | 449734 | 0.00296926 | 0.0000056 | 0.986561218 |
| 12 | 17015267 | rs12298405 | T | C | 449734 | 0.00286448 | 0.000000027 | 0.98427121 |
| 12 | 23060363 | rs2433634 | A | C | 449734 | 0.00298813 | 0.00000033 | 0.984786217 |
| 12 | 24089322 | rs11611435 | T | C | 449734 | 0.00270297 | 8.6E-09 | 1.015864831 |
| 12 | 34051765 | rs13377754 | T | C | 449734 | 0.00275916 | 6.9E-27 | 1.030175422 |
| 12 | 38737310 | rs1843888 | A | G | 449734 | 0.00270621 | 3.2E-26 | 1.029258665 |
| 12 | 46294908 | rs247929 | C | G | 449734 | 0.0026901 | 6.2E-11 | 1.017865411 |
| 12 | 49413486 | rs7975791 | T | C | 449734 | 0.00689894 | 0.00000029 | 1.035170347 |
| 12 | 52042142 | rs4761989 | T | C | 449734 | 0.00398363 | 0.000001 | 0.98104907 |
| 12 | 54702519 | rs7299922 | A | G | 449734 | 0.00279944 | 2.5E-09 | 1.0162801 |
| 12 | 57824165 | rs487722 | T | G | 449734 | 0.00329114 | 0.000000096 | 1.017614131 |
| 12 | 63520912 | rs10877962 | T | C | 449734 | 0.00273885 | 0.000000082 | 1.014651192 |
| 12 | 77976559 | rs711098 | A | C | 449734 | 0.00274587 | 0.0004 | 1.00968409 |
| 12 | 90452978 | rs7959983 | T | C | 449734 | 0.00272849 | 9.5E-13 | 0.980663594 |
| 12 | 106989915 | rs7304278 | A | G | 449734 | 0.00302264 | 2.2E-11 | 0.979948755 |
| 12 | 112510404 | rs7298532 | T | C | 449734 | 0.00298282 | 0.000000027 | 1.016566121 |
| 12 | 114343818 | rs3955311 | T | C | 449734 | 0.00376823 | 0.0000069 | 1.016904491 |
| 12 | 121029604 | rs80097534 | T | G | 449734 | 0.00455453 | 9.3E-10 | 0.972288702 |
| 13 | 56281271 | rs9597241 | A | C | 449734 | 0.00345609 | 7.5E-10 | 1.021618771 |
| 13 | 59576365 | rs12871550 | A | G | 449734 | 0.00287516 | 0.000000026 | 1.016125231 |
| 13 | 66590868 | rs9571526 | T | G | 449734 | 0.00319269 | 0.000016 | 0.98667162 |
| 13 | 69903058 | rs2593487 | A | G | 449734 | 0.00284306 | 0.00000025 | 0.985265629 |
| 13 | 72919800 | rs495593 | A | G | 449734 | 0.00308061 | 0.000031 | 1.012963203 |
| 13 | 73649152 | rs45597035 | A | G | 449734 | 0.00284536 | 0.0000034 | 0.987119572 |
| 13 | 77590741 | rs9573980 | A | G | 449734 | 0.00745314 | 5.7E-22 | 1.075417007 |
| 13 | 94062095 | rs1886205 | A | C | 449734 | 0.00316104 | 4.9E-10 | 1.019625293 |
| 13 | 107700218 | rs9558942 | T | C | 449734 | 0.00286731 | 0.00072 | 0.99017837 |
| 13 | 109779906 | rs3815983 | T | C | 449734 | 0.00282602 | 0.00000015 | 0.985284054 |
| 13 | 112226420 | rs1163628 | A | C | 449734 | 0.0038478 | 0.0000015 | 0.981335284 |
| 14 | 42069889 | rs61990287 | A | C | 449734 | 0.00299613 | 0.000003 | 1.013913808 |
| 14 | 55373670 | rs2878172 | A | G | 449734 | 0.00272883 | 0.000094 | 0.989640335 |
| 14 | 57281154 | rs962961 | T | C | 449734 | 0.00286872 | 0.00000002 | 0.984218947 |
| 14 | 60806976 | rs6573308 | T | C | 449734 | 0.00277713 | 0.00002 | 1.011978069 |
| 14 | 62460219 | rs7143933 | T | G | 449734 | 0.00305185 | 0.00012 | 1.011441256 |
| 14 | 64769074 | rs2978382 | T | C | 449734 | 0.00274141 | 0.00000061 | 1.014097648 |
| 14 | 74660508 | rs4903203 | A | G | 449734 | 0.00287892 | 0.00000017 | 1.014609896 |
| 14 | 79452019 | rs12436039 | T | C | 449734 | 0.00422728 | 0.00084 | 1.014437326 |
| 14 | 85350142 | rs4550384 | T | G | 449734 | 0.00313024 | 0.00000077 | 1.01584553 |
| 14 | 98532540 | rs710284 | T | C | 449734 | 0.00273187 | 0.000017 | 1.011984445 |
| 14 | 101016824 | rs11845599 | A | G | 449734 | 0.00281767 | 0.000000056 | 0.984827776 |
| 15 | 48009263 | rs59986227 | C | G | 449734 | 0.00310217 | 8.3E-09 | 0.982318092 |
| 15 | 53725112 | rs12442008 | T | C | 449734 | 0.00311391 | 0.000003 | 1.014396039 |
| 15 | 58969292 | rs4775086 | A | G | 449734 | 0.00316622 | 0.000016 | 0.986331869 |
| 15 | 96907819 | rs12442674 | A | C | 449734 | 0.00301088 | 0.00014 | 1.011325453 |
| 15 | 101147726 | rs1873958 | A | G | 449734 | 0.0027541 | 0.00000013 | 1.014217115 |
| 16 | 728514 | rs72773411 | A | G | 449734 | 0.003685 | 0.000023 | 1.015695297 |
| 16 | 8195278 | rs12445235 | C | G | 449734 | 0.00273708 | 0.0000068 | 0.987361248 |
| 16 | 8988777 | rs2304467 | C | G | 449734 | 0.00276055 | 0.00000022 | 0.985989572 |
| 16 | 23124193 | rs11641239 | T | C | 449734 | 0.00296753 | 0.000000011 | 1.017398216 |
| 16 | 24518569 | rs7203707 | A | C | 449734 | 0.00269903 | 7.6E-10 | 0.983662331 |
| 16 | 49467234 | rs4785296 | C | G | 449734 | 0.00318655 | 0.0000013 | 1.015291639 |
| 16 | 52112312 | rs3743794 | A | G | 449734 | 0.00276271 | 0.00000036 | 0.986025266 |
| 16 | 52684916 | rs12927162 | A | G | 449734 | 0.00300067 | 6.8E-22 | 1.029064153 |
| 16 | 53800954 | rs1421085 | T | C | 449734 | 0.00274075 | 1.5E-23 | 0.972814269 |
| 16 | 56367969 | rs2550298 | T | C | 449734 | 0.0027767 | 3.6E-18 | 0.976286784 |
| 16 | 60628436 | rs8044054 | T | C | 449734 | 0.00275723 | 1.5E-12 | 1.019403854 |
| 16 | 68136932 | rs72790386 | T | G | 449734 | 0.00751134 | 0.000017 | 1.031690687 |
| 16 | 72210865 | rs17604349 | A | G | 449734 | 0.00351443 | 1.7E-13 | 0.974586306 |
| 17 | 8064083 | rs1061032 | T | G | 449734 | 0.00477391 | 2E-16 | 1.039528973 |
| 17 | 17398278 | rs11545787 | A | G | 449734 | 0.0031377 | 4.9E-14 | 0.976471417 |
| 17 | 30603994 | rs12950382 | A | G | 449734 | 0.0030171 | 0.000038 | 1.01269774 |
| 17 | 31625887 | rs4365329 | A | T | 449734 | 0.00270572 | 0.000032 | 0.989228038 |
| 17 | 33980566 | rs2011528 | T | C | 449734 | 0.00359054 | 5.3E-09 | 0.979524431 |
| 17 | 43047083 | rs3760381 | A | G | 449734 | 0.00311595 | 0.00000093 | 1.015148696 |
| 17 | 44189067 | rs7225002 | A | G | 449734 | 0.00273339 | 0.0000043 | 0.987281769 |
| 17 | 45054564 | rs12600452 | A | G | 449734 | 0.00332908 | 0.000009 | 1.014700606 |
| 17 | 46103760 | rs12051 | A | G | 449734 | 0.0027634 | 0.00000005 | 0.985532672 |
| 17 | 50092201 | rs55846845 | A | G | 449734 | 0.00269305 | 2.4E-09 | 0.98412732 |
| 17 | 54173733 | rs72829706 | A | G | 449734 | 0.00693598 | 5.1E-09 | 1.041551262 |
| 17 | 55734198 | rs8072058 | A | T | 449734 | 0.0032573 | 0.000019 | 0.985908429 |
| 17 | 56709058 | rs412000 | C | G | 449734 | 0.00270849 | 0.0000019 | 0.987106345 |
| 17 | 57934654 | rs58681483 | A | G | 449734 | 0.00503574 | 0.00037 | 1.018193216 |
| 17 | 61391114 | rs72841368 | A | T | 449734 | 0.00344327 | 8.7E-09 | 0.980434734 |
| 17 | 65482109 | rs2916148 | A | G | 449734 | 0.0027342 | 3.5E-09 | 1.016347075 |
| 18 | 1816036 | rs2580160 | A | G | 449734 | 0.00273342 | 0.000000017 | 1.015468315 |
| 18 | 5186566 | rs62082402 | T | G | 449734 | 0.00342345 | 1.6E-14 | 1.025947012 |
| 18 | 21159630 | rs1788784 | A | G | 449734 | 0.00283533 | 0.0017 | 0.991824156 |
| 18 | 22630836 | rs1013987 | T | C | 449734 | 0.00274466 | 1.5E-11 | 0.981899223 |
| 18 | 31663654 | rs4419127 | A | G | 449734 | 0.00285599 | 1.3E-14 | 1.022045284 |
| 18 | 35762461 | rs9950528 | A | G | 449734 | 0.0028552 | 0.0000031 | 0.986814797 |
| 18 | 38152835 | rs12969848 | T | C | 449734 | 0.00270301 | 1.3E-15 | 1.022103951 |
| 18 | 44773382 | rs9956387 | A | T | 449734 | 0.00269715 | 0.0000005 | 0.986804731 |
| 18 | 53429655 | rs4800998 | A | T | 449734 | 0.00346394 | 0.0000078 | 1.0156306 |
| 18 | 56824041 | rs9964420 | A | C | 449734 | 0.00293643 | 8.6E-22 | 0.971921635 |
| 18 | 60240352 | rs11152350 | A | C | 449734 | 0.00269517 | 1.3E-09 | 0.983828486 |
| 18 | 64526233 | rs34329963 | T | C | 449734 | 0.00423824 | 0.000012 | 0.981969726 |
| 18 | 73056278 | rs1025601 | T | C | 449734 | 0.0028031 | 0.00000013 | 0.985041408 |
| 19 | 2695661 | rs10402849 | T | C | 449734 | 0.00335946 | 8.9E-09 | 1.019447588 |
| 19 | 5799433 | rs36055559 | A | G | 449734 | 0.00372199 | 0.000000015 | 0.979129664 |
| 19 | 10770305 | rs7248205 | T | C | 449734 | 0.00275446 | 0.000000038 | 1.015011051 |
| 19 | 18449238 | rs9636202 | A | G | 449734 | 0.00305534 | 7.5E-10 | 0.981794362 |
| 19 | 31052954 | rs73026775 | A | G | 449734 | 0.00416989 | 0.000012 | 0.982131666 |
| 19 | 31673388 | rs4804951 | A | G | 449734 | 0.0028711 | 0.00000018 | 1.015441101 |
| 19 | 41353107 | rs56113850 | T | C | 449734 | 0.00272306 | 0.0000063 | 0.987995437 |
| 19 | 42600984 | rs58876439 | A | G | 449734 | 0.00530315 | 0.00000007 | 1.029198661 |
| 19 | 47003906 | rs11670534 | T | C | 449734 | 0.00362802 | 8.5E-09 | 0.979242956 |
| 20 | 16222093 | rs6131805 | T | G | 449734 | 0.00279935 | 0.000000015 | 1.015757765 |
| 20 | 17348608 | rs6131942 | A | G | 449734 | 0.00273235 | 9.4E-11 | 0.982609295 |
| 20 | 20077178 | rs1474754 | A | G | 449734 | 0.00304293 | 0.000026 | 0.987145139 |
| 20 | 21539564 | rs6047481 | A | T | 449734 | 0.00287782 | 0.0000014 | 1.014060634 |
| 20 | 31051699 | rs1737893 | T | C | 449734 | 0.00278957 | 0.00000035 | 0.98592588 |
| 20 | 43538733 | rs2072727 | T | C | 449734 | 0.00271514 | 8.5E-10 | 1.016486426 |
| 20 | 44668401 | rs57236847 | C | G | 449734 | 0.00275467 | 0.000053 | 1.011376223 |
| 22 | 28848278 | rs695459 | T | C | 449734 | 0.00276149 | 0.00000032 | 0.98602142 |
| 22 | 35846168 | rs28459838 | T | C | 449734 | 0.00319138 | 0.0000019 | 1.015258338 |
| 22 | 38851675 | rs118047999 | C | G | 449734 | 0.00311793 | 0.00035 | 1.010900886 |
| 22 | 40704052 | rs139911 | T | C | 449734 | 0.00273231 | 2.3E-17 | 0.97687996 |
| 22 | 41864190 | rs9611597 | A | T | 449734 | 0.00369486 | 0.000000018 | 1.021092467 |
| 22 | 45728370 | rs6007594 | A | G | 449734 | 0.00306305 | 0.00000016 | 0.983999982 |
| X | 68751721 | rs3747463 | T | C | - | - | - | - |
| X | 82704455 | rs213462 | A | C | - | - | - | - |
| X | 101012848 | rs7060620 | A | T | - | - | - | - |

^a^Chr, chromosome; BP, basepair position; SNP, single nucleotide polymorphism; A1, effect allele; A2, non-effect allele; N, number of participants; SE, standard error; P, two-sided P-value; OR, odd ratio for the association of SNP with outcome.

**Supplementary table 3.** Genome-wide association signals for sleep duration in participants of European ancestry from the UK Biobank (n = 452,633)^a^

| CHR | BP | SNP | A1 | A2 | N | SE | P | OR |
| --- | --- | --- | --- | --- | --- | --- | --- | --- |
| 1 | 31492150 | rs4642942 | C | G | - | 0.004660153 | 0.00019 | 1.017464292 |
| 1 | 98520219 | rs2660302 | T | A | - | 0.005906188 | 5E-10 | 0.964290721 |
| 2 | 58047263 | rs2717052 | C | A | - | 0.004747529 | 0.00043 | 0.983543201 |
| 2 | 66750564 | rs113851554 | G | T | - | 0.010292586 | 3.1E-18 | 1.093803543 |
| 2 | 113348562 | rs62158211 | G | T | - | 0.005603372 | 5.9E-17 | 0.954241307 |
| 2 | 114085785 | rs7556815 | G | A | - | 0.005545211 | 1.5E-18 | 0.952510619 |
| 3 | 70470834 | rs17732997 | C | G | - | 0.004640249 | 0.00013 | 1.018112947 |
| 3 | 137031237 | rs7616632 | T | G | - | 0.004611169 | 0.0000022 | 1.022031133 |
| 6 | 19102286 | rs72828540 | T | C | - | 0.005333812 | 3E-12 | 1.037838745 |
| 6 | 27034181 | rs7764984 | A | G | - | 0.004902433 | 0.00028 | 1.017901759 |
| 6 | 38437303 | rs9369062 | A | C | - | 0.005068544 | 0.0000011 | 0.975621799 |
| 8 | 41723550 | rs13282541 | C | T | - | 0.005241571 | 0.0000018 | 1.025429051 |
| 8 | 105987057 | rs2880370 | A | T | - | 0.004924368 | 0.00002 | 1.021217552 |
| 9 | 37100525 | rs10973207 | G | T | - | 0.006359023 | 0.000043 | 0.974531843 |
| 11 | 122830251 | rs1057703 | T | G | - | 0.00654659 | 0.000026 | 0.972964706 |
| 14 | 63353479 | rs10138240 | G | C | - | 0.004620326 | 0.0000091 | 1.020569422 |
| 16 | 23909538 | rs11643715 | C | G | - | 0.005046916 | 0.0000061 | 0.977360964 |
| 16 | 53800629 | rs9940646 | C | G | - | 0.004650096 | 0.000031 | 1.019489402 |
| 17 | 44083402 | rs1991556 | G | A | - | 0.005545364 | 3.4E-09 | 1.033348799 |
| 19 | 9942123 | rs34786000 | G | T | - | 0.004669157 | 2.5E-09 | 0.972510575 |
| 1 | 34731984 | rs915416 | C | G | - | 0.005060977 | 0.012 | 1.012958395 |
| 1 | 57864304 | rs269054 | T | A | - | 0.0046509 | 0.097 | 0.992336337 |
| 1 | 66476437 | rs61796569 | C | T | - | 0.005205498 | 0.19 | 0.993402834 |
| 1 | 71703970 | rs6681755 | G | A | - | 0.005766284 | 0.57 | 1.003417531 |
| 1 | 79182694 | rs12731287 | G | A | - | 0.005423716 | 0.13 | 1.008467139 |
| 2 | 9185564 | rs62120041 | T | C | - | 0.009296437 | 0.049 | 1.018511572 |
| 2 | 40382712 | rs374153 | C | T | - | 0.006291322 | 0.008 | 1.016876185 |
| 2 | 58871658 | rs75539574 | A | C | - | 0.008248372 | 0.0033 | 0.97605588 |
| 2 | 139195328 | rs12611523 | A | G | - | 0.004617835 | 0.1 | 1.007565583 |
| 2 | 144496866 | rs80071665 | G | T | - | 0.00717182 | 0.026 | 0.983839571 |
| 2 | 147612734 | rs4128364 | T | C | - | 0.004874962 | 0.03 | 0.989624488 |
| 2 | 157049217 | rs11883686 | A | T | - | 0.004872682 | 0.072 | 1.00862615 |
| 2 | 166942663 | rs12463754 | C | G | - | 0.005177261 | 0.78 | 0.998463823 |
| 2 | 178565913 | rs17400325 | T | C | - | 0.011426628 | 0.58 | 1.006507905 |
| 2 | 210377845 | rs10173260 | T | C | - | 0.004706207 | 0.96 | 1.000087544 |
| 2 | 225475560 | rs56337305 | T | C | - | 0.004742452 | 0.052 | 0.990668524 |
| 3 | 55879269 | rs112230981 | A | G | - | 0.010747663 | 0.07 | 1.019147384 |
| 3 | 74961196 | rs9810474 | C | T | - | 0.00546046 | 0.16 | 0.992501169 |
| 3 | 107564459 | rs7644809 | T | C | - | 0.00466954 | 0.36 | 1.004274474 |
| 3 | 118195601 | rs4688116 | G | T | - | 0.004690862 | 0.26 | 0.994672365 |
| 3 | 123283309 | rs16834426 | G | A | - | 0.005212548 | 0.41 | 0.995535684 |
| 3 | 135838598 | rs13088093 | T | G | - | 0.00488341 | 0.00072 | 0.98361908 |
| 4 | 18327896 | rs2192528 | A | G | - | 0.004605709 | 0.031 | 1.009771494 |
| 4 | 44535866 | rs7686205 | A | G | - | 0.004737069 | 0.16 | 0.993469425 |
| 4 | 82272105 | rs41501452 | A | G | - | 0.004948218 | 0.3 | 1.005011308 |
| 4 | 92533225 | rs35531607 | T | C | - | 0.004619732 | 0.1 | 0.992550511 |
| 4 | 102896591 | rs13109404 | T | G | - | 0.009001648 | 0.2 | 1.011349671 |
| 5 | 1428883 | rs365663 | A | G | - | 0.004637682 | 0.58 | 1.00266744 |
| 5 | 3126584 | rs460692 | C | T | - | 0.006790479 | 0.13 | 1.010306756 |
| 5 | 102321905 | rs56372231 | C | T | - | 0.004868352 | 0.0015 | 0.984573502 |
| 5 | 135615615 | rs180769 | T | C | - | 0.004665307 | 0.08 | 1.008163814 |
| 5 | 137769576 | rs113322698 | G | A | - | 0.004838008 | 0.0014 | 0.984814432 |
| 5 | 176751059 | rs151014368 | G | A | - | 0.005723238 | 0.4 | 0.99512655 |
| 6 | 28584775 | rs34556183 | A | G | - | 0.005126513 | 0.017 | 1.012543807 |
| 6 | 29764472 | rs1633005 | C | T | - | 0.005689579 | 0.78 | 1.001571412 |
| 6 | 30799168 | rs4587207 | A | G | - | 0.006566456 | 0.14 | 1.009756463 |
| 6 | 33464363 | rs80193650 | A | G | - | 0.00622864 | 0.07 | 0.988666382 |
| 6 | 43160375 | rs113113059 | T | C | - | 0.005573793 | 0.19 | 0.992909624 |
| 6 | 54937974 | rs9382445 | T | C | - | 0.004752433 | 0.2 | 1.006203897 |
| 6 | 89798679 | rs9451146 | T | C | - | 0.005491264 | 0.46 | 0.995915224 |
| 6 | 93157567 | rs9362971 | C | T | - | 0.00484364 | 0.25 | 0.994595081 |
| 6 | 130640112 | rs4897409 | G | A | - | 0.004978123 | 0.015 | 1.012268285 |
| 7 | 2106928 | rs34731055 | C | T | - | 0.005946264 | 0.24 | 0.993054653 |
| 7 | 107197694 | rs6979198 | T | G | - | 0.005624234 | 0.43 | 1.004284824 |
| 7 | 114126432 | rs2079070 | C | G | - | 0.005193257 | 0.91 | 0.999532263 |
| 7 | 132568255 | rs11982852 | C | T | - | 0.005367107 | 0.88 | 1.000895955 |
| 8 | 9149746 | rs330088 | T | C | - | 0.004619502 | 0.0027 | 0.986417587 |
| 8 | 10190040 | rs7832708 | C | T | - | 0.004602567 | 0.025 | 0.989809785 |
| 8 | 10716028 | rs2292369 | A | G | - | 0.004703065 | 0.0055 | 1.013109748 |
| 8 | 11450422 | rs2244648 | A | G | - | 0.0046209 | 0.0072 | 0.98775717 |
| 8 | 14279446 | rs73219758 | G | A | - | 0.005068084 | 0.16 | 1.007165473 |
| 8 | 73890425 | rs4588900 | G | A | - | 0.004603927 | 0.92 | 1.000356496 |
| 9 | 4120648 | rs1570203 | G | A | - | 0.004638946 | 0.2 | 0.994169478 |
| 10 | 21830580 | rs12246842 | A | G | - | 0.004633257 | 0.63 | 1.002371659 |
| 10 | 64618340 | rs10761674 | C | T | - | 0.004602203 | 0.61 | 1.002292797 |
| 10 | 103128332 | rs11190970 | G | A | - | 0.005716322 | 0.019 | 1.013553964 |
| 10 | 104078044 | rs144625846 | A | G | - | 0.006746667 | 0.14 | 1.010179876 |
| 10 | 125016501 | rs7915425 | T | C | - | 0.00603728 | 0.063 | 1.011397121 |
| 11 | 28829882 | rs1517572 | A | C | - | 0.004673946 | 0.016 | 0.988735496 |
| 11 | 43800474 | rs4592416 | A | G | - | 0.004611169 | 0.0037 | 0.986696714 |
| 11 | 48162453 | rs11602180 | C | T | - | 0.006187337 | 0.93 | 1.000368583 |
| 11 | 61581764 | rs174560 | T | C | - | 0.004937146 | 0.37 | 0.995625725 |
| 11 | 80685181 | rs12791153 | A | T | - | 0.008575038 | 0.44 | 0.993843188 |
| 11 | 88297740 | rs1553132 | A | G | - | 0.005263525 | 0.12 | 0.99187001 |
| 11 | 101520886 | rs1939455 | G | T | - | 0.007254674 | 0.62 | 0.996675806 |
| 11 | 113408518 | rs7115226 | C | A | - | 0.008872088 | 0.0044 | 0.975326728 |
| 11 | 116576415 | rs1263056 | A | G | - | 0.004625402 | 0.82 | 0.999048078 |
| 11 | 118358027 | rs7951019 | T | G | - | 0.013104847 | 0.13 | 1.01990783 |
| 12 | 32851866 | rs188906236 | G | A | - | 0.023973946 | 0.69 | 1.010015145 |
| 12 | 38764559 | rs34354917 | C | A | - | 0.005071743 | 0.94 | 0.999631982 |
| 12 | 67645219 | rs800165 | C | T | - | 0.00486908 | 0.0075 | 1.013111786 |
| 12 | 110007939 | rs11614986 | A | G | - | 0.005965192 | 0.017 | 1.014202108 |
| 12 | 117951150 | rs4767550 | A | G | - | 0.004694253 | 0.0092 | 0.987925614 |
| 14 | 26954078 | rs6575005 | T | C | - | 0.005348544 | 0.17 | 1.007487992 |
| 14 | 29816155 | rs10483350 | A | G | - | 0.005822625 | 0.48 | 1.003854325 |
| 14 | 60233841 | rs61985058 | C | T | - | 0.006542414 | 0.27 | 0.992815759 |
| 14 | 65554638 | rs55658675 | C | T | - | 0.004825402 | 0.061 | 1.009022625 |
| 14 | 78495761 | rs11621908 | C | T | - | 0.008430077 | 0.029 | 1.018674898 |
| 14 | 93752603 | rs61980273 | G | A | - | 0.012847222 | 0.12 | 0.979942954 |
| 15 | 48050577 | rs13329140 | G | A | - | 0.00542113 | 0.58 | 0.9970885 |
| 16 | 6550400 | rs3095508 | C | A | - | 0.004673716 | 0.0018 | 1.014861612 |
| 16 | 19987636 | rs28651105 | A | G | - | 0.005579866 | 0.1 | 0.991086894 |
| 16 | 56120461 | rs8050478 | G | A | - | 0.004594483 | 0.31 | 1.004737624 |
| 16 | 61520904 | rs17822558 | G | A | - | 0.004935402 | 0.31 | 1.005046349 |
| 17 | 4732467 | rs11654671 | A | T | - | 0.005421149 | 0.87 | 1.000787254 |
| 17 | 8134275 | rs7503199 | C | T | - | 0.005191916 | 0.37 | 0.995443115 |
| 17 | 11227352 | rs205024 | C | T | - | 0.004724368 | 0.079 | 0.991730816 |
| 17 | 21313223 | rs2139261 | C | G | - | 0.005894808 | 0.039 | 0.987845713 |
| 17 | 45539117 | rs9895274 | C | T | - | 0.004608697 | 0.33 | 1.004686503 |
| 17 | 50571227 | rs9903973 | C | T | - | 0.00462387 | 0.24 | 1.005370693 |
| 18 | 53059748 | rs12607679 | T | C | - | 0.005269483 | 0.73 | 0.998314742 |
| 20 | 43538733 | rs2072727 | T | C | - | 0.004645556 | 0.67 | 0.998196047 |

^a^Chr, chromosome; BP, basepair position; SNP, single nucleotide polymorphism; A1, effect allele; A2, non-effect allele; N, number of participants; SE, standard error; P, two-sided P-value; OR, odd ratio for the association of SNP with outcome.

**Supplementary table 4.** Genome-wide association signals for insomnia in participants of European ancestry from the UK Biobank (n = 452,633)^a^

| CHR | BP | SNP | A1 | A2 | N | SE | P | OR |
| --- | --- | --- | --- | --- | --- | --- | --- | --- |
| 16 | 51177517 | rs1015438 | A | G | - | 0.008 | 2.51E-14 | 1.081122659 |
| 13 | 54382035 | rs1031654 | A | C | - | 0.007 | 3.88E-12 | 0.95 |
| 15 | 74012409 | rs1038093 | T | C | - | 0.006 | 2.47E-10 | 1.04 |
| 18 | 50748499 | rs10502966 | A | G | - | 0.006 | 8.54E-11 | 0.962 |
| 2 | 198950240 | rs1064213 | A | G | - | 0.006 | 6.41E-10 | 0.964 |
| 11 | 118396331 | rs1064939 | A | T | - | 0.02 | 2.16E-10 | 1.138999999 |
| 9 | 14534505 | rs10756571 | T | C | - | 0.006 | 0.000000018 | 1.037 |
| 9 | 4292083 | rs10758593 | A | G | - | 0.006 | 4.9E-09 | 0.965 |
| 9 | 96361922 | rs10761240 | A | G | - | 0.006 | 2.12E-12 | 0.958 |
| 1 | 190900576 | rs10800992 | T | C | - | 0.006 | 3.84E-12 | 1.043 |
| 10 | 57177470 | rs10825503 | T | G | - | 0.006 | 1.43E-08 | 1.034 |
| 3 | 49211989 | rs10865954 | T | C | - | 0.006 | 1.92E-11 | 1.043 |
| 11 | 73455292 | rs10898940 | A | C | - | 0.006 | 8.09E-09 | 1.035 |
| 2 | 146458738 | rs10928256 | T | C | - | 0.006 | 1.61E-08 | 1.035 |
| 6 | 94498850 | rs10944696 | A | G | - | 0.007 | 7.99E-09 | 0.963 |
| 6 | 33647058 | rs10947428 | T | C | - | 0.007 | 9.06E-21 | 0.934 |
| 6 | 37631768 | rs10947690 | A | G | - | 0.007 | 4.04E-12 | 0.954 |
| 6 | 41754370 | rs10947987 | T | C | - | 0.006 | 4.08E-08 | 0.968 |
| 8 | 114154187 | rs10955647 | T | G | - | 0.006 | 1.84E-08 | 1.034 |
| 10 | 76825638 | rs11001276 | A | T | - | 0.007 | 2.52E-08 | 0.963 |
| 22 | 41496800 | rs11090039 | A | G | - | 0.007 | 1.82E-09 | 1.04 |
| 1 | 210293333 | rs11119409 | T | C | - | 0.006 | 1.19E-08 | 0.966 |
| 2 | 66789341 | rs11126082 | C | G | - | 0.006 | 8.26E-13 | 0.958 |
| 13 | 85294881 | rs11149313 | A | G | - | 0.007 | 2.38E-09 | 1.041 |
| 2 | 66750564 | rs113851554 | T | G | - | 0.014 | 1.56E-51 | 1.229000001 |
| 6 | 147980909 | rs1147852 | A | G | - | 0.006 | 9.94E-10 | 1.04 |
| 1 | 57819204 | rs11588755 | A | G | - | 0.006 | 5.14E-09 | 0.966 |
| 11 | 47606483 | rs11605348 | A | G | - | 0.006 | 7.01E-13 | 0.956 |
| 2 | 159137557 | rs116466468 | T | C | - | 0.007 | 2.11E-10 | 1.045 |
| 17 | 46035001 | rs11650304 | C | G | - | 0.012 | 1.23E-08 | 1.069 |
| 12 | 43484487 | rs1167132 | T | C | - | 0.006 | 8.73E-09 | 1.036 |
| 2 | 77724624 | rs11679943 | A | G | - | 0.006 | 3.16E-09 | 1.038 |
| 4 | 112822731 | rs11722569 | T | C | - | 0.006 | 2.91E-08 | 1.035 |
| 6 | 18843810 | rs11756035 | C | G | - | 0.009 | 1.29E-08 | 1.052 |
| 13 | 54018867 | rs117630493 | C | G | - | 0.018 | 3.61E-08 | 0.904 |
| 1 | 190060095 | rs11803128 | A | G | - | 0.006 | 6.85E-11 | 0.96 |
| 9 | 8858043 | rs118166957 | T | C | - | 0.008 | 1.95E-16 | 1.069999999 |
| 13 | 60362013 | rs11838830 | A | G | - | 0.013 | 5.2E-10 | 0.923 |
| 1 | 96961268 | rs12030482 | A | T | - | 0.007 | 8.16E-09 | 1.042 |
| 5 | 102660400 | rs12187443 | T | C | - | 0.006 | 1.64E-10 | 1.041 |
| 10 | 21821918 | rs12251016 | A | T | - | 0.006 | 3.89E-10 | 0.962 |
| 12 | 84700945 | rs12310246 | A | G | - | 0.007 | 4.74E-11 | 1.046 |
| 18 | 26315799 | rs12454003 | C | G | - | 0.006 | 4.94E-09 | 0.966 |
| 5 | 61514611 | rs12520974 | T | C | - | 0.006 | 1.69E-09 | 0.965 |
| 7 | 3824141 | rs12540241 | A | T | - | 0.008 | 1.58E-09 | 0.955 |
| 18 | 31313965 | rs12605642 | T | G | - | 0.006 | 2.13E-09 | 1.036 |
| 2 | 66792109 | rs12614369 | A | G | - | 0.008 | 7.21E-09 | 1.045 |
| 6 | 30576781 | rs1264419 | C | G | - | 0.006 | 8.91E-10 | 1.037 |
| 7 | 115082406 | rs12666306 | A | G | - | 0.006 | 2.24E-12 | 1.043 |
| 11 | 57667222 | rs12790660 | T | C | - | 0.006 | 4.49E-10 | 0.961 |
| 1 | 117944435 | rs1289939 | T | C | - | 0.007 | 0.000000006 | 0.96 |
| 15 | 38897857 | rs12912299 | T | C | - | 0.006 | 4.42E-13 | 0.958 |
| 15 | 74331659 | rs12917449 | A | C | - | 0.008 | 2.97E-08 | 0.959 |
| 16 | 9191790 | rs12924275 | T | C | - | 0.007 | 1.93E-08 | 1.039 |
| 19 | 5073447 | rs12983032 | A | G | - | 0.006 | 1.07E-11 | 0.958 |
| 2 | 68071990 | rs12991815 | C | G | - | 0.006 | 3.02E-11 | 1.041 |
| 2 | 51824512 | rs13010288 | T | G | - | 0.009 | 9.26E-12 | 0.942 |
| 4 | 103198082 | rs13135092 | A | G | - | 0.011 | 2.53E-16 | 0.915 |
| 4 | 148987430 | rs13138995 | A | G | - | 0.006 | 1.97E-08 | 1.035 |
| 7 | 109200331 | rs1357685 | T | C | - | 0.006 | 1.39E-08 | 1.034 |
| 3 | 50070843 | rs138014720 | A | T | - | 0.013 | 3.46E-08 | 1.072 |
| 6 | 30932223 | rs138678612 | A | G | - | 0.02 | 1.41E-08 | 0.89 |
| 2 | 66677816 | rs1519102 | C | G | - | 0.006 | 0.000000019 | 0.964 |
| 5 | 106849674 | rs152555 | A | G | - | 0.008 | 4.83E-10 | 0.949 |
| 2 | 236900633 | rs1530938 | A | G | - | 0.006 | 8.82E-10 | 1.037 |
| 13 | 111982291 | rs1536053 | T | C | - | 0.006 | 6.04E-09 | 0.963 |
| 17 | 46563707 | rs1553754 | T | G | - | 0.006 | 3.51E-08 | 0.967 |
| 3 | 71435955 | rs1567084 | A | G | - | 0.006 | 2.14E-08 | 1.034 |
| 3 | 107955515 | rs1580173 | A | G | - | 0.006 | 2.28E-08 | 1.034 |
| 1 | 72729142 | rs1620977 | A | G | - | 0.007 | 2.27E-14 | 1.053 |
| 5 | 87693561 | rs16903122 | T | C | - | 0.007 | 9.04E-16 | 1.057 |
| 4 | 34720226 | rs16990210 | T | C | - | 0.008 | 1.97E-08 | 0.955 |
| 4 | 82288564 | rs17005118 | A | G | - | 0.007 | 6.13E-10 | 1.043 |
| 3 | 88001713 | rs17025198 | A | G | - | 0.007 | 2.19E-08 | 1.042 |
| 5 | 92995477 | rs17083297 | A | C | - | 0.008 | 0.000000016 | 0.957 |
| 5 | 50492629 | rs17223714 | A | G | - | 0.007 | 2.44E-10 | 1.047 |
| 7 | 137075847 | rs1731951 | A | T | - | 0.006 | 1.36E-08 | 0.966 |
| 23 | 133134421 | rs17324524 | T | C | - | 0.009 | 5.01E-10 | 0.944 |
| 5 | 107112116 | rs17367725 | T | C | - | 0.006 | 9.29E-09 | 0.965 |
| 7 | 119674508 | rs17520265 | A | G | - | 0.016 | 2.87E-08 | 0.913 |
| 8 | 91650818 | rs17643634 | T | C | - | 0.008 | 1.34E-13 | 0.942 |
| 15 | 89913632 | rs176644 | T | G | - | 0.006 | 9.49E-09 | 1.036 |
| 2 | 58893065 | rs1861412 | A | G | - | 0.006 | 1.67E-10 | 1.039 |
| 7 | 10985188 | rs190073 | A | G | - | 0.006 | 2.86E-08 | 0.967 |
| 9 | 120518991 | rs1927902 | T | C | - | 0.007 | 1.15E-14 | 1.054 |
| 1 | 66358242 | rs1937447 | C | G | - | 0.007 | 2.08E-08 | 0.962 |
| 7 | 21687925 | rs2030672 | C | G | - | 0.006 | 0.000000011 | 1.035 |
| 1 | 37194103 | rs2089358 | T | C | - | 0.007 | 2.75E-10 | 0.96 |
| 11 | 17193475 | rs214934 | A | T | - | 0.006 | 3.16E-09 | 0.963 |
| 3 | 180785697 | rs2216427 | C | G | - | 0.006 | 0.000000016 | 1.036 |
| 11 | 88598444 | rs2221119 | C | G | - | 0.006 | 0.000000002 | 1.037 |
| 10 | 64519299 | rs224029 | T | C | - | 0.006 | 2.51E-10 | 0.962 |
| 12 | 6873818 | rs2286729 | A | G | - | 0.011 | 5.37E-11 | 1.072 |
| 3 | 158522463 | rs2364921 | T | C | - | 0.006 | 2.13E-08 | 0.967 |
| 6 | 29355113 | rs238869 | T | C | - | 0.006 | 3.36E-08 | 0.967 |
| 6 | 99598756 | rs2388840 | A | G | - | 0.006 | 1.37E-09 | 0.964 |
| 13 | 96932868 | rs2389631 | A | C | - | 0.006 | 2.03E-10 | 0.961 |
| 16 | 56352854 | rs2398144 | A | C | - | 0.006 | 5.09E-10 | 1.039 |
| 5 | 103947968 | rs2431108 | T | C | - | 0.006 | 7.83E-17 | 0.948 |
| 17 | 2294048 | rs2447094 | A | C | - | 0.006 | 0.000000025 | 0.967 |
| 13 | 53784083 | rs2491124 | T | C | - | 0.006 | 8.81E-16 | 1.05 |
| 7 | 133989882 | rs2598293 | T | C | - | 0.006 | 2.48E-09 | 1.036 |
| 8 | 116657235 | rs2737240 | A | G | - | 0.007 | 3.37E-08 | 1.037 |
| 9 | 125621610 | rs2792990 | C | G | - | 0.008 | 1.15E-10 | 1.056 |
| 1 | 72764289 | rs2815757 | T | C | - | 0.008 | 2.24E-13 | 1.057 |
| 21 | 46539725 | rs2838787 | A | G | - | 0.006 | 7.65E-09 | 0.965 |
| 8 | 103356226 | rs28552587 | A | G | - | 0.006 | 0.000000033 | 1.034 |
| 12 | 123856998 | rs28582096 | A | G | - | 0.007 | 1.74E-13 | 0.947 |
| 8 | 10170037 | rs28611339 | T | G | - | 0.009 | 8.46E-11 | 1.06 |
| 20 | 41972028 | rs2867690 | T | C | - | 0.008 | 0.000000037 | 1.043 |
| 4 | 106094427 | rs2903385 | A | G | - | 0.006 | 4.53E-13 | 1.044 |
| 6 | 31475127 | rs3131638 | A | G | - | 0.007 | 7.88E-10 | 0.957 |
| 6 | 105400605 | rs314281 | T | C | - | 0.006 | 6.03E-13 | 0.958 |
| 16 | 715164 | rs3184470 | A | G | - | 0.006 | 9.73E-10 | 0.963 |
| 12 | 57487814 | rs324017 | A | C | - | 0.007 | 1.61E-09 | 1.04 |
| 2 | 66815719 | rs34036083 | T | C | - | 0.006 | 2.07E-08 | 0.965 |
| 16 | 52303107 | rs34214423 | A | C | - | 0.008 | 3.18E-09 | 1.046 |
| 17 | 26933741 | rs34490907 | C | G | - | 0.009 | 1.76E-08 | 1.055 |
| 2 | 215382654 | rs34967082 | A | G | - | 0.006 | 4.34E-09 | 1.036 |
| 3 | 43066558 | rs35110063 | A | G | - | 0.006 | 8.82E-11 | 1.04 |
| 16 | 77137324 | rs35322724 | A | C | - | 0.006 | 3.75E-16 | 1.05 |
| 5 | 91607148 | rs35539975 | A | G | - | 0.007 | 4.49E-09 | 1.043 |
| 5 | 106899684 | rs37445 | A | G | - | 0.006 | 4.88E-09 | 0.965 |
| 3 | 50209053 | rs3774751 | T | G | - | 0.006 | 7.32E-12 | 0.96 |
| 16 | 61647589 | rs3902952 | T | C | - | 0.008 | 2.55E-10 | 1.049 |
| 9 | 77118987 | rs4090240 | T | C | - | 0.007 | 8.46E-09 | 0.962 |
| 16 | 52746089 | rs4238755 | A | C | - | 0.007 | 2.3E-10 | 0.958 |
| 3 | 178469932 | rs4260410 | T | C | - | 0.006 | 4.87E-08 | 1.035 |
| 19 | 45411941 | rs429358 | T | C | - | 0.008 | 2.13E-08 | 1.047 |
| 5 | 153093998 | rs4502882 | T | C | - | 0.006 | 7.96E-10 | 0.962 |
| 16 | 52637892 | rs45453598 | A | T | - | 0.008 | 4.42E-09 | 1.048 |
| 8 | 73890425 | rs4588900 | A | G | - | 0.006 | 1.57E-08 | 1.034 |
| 11 | 62697813 | rs4592425 | T | G | - | 0.006 | 4.31E-10 | 1.041 |
| 17 | 47123423 | rs4643373 | T | C | - | 0.007 | 1.58E-10 | 1.042 |
| 2 | 160570033 | rs4664299 | T | C | - | 0.007 | 4.95E-09 | 0.96 |
| 4 | 106055212 | rs4699157 | T | C | - | 0.015 | 3.98E-08 | 0.922 |
| 15 | 91426560 | rs4702 | A | G | - | 0.006 | 6.78E-16 | 0.953 |
| 6 | 163280204 | rs4709655 | T | C | - | 0.009 | 3.09E-09 | 0.947 |
| 12 | 118385788 | rs4767645 | T | G | - | 0.006 | 6.47E-10 | 0.964 |
| 16 | 29978827 | rs4788203 | A | G | - | 0.006 | 6.32E-09 | 0.966 |
| 17 | 2243628 | rs4790076 | T | C | - | 0.008 | 1.76E-09 | 1.049 |
| 3 | 25154112 | rs4858708 | A | T | - | 0.006 | 1.23E-08 | 0.967 |
| 3 | 155432229 | rs492858 | T | C | - | 0.011 | 3.46E-09 | 0.936 |
| 14 | 33412996 | rs4981170 | A | G | - | 0.008 | 7.33E-13 | 0.947 |
| 7 | 49894349 | rs521484 | A | G | - | 0.007 | 1.53E-08 | 0.961 |
| 11 | 66041079 | rs524859 | A | G | - | 0.006 | 1.48E-12 | 0.957 |
| 2 | 208042581 | rs55772859 | A | C | - | 0.006 | 4.82E-11 | 1.043 |
| 5 | 135653737 | rs55972276 | A | C | - | 0.009 | 4.19E-17 | 1.076 |
| 2 | 44262449 | rs56097173 | T | C | - | 0.006 | 2.69E-10 | 1.041 |
| 11 | 72348039 | rs56133505 | A | G | - | 0.006 | 5.59E-12 | 1.042 |
| 11 | 66401373 | rs566673 | T | G | - | 0.006 | 1.18E-10 | 0.962 |
| 1 | 173878862 | rs5877 | T | C | - | 0.006 | 1.23E-08 | 1.037 |
| 20 | 47774512 | rs6019663 | T | C | - | 0.007 | 6.47E-10 | 1.041 |
| 18 | 52906830 | rs60565673 | T | G | - | 0.006 | 1.59E-12 | 0.958 |
| 20 | 31163914 | rs6119267 | C | G | - | 0.006 | 2.32E-20 | 0.942 |
| 1 | 73957815 | rs61765555 | T | C | - | 0.007 | 4E-11 | 0.956 |
| 12 | 66367726 | rs61921611 | T | C | - | 0.006 | 7.84E-12 | 0.957 |
| 17 | 2400876 | rs62068188 | T | C | - | 0.008 | 1.18E-09 | 1.05 |
| 2 | 73853830 | rs62149809 | A | G | - | 0.025 | 5.71E-09 | 1.159 |
| 2 | 114082175 | rs62158170 | A | G | - | 0.007 | 1.2E-19 | 1.068 |
| 2 | 239222376 | rs62194948 | C | G | - | 0.007 | 4.64E-09 | 1.04 |
| 2 | 210380152 | rs62213452 | T | G | - | 0.007 | 2.39E-08 | 1.038 |
| 3 | 117642005 | rs62264767 | A | C | - | 0.008 | 1.63E-14 | 1.067 |
| 4 | 22050165 | rs62301574 | C | G | - | 0.007 | 1.37E-08 | 0.959 |
| 1 | 201765094 | rs623025 | T | C | - | 0.007 | 3.16E-08 | 0.963 |
| 5 | 165460085 | rs62383308 | A | G | - | 0.011 | 3.98E-08 | 0.942 |
| 6 | 140324582 | rs62429521 | A | C | - | 0.008 | 1.78E-09 | 1.052 |
| 23 | 21494390 | rs62590551 | A | G | - | 0.01 | 4.61E-09 | 1.056 |
| 6 | 34828553 | rs6457796 | T | C | - | 0.007 | 1.12E-08 | 0.962 |
| 7 | 88310899 | rs6465151 | T | C | - | 0.009 | 1.9E-09 | 1.058 |
| 11 | 121534938 | rs647905 | T | C | - | 0.006 | 2.87E-08 | 1.034 |
| 19 | 30710785 | rs6510033 | A | G | - | 0.007 | 4.66E-08 | 0.964 |
| 2 | 60521311 | rs6545798 | A | T | - | 0.006 | 1.19E-11 | 0.96 |
| 13 | 60532796 | rs6562066 | T | C | - | 0.006 | 1.38E-10 | 1.04 |
| 11 | 99126016 | rs6589988 | A | G | - | 0.006 | 4.7E-09 | 0.963 |
| 9 | 133786652 | rs6597649 | T | C | - | 0.006 | 3.05E-08 | 1.034 |
| 5 | 179511043 | rs6601080 | A | G | - | 0.006 | 2.21E-08 | 1.036 |
| 12 | 109982578 | rs6606731 | A | T | - | 0.008 | 1.51E-08 | 1.044 |
| 16 | 19904344 | rs66674044 | A | T | - | 0.009 | 2.18E-12 | 0.942 |
| 11 | 83277325 | rs667730 | T | C | - | 0.006 | 2.26E-08 | 1.034 |
| 1 | 107190062 | rs6702604 | A | G | - | 0.006 | 1.3E-09 | 0.964 |
| 7 | 108625185 | rs670501 | T | C | - | 0.007 | 7.4E-13 | 1.054 |
| 8 | 60914783 | rs671985 | A | G | - | 0.006 | 2.79E-10 | 0.963 |
| 2 | 42813247 | rs6734957 | T | G | - | 0.007 | 1.82E-09 | 0.959 |
| 16 | 20006745 | rs67501351 | C | G | - | 0.007 | 5.36E-11 | 1.046 |
| 2 | 147480394 | rs6756610 | C | G | - | 0.006 | 1.14E-09 | 1.038 |
| 3 | 10581380 | rs6808140 | T | C | - | 0.006 | 5.35E-11 | 1.04 |
| 5 | 141254063 | rs6888135 | A | C | - | 0.006 | 1.21E-10 | 1.039 |
| 3 | 173112907 | rs694786 | T | C | - | 0.006 | 1.97E-13 | 0.957 |
| 7 | 132672192 | rs6967168 | T | G | - | 0.007 | 1.39E-10 | 0.957 |
| 7 | 102008352 | rs6973090 | A | G | - | 0.007 | 4.31E-08 | 0.963 |
| 7 | 1966841 | rs6978112 | T | C | - | 0.006 | 2.11E-08 | 1.035 |
| 1 | 74878253 | rs699844 | A | G | - | 0.011 | 4.11E-08 | 1.062 |
| 5 | 80296487 | rs701394 | A | G | - | 0.006 | 6.83E-09 | 0.965 |
| 9 | 134886837 | rs7040224 | A | G | - | 0.006 | 4.24E-09 | 1.038 |
| 9 | 81739348 | rs7044885 | C | G | - | 0.006 | 5.67E-12 | 0.96 |
| 15 | 57215867 | rs715338 | A | G | - | 0.006 | 7.85E-12 | 1.042 |
| 5 | 106918329 | rs71575448 | A | G | - | 0.009 | 3.38E-09 | 1.052 |
| 15 | 66709386 | rs7168238 | C | G | - | 0.011 | 0.000000018 | 1.066 |
| 17 | 43157709 | rs7214267 | A | G | - | 0.006 | 5.09E-13 | 0.957 |
| 4 | 90820809 | rs72657797 | T | C | - | 0.008 | 1.52E-12 | 0.946 |
| 9 | 139109080 | rs72773790 | T | C | - | 0.006 | 3.71E-09 | 1.038 |
| 6 | 124292594 | rs728017 | A | G | - | 0.006 | 9.51E-09 | 0.966 |
| 2 | 104412924 | rs72820274 | A | G | - | 0.006 | 1.28E-08 | 1.035 |
| 11 | 45415577 | rs72899452 | T | C | - | 0.012 | 0.000000001 | 1.077 |
| 3 | 49863483 | rs73079014 | T | C | - | 0.009 | 3.65E-08 | 0.952 |
| 3 | 117602144 | rs73163783 | T | C | - | 0.007 | 1.39E-08 | 0.963 |
| 7 | 3520024 | rs73671843 | A | G | - | 0.009 | 5.49E-10 | 0.946 |
| 15 | 99183876 | rs7402939 | T | C | - | 0.006 | 5.19E-09 | 0.965 |
| 20 | 50985290 | rs742760 | A | T | - | 0.008 | 2.48E-08 | 1.044 |
| 3 | 48941551 | rs7432782 | T | C | - | 0.014 | 7.42E-09 | 0.92 |
| 10 | 77771194 | rs7475916 | C | G | - | 0.006 | 6.7E-09 | 0.964 |
| 12 | 84336911 | rs7486418 | T | G | - | 0.006 | 6.84E-11 | 1.042 |
| 2 | 67134426 | rs75452188 | A | G | - | 0.009 | 1.58E-08 | 1.053 |
| 2 | 66972843 | rs7566062 | T | C | - | 0.007 | 1.37E-16 | 1.061 |
| 2 | 176473295 | rs7571486 | A | G | - | 0.007 | 0.000000014 | 0.962 |
| 7 | 106844694 | rs75932578 | T | C | - | 0.007 | 4.15E-08 | 0.961 |
| 2 | 239231477 | rs7599697 | T | C | - | 0.006 | 0.000000005 | 0.964 |
| 20 | 62670427 | rs76145129 | T | G | - | 0.009 | 2.73E-08 | 0.951 |
| 3 | 18718055 | rs7615602 | C | G | - | 0.007 | 2.59E-09 | 0.961 |
| 3 | 44062561 | rs7625896 | A | G | - | 0.006 | 5.28E-09 | 1.037 |
| 9 | 140265782 | rs77641763 | T | C | - | 0.009 | 6.53E-15 | 1.074 |
| 2 | 67022234 | rs78206187 | A | G | - | 0.013 | 2.96E-13 | 0.91 |
| 13 | 53969796 | rs79204944 | A | G | - | 0.014 | 4.24E-08 | 1.082 |
| 11 | 72340686 | rs79693059 | C | G | - | 0.011 | 1.61E-11 | 0.93 |
| 13 | 54721699 | rs7992992 | A | G | - | 0.009 | 1.15E-08 | 1.052 |
| 17 | 61024696 | rs8076183 | T | C | - | 0.006 | 2.75E-10 | 0.963 |
| 5 | 107209814 | rs8180457 | T | C | - | 0.008 | 1.12E-11 | 0.946 |
| 7 | 114047542 | rs8180817 | C | G | - | 0.006 | 1.83E-16 | 0.952 |
| 13 | 53613990 | rs8181889 | A | G | - | 0.006 | 8.9E-10 | 0.963 |
| 2 | 2850540 | rs823247 | T | C | - | 0.006 | 5.25E-10 | 0.964 |
| 16 | 12323509 | rs830716 | C | G | - | 0.007 | 8.68E-12 | 1.046 |
| 8 | 35190619 | rs871994 | A | C | - | 0.006 | 5.5E-09 | 1.036 |
| 8 | 30849450 | rs874168 | T | C | - | 0.006 | 7.95E-09 | 1.035 |
| 19 | 56134038 | rs908668 | T | C | - | 0.007 | 1.41E-11 | 1.051 |
| 20 | 45841052 | rs910187 | A | G | - | 0.006 | 1.63E-08 | 0.966 |
| 13 | 53978628 | rs9316619 | T | C | - | 0.008 | 5.5E-09 | 1.047 |
| 6 | 101212001 | rs9373590 | A | T | - | 0.006 | 2.18E-11 | 1.041 |
| 6 | 38452503 | rs9394502 | T | C | - | 0.006 | 7.76E-18 | 0.947 |
| 7 | 3323848 | rs940780 | T | C | - | 0.006 | 8.5E-10 | 1.039 |
| 6 | 33455574 | rs9469434 | C | G | - | 0.007 | 4.41E-08 | 0.965 |
| 13 | 53991125 | rs9527083 | A | G | - | 0.006 | 1.61E-32 | 0.927 |
| 13 | 66947124 | rs9540729 | A | T | - | 0.006 | 1.4E-09 | 1.037 |
| 13 | 61720066 | rs9563886 | T | C | - | 0.006 | 3.08E-08 | 0.967 |
| 2 | 66817402 | rs984306 | T | C | - | 0.007 | 7.94E-10 | 0.958 |
| 17 | 50259142 | rs9889282 | A | C | - | 0.006 | 4.7E-12 | 0.959 |
| 16 | 56128782 | rs9931543 | T | C | - | 0.007 | 1.11E-12 | 1.049 |
| 18 | 56824041 | rs9964420 | A | C | - | 0.007 | 4.54E-08 | 1.036 |

^a^Chr, chromosome; BP, basepair position; SNP, single nucleotide polymorphism; A1, effect allele; A2, non-effect allele; N, number of participants; SE, standard error; P, two-sided P-value; OR, odd ratio for the association of SNP with outcome.

**Supplementary table 5.** Genome-wide association signals for daytime sleepiness in participants of European ancestry from the UK Biobank (n = 452,633)^a^

| CHR | BP | SNP | A1 | A2 | N | SE | P | OR |
| --- | --- | --- | --- | --- | --- | --- | --- | --- |
| 12 | 117942076 | rs11615756 | T | C | - | 0.00123883 | 1.4E-49 | 1.018435168 |
| 17 | 43686419 | rs385199 | A | C | - | 0.0014497 | 7.5E-47 | 1.021133311 |
| 1 | 62579891 | rs12140153 | G | T | - | 0.0021 | 2.4E-31 | 1.025007572 |
| 6 | 55142337 | rs2653349 | A | G | - | 0.00147827 | 3.4E-29 | 1.016732648 |
| 18 | 44788274 | rs9965170 | G | A | - | 0.00122874 | 7.8E-29 | 1.013730813 |
| 1 | 201860626 | rs2250377 | A | G | - | 0.00127975 | 1.4E-24 | 1.013243022 |
| 5 | 103947968 | rs2431108 | C | T | - | 0.00129028 | 7.7E-24 | 1.013067341 |
| 9 | 81727018 | rs13284688 | C | T | - | 0.00149894 | 1.7E-23 | 1.015185851 |
| 6 | 124920871 | rs614987 | C | A | - | 0.00124925 | 7.5E-19 | 1.011082663 |
| 5 | 146693062 | rs10875622 | A | G | - | 0.0012291 | 1.3E-17 | 1.010517019 |
| 2 | 236801226 | rs796858289 | GA | G | - | 0.0012423 | 5E-17 | 1.010540969 |
| 6 | 37699156 | rs6919087 | T | G | - | 0.0013114 | 1E-16 | 1.01083892 |
| 11 | 61565908 | rs174541 | C | T | - | 0.00126293 | 4.4E-15 | 1.00993113 |
| 5 | 102295504 | rs2195272 | T | G | - | 0.00129125 | 6.7E-15 | 1.010175118 |
| 18 | 36105007 | rs2861805 | A | G | - | 0.00122068 | 2E-14 | 1.009354684 |
| 2 | 23611438 | rs13033444 | G | A | - | 0.00135018 | 3.6E-13 | 1.009789396 |
| 10 | 13868855 | rs11258652 | C | A | - | 0.00142918 | 3.7E-13 | 1.010454672 |
| 16 | 56129244 | rs1592544 | C | T | - | 0.00122088 | 8.5E-13 | 1.008731297 |
| 17 | 35595368 | rs12451365 | C | T | - | 0.00150388 | 1.5E-12 | 1.010684678 |
| 1 | 174864554 | rs542011541 | CT | C | - | 0.00144419 | 3.1E-12 | 1.010170572 |
| 6 | 84328832 | rs749865489 | CA | C | - | 0.00123023 | 3.8E-12 | 1.00854008 |
| 14 | 29721044 | rs10149986 | G | T | - | 0.00156608 | 4.4E-12 | 1.010998443 |
| 1 | 33342304 | rs2786547 | C | T | - | 0.00158775 | 1E-11 | 1.010825476 |
| 2 | 59478517 | rs11125776 | T | G | - | 0.00172495 | 1.5E-11 | 1.011718328 |
| 2 | 52934600 | rs350785 | T | C | - | 0.00190975 | 1.5E-11 | 1.012728223 |
| 13 | 107820389 | rs2769916 | A | G | - | 0.00131344 | 1.7E-11 | 1.008791702 |
| 3 | 84665393 | rs1601440 | C | T | - | 0.00135575 | 1.9E-11 | 1.009168304 |
| 15 | 83235408 | rs17158413 | A | G | - | 0.00142955 | 4.4E-11 | 1.009293055 |
| 12 | 38616581 | rs35011311 | G | T | - | 0.00138249 | 4.4E-11 | 1.009123366 |
| 17 | 79084367 | rs3935190 | A | G | - | 0.00122354 | 5.4E-11 | 1.008046521 |
| 18 | 23084118 | rs962247 | G | A | - | 0.00122493 | 5.7E-11 | 1.007995343 |
| 12 | 108334477 | rs60222088 | C | A | - | 0.00172293 | 6.4E-11 | 1.011299159 |
| 4 | 85408000 | rs13150944 | G | A | - | 0.0013218 | 6.8E-11 | 1.008637853 |
| 9 | 86367994 | rs141957346 | AT | A | - | 0.0012 | 7.1E-11 | 1.008132894 |
| 1 | 171516863 | rs35039375 | G | A | - | 0.00210973 | 8.7E-11 | 1.013736592 |
| 6 | 38470087 | rs4236060 | T | C | - | 0.00137545 | 8.8E-11 | 1.008864338 |
| 2 | 164574344 | rs80163246 | C | T | - | 0.00188438 | 1.4E-10 | 1.011984141 |
| 10 | 64552010 | rs224111 | G | A | - | 0.00124912 | 1.6E-10 | 1.008016068 |
| 3 | 183995341 | rs1001817 | C | T | - | 0.00121418 | 1.7E-10 | 1.007784402 |
| 11 | 28866669 | rs10835420 | T | A | - | 0.00140206 | 1.7E-10 | 1.008937725 |
| 14 | 79602542 | rs2370926 | T | C | - | 0.00126359 | 1.7E-10 | 1.008165286 |
| 21 | 47397586 | rs1883048 | C | T | - | 0.00122313 | 1.8E-10 | 1.007902491 |
| 5 | 62760828 | rs12657723 | T | C | - | 0.00129849 | 2E-10 | 1.008216764 |
| 5 | 87712831 | rs6452787 | A | G | - | 0.0012169 | 2E-10 | 1.007759258 |
| 5 | 112030173 | rs2099810 | A | G | - | 0.00121447 | 2.8E-10 | 1.007721579 |
| 14 | 29218473 | rs4983329 | A | C | - | 0.0012207 | 3.1E-10 | 1.007702019 |
| 2 | 58905715 | rs17049683 | G | A | - | 0.00129066 | 4.1E-10 | 1.007983409 |
| 4 | 79550425 | rs9998136 | G | C | - | 0.00140269 | 4.4E-10 | 1.008854935 |
| 16 | 10134637 | rs60920123 | G | A | - | 0.00122697 | 4.5E-10 | 1.007667022 |
| 1 | 96928273 | rs1931175 | G | C | - | 0.00124756 | 4.6E-10 | 1.007793462 |
| 8 | 106108303 | rs285815 | T | A | - | 0.00121983 | 5.8E-10 | 1.007558512 |
| 11 | 92769748 | rs271057 | C | T | - | 0.00140414 | 6.5E-10 | 1.008705504 |
| 2 | 49413860 | rs13023587 | C | G | - | 0.0012 | 6.5E-10 | 1.007427448 |
| 8 | 28191306 | rs351776 | C | A | - | 0.00122072 | 8.4E-10 | 1.007582271 |
| 7 | 99188589 | rs10257273 | A | T | - | 0.00170413 | 8.9E-10 | 1.010794444 |
| 6 | 100079774 | rs9389556 | G | C | - | 0.00138285 | 9.7E-10 | 1.008450677 |
| 7 | 106931292 | rs113960908 | GGT | G | - | 0.00145069 | 0.000000001 | 1.008953031 |
| 9 | 73504992 | rs1415218 | T | C | - | 0.00135243 | 1.2E-09 | 1.008240739 |
| 12 | 13493906 | rs2417268 | T | A | - | 0.00123523 | 1.2E-09 | 1.007550996 |
| 6 | 36224315 | rs34262487 | C | A | - | 0.00235463 | 1.2E-09 | 1.014616491 |
| 9 | 108846445 | rs971415 | A | G | - | 0.00185108 | 1.2E-09 | 1.011126646 |
| 3 | 19377311 | rs77154532 | A | G | - | 0.0012705 | 1.4E-09 | 1.007722909 |
| 1 | 8503379 | rs301817 | A | C | - | 0.0012 | 1.6E-09 | 1.00732671 |
| 6 | 28199764 | rs11967137 | A | G | - | 0.00153285 | 1.7E-09 | 1.009322466 |
| 20 | 39832628 | rs17265513 | C | T | - | 0.00151965 | 0.000000002 | 1.009173703 |
| 4 | 170228549 | rs4692709 | C | T | - | 0.00122517 | 2.4E-09 | 1.00726602 |
| 6 | 57698806 | rs113493369 | C | CA | - | 0.00122966 | 2.8E-09 | 1.00736921 |
| 2 | 58171287 | rs1518394 | G | A | - | 0.00123975 | 2.8E-09 | 1.007380613 |
| 16 | 69403012 | rs528301822 | T | A | - | 0.00134223 | 2.8E-09 | 1.008066712 |
| 9 | 20962282 | rs10811438 | G | C | - | 0.0012428 | 3.2E-09 | 1.007242813 |
| 1 | 98589715 | rs1843815 | T | A | - | 0.00121793 | 3.3E-09 | 1.007300399 |
| 2 | 44557919 | rs9309116 | T | C | - | 0.00127277 | 3.9E-09 | 1.007406433 |
| 3 | 82774831 | rs9883093 | G | T | - | 0.00125779 | 4.5E-09 | 1.007368011 |
| 16 | 23837048 | rs9939355 | C | T | - | 0.00122438 | 4.5E-09 | 1.007290487 |
| 20 | 45841052 | rs910187 | G | A | - | 0.00125594 | 4.9E-09 | 1.007344379 |
| 9 | 131840856 | rs12346996 | T | C | - | 0.00136459 | 0.000000005 | 1.007992833 |
| 15 | 63793936 | rs11071755 | G | A | - | 0.00122819 | 5.2E-09 | 1.007139375 |
| 1 | 162876200 | rs12031519 | A | G | - | 0.00194275 | 5.5E-09 | 1.011347702 |
| 1 | 32091779 | rs6663012 | T | G | - | 0.00124718 | 5.5E-09 | 1.007231331 |
| 5 | 106861892 | rs388016 | A | G | - | 0.00128736 | 6.1E-09 | 1.007408649 |
| 5 | 89597733 | rs2943023 | C | T | - | 0.00123055 | 7.4E-09 | 1.007129515 |
| 6 | 114638966 | rs35127490 | CG | C | - | 0.00122501 | 8.3E-09 | 1.00706359 |
| 2 | 162573586 | rs62189006 | A | G | - | 0.0020982 | 8.3E-09 | 1.012167732 |
| 6 | 170621249 | rs9460110 | C | T | - | 0.00125751 | 8.4E-09 | 1.007382799 |
| 16 | 28350059 | rs3986805 | G | A | - | 0.00123765 | 8.7E-09 | 1.007220292 |
| 6 | 62182204 | rs2143792 | G | A | - | 0.00123401 | 9.1E-09 | 1.007159568 |
| 15 | 93510243 | rs10152428 | C | G | - | 0.00136996 | 0.000000011 | 1.007837433 |
| 11 | 101479583 | rs11224896 | T | C | - | 0.00193872 | 0.000000011 | 1.011223011 |
| 2 | 208941004 | rs7422655 | C | T | - | 0.00137827 | 0.000000011 | 1.007864242 |
| 2 | 169092428 | rs35144585 | T | A | - | 0.00182249 | 0.000000012 | 1.010363836 |
| 5 | 143769614 | rs10875606 | C | A | - | 0.0013077 | 0.000000013 | 1.007404348 |
| 2 | 25337155 | rs56180058 | C | T | - | 0.00165866 | 0.000000013 | 1.009470726 |
| 5 | 106311039 | rs4357022 | G | T | - | 0.00121553 | 0.000000014 | 1.006847296 |
| 9 | 120555845 | rs4604518 | G | A | - | 0.00122091 | 0.000000014 | 1.006935993 |
| 2 | 206831860 | rs3732085 | C | A | - | 0.00126622 | 0.000000015 | 1.007233708 |
| 8 | 142213997 | rs7814873 | C | T | - | 0.00126385 | 0.000000015 | 1.007235773 |
| 8 | 1175131 | rs13263535 | G | T | - | 0.00121641 | 0.000000016 | 1.006895012 |
| 21 | 40505569 | rs2836909 | G | A | - | 0.00131199 | 0.000000016 | 1.007502403 |
| 3 | 62459819 | rs76824303 | A | C | - | 0.00207749 | 0.000000017 | 1.011867567 |
| 17 | 64308310 | rs112520848 | C | G | - | 0.00125191 | 0.000000018 | 1.00704922 |
| 3 | 138115770 | rs253666 | A | G | - | 0.0014309 | 0.000000018 | 1.008160215 |
| 9 | 37441650 | rs17502738 | T | C | - | 0.00153004 | 0.00000002 | 1.008742585 |
| 2 | 151501545 | rs12614085 | C | T | - | 0.0022 | 0.00000002 | 1.012375956 |
| 20 | 62194103 | rs3810484 | A | G | - | 0.001222 | 0.000000022 | 1.006864926 |
| 8 | 67381858 | rs2059639 | C | T | - | 0.0012172 | 0.000000024 | 1.006704676 |
| 9 | 34081331 | rs62560863 | T | C | - | 0.0020241 | 0.000000025 | 1.011381482 |
| 1 | 34998659 | rs11383197 | AG | A | - | 0.00129454 | 0.000000027 | 1.007216273 |
| 6 | 79539632 | rs10639111 | TGAGA | T | - | 0.0015 | 0.000000029 | 1.008132894 |
| 5 | 100809375 | rs189489 | C | G | - | 0.00148659 | 0.00000003 | 1.00834659 |
| 3 | 133032892 | rs2699869 | A | C | - | 0.00121774 | 0.000000031 | 1.006803882 |
| 3 | 44385814 | rs936944 | G | A | - | 0.00164374 | 0.000000032 | 1.009177134 |
| 1 | 95783037 | rs2893323 | A | G | - | 0.00126675 | 0.000000033 | 1.006965749 |
| 7 | 31330785 | rs35851551 | A | G | - | 0.00202654 | 0.000000035 | 1.011256887 |
| 6 | 51829890 | rs927184 | A | T | - | 0.0013 | 0.000000035 | 1.007225982 |
| 12 | 34855113 | rs11502903 | A | G | - | 0.00137455 | 0.000000036 | 1.007611834 |
| 3 | 85388164 | rs3943782 | G | A | - | 0.00126436 | 0.000000036 | 1.007020751 |
| 18 | 31610848 | rs34728579 | C | T | - | 0.00152087 | 0.000000037 | 1.008435339 |
| 15 | 83752159 | rs2084839 | G | T | - | 0.00123268 | 0.000000038 | 1.006723009 |
| 5 | 159042652 | rs73817091 | T | C | - | 0.00302224 | 0.000000038 | 1.016649788 |
| 18 | 25569743 | rs1941182 | A | C | - | 0.00124346 | 0.000000041 | 1.006817705 |
| 22 | 37096573 | rs2284015 | G | C | - | 0.00138662 | 0.000000041 | 1.007741482 |
| 6 | 155129280 | rs140506252 | A | T | - | 0.0040886 | 0.000000043 | 1.022986305 |
| 4 | 164216739 | rs4356873 | C | T | - | 0.00142205 | 0.000000045 | 1.007787053 |

^a^Chr, chromosome; BP, basepair position; SNP, single nucleotide polymorphism; A1, effect allele; A2, non-effect allele; N, number of participants; SE, standard error; P, two-sided P-value; OR, odd ratio for the association of SNP with outcome.

**Supplementary table 6.** Genome-wide association signals for snoring in participants of European ancestry from the UK Biobank (n = 452,633)^a^

| CHR | BP | SNP | A1 | A2 | N | SE | P | OR |
| --- | --- | --- | --- | --- | --- | --- | --- | --- |
| 13 | 51340315 | rs592333 | A | G | - | 0.0010512 | 1E-17 | 0.99098296 |
| 12 | 65791463 | rs10878269 | C | T | - | 0.00108623 | 2.3E-16 | 0.991182673 |
| 2 | 156996626 | rs61597598 | G | A | - | 0.0015292 | 5.1E-15 | 0.988175762 |
| 5 | 75003678 | rs2307111 | T | C | - | 0.00106982 | 4.8E-13 | 1.0076969 |
| 14 | 99742187 | rs2664299 | T | C | - | 0.00106064 | 1.1E-12 | 1.007531158 |
| 8 | 71474355 | rs13251292 | A | G | - | 0.00106717 | 4.3E-12 | 0.992652407 |
| 17 | 43758898 | rs57222984 | A | G | - | 0.00122014 | 5.4E-12 | 0.991600951 |
| 10 | 9063776 | rs725861 | A | G | - | 0.00133779 | 1E-11 | 0.990959137 |
| 1 | 96878072 | rs12119849 | G | A | - | 0.00186046 | 4.1E-11 | 0.987818601 |
| 16 | 53799278 | rs796856741 | G | GT | - | 0.00105918 | 4.7E-11 | 0.993063092 |
| 13 | 40745860 | rs12429765 | A | G | - | 0.0010508 | 6.2E-11 | 1.006822689 |
| 4 | 25408838 | rs34811474 | G | A | - | 0.00123692 | 1.3E-10 | 1.008028326 |
| 8 | 78215352 | rs7829639 | A | G | - | 0.00115501 | 1.4E-10 | 0.992614627 |
| 17 | 67930772 | rs180107 | A | T | - | 0.00105996 | 2.1E-10 | 0.993223862 |
| 17 | 46269542 | rs11409890 | T | TA | - | 0.00106125 | 2.2E-10 | 1.006686133 |
| 17 | 1985843 | rs8069947 | C | T | - | 0.00105133 | 2.8E-10 | 1.006627848 |
| 3 | 77599166 | rs74936745 | AC | A | - | 0.0010503 | 4E-10 | 1.006511226 |
| 11 | 88861590 | rs11018488 | A | T | - | 0.00109873 | 5.3E-10 | 1.006803409 |
| 19 | 32183171 | rs8108822 | C | T | - | 0.00178271 | 6.2E-10 | 1.010932225 |
| 5 | 134452597 | rs4976269 | G | A | - | 0.00111414 | 8.6E-10 | 1.006867393 |
| 11 | 27694241 | rs2049045 | G | C | - | 0.00134145 | 8.8E-10 | 1.008375933 |
| 16 | 31109287 | rs59502288 | G | GTCATCCA | - | 0.00109415 | 9.1E-10 | 0.99329338 |
| 17 | 7439739 | rs9900496 | T | C | - | 0.0011057 | 1.8E-09 | 0.99340995 |
| 7 | 127382155 | rs17151229 | G | C | - | 0.00110239 | 1.9E-09 | 0.993481439 |
| 12 | 57088077 | 12:57088077_CA_C | CA | C | - | 0.0011479 | 0.000000003 | 1.006830563 |
| 1 | 50819256 | 1:50819256_CA_C | CA | C | - | 0.00106313 | 3.5E-09 | 1.006306935 |
| 20 | 6635266 | rs6054427 | G | A | - | 0.00108007 | 0.000000004 | 0.993703447 |
| 3 | 94027330 | rs202110996 | A | ATT | - | 0.00108156 | 4.6E-09 | 1.00624991 |
| 3 | 90049321 | rs145367119 | C | T | - | 0.00107214 | 0.000000012 | 1.006058308 |
| 6 | 100827834 | rs17060460 | A | G | - | 0.00124704 | 0.000000014 | 0.992933724 |
| 17 | 54776955 | rs227727 | A | T | - | 0.00104952 | 0.000000014 | 0.994045399 |
| 6 | 73738661 | rs947612 | G | A | - | 0.00121018 | 0.000000015 | 1.00675159 |
| 5 | 122699198 | rs34732995 | C | CTA | - | 0.00104786 | 0.000000017 | 1.005840369 |
| 1 | 39698437 | rs80093081 | T | G | - | 0.00112958 | 0.000000018 | 1.006340768 |
| 6 | 84307328 | rs2207944 | T | C | - | 0.00106471 | 0.00000002 | 0.994072358 |
| 20 | 55347828 | rs6099273 | C | T | - | 0.00120584 | 0.000000026 | 0.993340315 |
| 2 | 103892497 | rs773118143 | ACTCTGTAT | A | - | 0.0011885 | 0.000000029 | 0.993526424 |
| 1 | 87773720 | 1:87773720_CT_C | CT | C | - | 0.00109667 | 0.00000003 | 1.006121349 |
| 18 | 60960310 | rs4987719 | C | T | - | 0.00290619 | 0.000000035 | 0.983906113 |
| 9 | 97475396 | rs4744369 | T | A | - | 0.00106803 | 0.00000004 | 0.994191684 |
| 13 | 111566412 | rs9583546 | G | C | - | 0.00108049 | 0.00000004 | 0.994149292 |

^a^Chr, chromosome; BP, basepair position; SNP, single nucleotide polymorphism; A1, effect allele; A2, non-effect allele; N, number of participants; SE, standard error; P, two-sided P-value; OR, odd ratio for the association of SNP with outcome.

**Supplementary table 7.** Genome-wide association signals for Klemera-Doubal method in participants of European ancestry from the UK Biobank (n = 452,633)^a^

| CHR | BP | SNP | A1 | A2 | N | SE | P | OR |
| --- | --- | --- | --- | --- | --- | --- | --- | --- |
| 1 | 11862778 | rs17367504 | G | A | - | 0.012 | 9.03E−10 | 1.072508181 |
| 1 | 55505647 | rs11591147 | T | G | - | 0.033 | 7.91E−13 | 1.25860001 |
| 2 | 21294975 | rs541041 | A | G | - | 0.011 | 2.33E−14 | 0.913931185 |
| 2 | 169763148 | rs560887 | C | T | - | 0.009 | 9.83E−11 | 0.941764534 |
| 4 | 81184341 | rs16998073 | T | A | - | 0.009 | 2.46E−08 | 0.951229425 |
| 5 | 32815028 | rs1173771 | G | A | - | 0.009 | 6.19E−09 | 0.951229425 |
| 7 | 106411858 | rs17477177 | C | T | - | 0.011 | 4.62E−17 | 0.913931185 |
| 8 | 126486409 | rs17321515 | G | A | - | 0.009 | 2.20E−12 | 1.061836547 |
| 10 | 71093392 | rs16926246 | T | C | - | 0.013 | 7.77E−13 | 1.094174284 |
| 10 | 96039597 | rs2274224 | C | G | - | 0.009 | 2.41E−10 | 1.051271096 |
| 12 | 90060586 | rs17249754 | A | G | - | 0.011 | 9.41E−09 | 1.072508181 |
| 15 | 91429176 | rs7497304 | T | G | - | 0.009 | 1.89E−08 | 0.951229425 |
| 19 | 11188153 | rs55791371 | C | A | - | 0.013 | 4.95E−26 | 1.150273799 |
| 19 | 19379549 | rs58542926 | T | C | - | 0.016 | 1.78E−11 | 1.11627807 |
| 19 | 45412079 | rs7412 | T | C | - | 0.016 | 3.16E−60 | 1.296930087 |
| 20 | 10969030 | rs1327235 | G | A | - | 0.009 | 1.02E−08 | 0.951229425 |

^a^Chr, chromosome; BP, basepair position; SNP, single nucleotide polymorphism; A1, effect allele; A2, non-effect allele; N, number of participants; SE, standard error; P, two-sided P-value; OR, odd ratio for the association of SNP with outcome.

**Supplementary table 8.** Genome-wide association signals for predicted age in participants of European ancestry from the UK Biobank (n = 452,633)^a^

| CHR | BP | SNP | A1 | A2 | N | SE | P | OR |
| --- | --- | --- | --- | --- | --- | --- | --- | --- |
| 1 | 11856378 | rs1801133 | A | G | - | 0.022 | 1.28E−09 | 0.878095431 |
| 1 | 40064961 | rs12037222 | A | G | - | 0.025 | 1.98E−15 | 0.818730753 |
| 1 | 66102257 | rs1805096 | A | G | - | 0.021 | 1.01E−20 | 1.221402758 |
| 1 | 154426264 | rs4129267 | T | C | - | 0.021 | 6.73E−15 | 1.173510871 |
| 1 | 159698549 | rs7553007 | A | G | - | 0.022 | 2.44E−17 | 1.209249598 |
| 1 | 247601595 | rs12239046 | C | T | - | 0.021 | 1.40E−08 | 0.886920437 |
| 1 | 248039451 | rs3811444 | T | C | - | 0.022 | 2.61E−16 | 1.197217363 |
| 2 | 27730940 | rs1260326 | C | T | - | 0.021 | 2.29E−09 | 0.878095431 |
| 2 | 113841030 | rs6734238 | G | A | - | 0.021 | 1.51E−10 | 0.878095431 |
| 2 | 169763148 | rs560887 | C | T | - | 0.023 | 1.72E−15 | 0.835270211 |
| 5 | 1104938 | rs35188965 | G | A | - | 0.021 | 5.45E−13 | 1.161834243 |
| 6 | 135418635 | rs7775698 | T | C | - | 0.024 | 9.60E−12 | 1.173510871 |
| 6 | 139840693 | rs592423 | T | C | - | 0.021 | 3.38E−11 | 1.150273799 |
| 8 | 126486409 | rs17321515 | C | A | - | 0.021 | 4.08E−12 | 0.869358235 |
| 9 | 136131322 | rs8176746 | A | G | - | 0.043 | 4.54E−11 | 1.323129812 |
| 10 | 45953767 | rs7908745 | C | T | - | 0.022 | 1.31E−09 | 0.869358235 |
| 10 | 71093392 | rs16926246 | G | A | - | 0.031 | 1.46E−10 | 0.818730753 |
| 11 | 61571348 | rs174548 | T | G | - | 0.022 | 8.18E−31 | 1.296930087 |
| 11 | 116648917 | rs964184 | G | A | - | 0.03 | 3.28E−08 | 0.843664817 |
| 12 | 121423956 | rs2393791 | T | C | - | 0.021 | 7.45E−12 | 0.860707976 |
| 14 | 23494277 | rs8013143 | G | C | - | 0.023 | 4.66E−14 | 0.843664817 |
| 15 | 78563103 | rs3169166 | C | G | - | 0.021 | 1.34E−14 | 1.173510871 |
| 16 | 53820527 | rs9939609 | T | C | - | 0.021 | 1.62E−13 | 0.852143789 |
| 17 | 27183104 | rs9914988 | C | A | - | 0.026 | 3.20E−08 | 0.869358235 |
| 17 | 38166879 | rs8078723 | A | T | - | 0.021 | 6.25E−23 | 0.810584246 |
| 18 | 43831259 | rs9944715 | G | A | - | 0.024 | 6.09E−11 | 0.852143789 |
| 19 | 18513594 | rs1985157 | C | T | - | 0.021 | 1.10E−10 | 0.869358235 |
| 19 | 45411941 | rs429358 | C | T | - | 0.029 | 1.50E−72 | 1.68202765 |
| 19 | 45412079 | rs7412 | T | C | - | 0.038 | 3.07E−21 | 0.697676326 |

^a^Chr, chromosome; BP, basepair position; SNP, single nucleotide polymorphism; A1, effect allele; A2, non-effect allele; N, number of participants; SE, standard error; P, two-sided P-value; OR, odd ratio for the association of SNP with outcome.

**Supplementary table 9.** Associations of the odds of different sleep behaviors and predicted age metrics with polygenic risk scores at baseline^a^

| Trait | Coefficients | SE | R-squares (variance explained) | *P* |
| --- | --- | --- | --- | --- |
| Morning person | 0.167 | 0.009 | 0.029 | < 0.001 |
| Insomnia | 0.048 | 0.010 | 0.051 | < 0.001 |
| Daytime sleepiness | 0.126 | 0.026 | 0.013 | < 0.001 |
| Snoring | -0.075 | 0.009 | 0.088 | < 0.001 |
| Sleep duration | 1.914 | 0.459 | 0.010 | < 0.001 |
| Klemera-Doubal method | -39.973 | 1.551 | 0.771 | < 0.001 |
| PhenoAge | -6.427 | 0.369 | 0.882 | < 0.001 |

^a^Associations of morning person, insomnia, daytime sleepiness, and snoring at baseline with polygenic risk scores were tested by logistic regression models. Associations of sleep duration, Klemera-Doubal method, and phenoage at baseline with polygenic risk scores were tested by linear regression models. Models were adjusted for age, sex, ethnicity, BMI, smoking, drinking, regular exercise, education, Townsend deprivation index, diet score, overall health rating, self-reported diabetes, hypertension, CVD, cancer, medication for cholesterol, blood pressure or diabetes, family history of diabetes, hypertension, CVD, cancer, sleep disorders, depression and shift work.

**Supplementary table 10.** Original questions and definitions for covariates

| Variable | UK Biobank code | UK Biobank Questionnaire/Definition |
| --- | --- | --- |
| Body mass index | 21001 | BMI value here is constructed from height and weight measured during the initial Assessment Centre visit. |
| Physical activity^a^ | 864, 874, 884, 894, 904, 914 | The UK Biobank applied a modified-version short-form International Physical Activity Questionnaire (IPAQ). We summarized weekly physical activity (PA) using weekly total Metabolic Equivalent Task (MET), calculated by multiplying the MET value of activity by the number of PA hours per week. Based on the lower and upper limits of the World Health Organization (WHO) PA guideline, we categorized PA into three groups. Another category “No moderate-to-vigorous PA” was further defined. See https://biobank.ndph.ox.ac.uk/showcase/label.cgi?id=100054 for full questionnaire details. Low (0 to 600 MET-mins/wk); Moderate (600 to 1200 MET-mins/wk); High (≥ 1200 MET-mins/wk). |
| Diet quality score^b^ | 1289, 1299, 1309, 1319, 1329, 1339, 1349, 1369, 1379, 1389 | Four healthy diet patterns were evaluated based on the American Heart Asociation (AHA), > 4.5 servings/d fruits and vegetables, >= 2 times/wk fish intake, < 2 times/wk processed meat, < 5 times/wk red meat intake. Participants got one score for each criterion. We then categorized them based on the score. See https://biobank.ndph.ox.ac.uk/showcase/label.cgi?id=100052 for full questionnaire details. |
| Type 2 diabetes^c^ | 20002, 41270, 40001, 40002 | Self-reported medical history and medication information, hospital inpatient records (ICD - 9 code 250.00, 250.10, 250.20, and 250.90 and ICD - 10 code E11). |
| Hypertension^d^ | 1065, 20002, 41270, 40001, 40002 | Self-reported medical history and medication information, hospital inpatient records (ICD - 9 code 401 - 405 and ICD - 10 code I10 - I13, I15, O10). |
| Cardiovascular diseases^b^ | 20002, 41270, 40001, 40002 | Self-reported medical history and medication information, hospital inpatient records (ICD - 10 code I00 - I99). |
| Cancer^e^ | 20002, 41270, 40001, 40002 | Self-reported medical history and medication information, hospital inpatient records (ICD - 10 code C00 - C97). |
| Sleep disorders^f^ | 20002, 41270, 40001, 40002 | Self-reported medical history and medication information, hospital inpatient records (ICD - 10 code G47, F51). |
| Depression^g^ | 20002, 41270, 40001, 40002 | Self-reported medical history and medication information, hospital inpatient records (ICD - 9 code 311 and ICD - 10 code F32 - F33). |

^a^Cassidy S, Chau JY, Catt M, Bauman A, Trenell MI: Cross-sectional study of diet, physical activity, television viewing and sleep duration in 233,110 adults from the UK Biobank; the behavioural phenotype of cardiovascular disease and type 2 diabetes. BMJ open 2016, 6(3):e010038.

^b^Rutten-Jacobs LC, Larsson SC, Malik R, Rannikmäe K, Sudlow CL, Dichgans M, Markus HS, Traylor M: Genetic risk, incident stroke, and the benefits of adhering to a healthy lifestyle: cohort study of 306 473 UK Biobank participants. BMJ (Clinical research ed) 2018, 363:k4168.

^c^Han H, Wang Y, Li T, Feng C, Kaliszewski C, Su Y, Wu Y, Zhou J, Wang L, Zong G: Sleep Duration and Risks of Incident Cardiovascular Disease and Mortality Among People With Type 2 Diabetes. Diabetes care 2023, 46(1):101-110.

^d^Shang X, Hill E, Zhu Z, Liu J, Ge BZ, Wang W, He M: The Association of Age at Diagnosis of Hypertension With Brain Structure and Incident Dementia in the UK Biobank. Hypertension (Dallas, Tex : 1979) 2021, 78(5):1463-1474.

^e^Bai L, Xu Z, Huang C, Sui Y, Guan X, Shi L: Psychotropic medication utilisation in adult cancer patients in China: A cross-sectional study based on national health insurance database. The Lancet regional health Western Pacific 2020, 5:100060.

^f^Huang BH, Del Pozo Cruz B, Teixeira-Pinto A, Cistulli PA, Stamatakis E: Influence of poor sleep on cardiovascular disease-free life expectancy: a multi-resource-based population cohort study. BMC medicine 2023, 21(1):75.

^g^Gao X, Geng T, Jiang M, Huang N, Zheng Y, Belsky DW, Huang T: Accelerated biological aging and risk of depression and anxiety: evidence from 424,299 UK Biobank participants. Nature communications 2023, 14(1):2277.

**Supplementary table 11.** Original questions for self-reported sleep characteristics

| Characteristic | UK Biobank code | UK Biobank questionnaire | Healthy answer | Unhealthy answer |
| --- | --- | --- | --- | --- |
| Insomnia complaint | 1200 | Do you have trouble falling asleep at night or do you wake up in the middle of the night? | Never/rarely; Sometimes | Usually |
| Sleep duration | 1160 | About how many hours sleep do you get in every 24 hours? (please include naps) (in integer). | 7 to < 9 h/d | < 7 or >= 9 h/d |
| Snoring | 1210 | Does your partner or a close relative or friend complain about your Snoring? | No | Yes |
| Chronotype | 1180 | Do you consider yourself to be? | Definitely a “morning” person; More a “morning” than “evening” person | More an “evening” than a “morning person;Definitely an “evening” person |
| Daytime sleepiness | 1220 | How likely are you to doze off or fall asleep during the daytime when you don't mean to? (e.g. when working, reading or driving) | Never/rarely; Sometimes | Often; All the Time |

**Supplementary table 12.** Associations between sleep duration and predicted age metrics

|  |  | Habitual sleep duration, h/day | | | | | | | | |  |
| --- | --- | --- | --- | --- | --- | --- | --- | --- | --- | --- | --- |
|  |  | ≤ 5 (n = 10,641) | | 6 (n = 43,154) | | 7 (n = 95,469) | 8 (n = 73,640) | | ≥ 9 (n = 18,809) | | *P*trend |
|  |  | β | 95 CI | β | 95 CI | β | β | 95 CI | β | 95 CI |  |
| Homeostatic dysregulation |  |  |  |  |  |  |  |  |  |  |  |
|  | Model 1 | 0.15 | **(0.13, 0.17)** | 0.04 | **(0.03, 0.05)** | Ref (0.00) | 0.02 | **(0.01, 0.03)** | 0.10 | **(0.09, 0.12)** | < 0.001 |
|  | Model 2 | 0.09 | **(0.07, 0.11)** | 0.01 | (0.00, 0.02) | Ref (0.00) | 0.02 | **(0.01, 0.03)** | 0.07 | **(0.05, 0.08)** | < 0.001 |
|  | Model 3 | 0.04 | **(0.02, 0.06)** | 0.00 | (-0.01, 0.01) | Ref (0.00) | 0.01 | **(0.01, 0.02)** | 0.03 | **(0.02, 0.04)** | < 0.001 |
|  | Model 4 | 0.05 | **(0.03, 0.07)** | 0.00 | (-0.01, 0.01) | Ref (0.00) | 0.01 | **(0.01, 0.02)** | 0.03 | **(0.02, 0.05)** | < 0.001 |
| PA residual |  |  |  |  |  |  |  |  |  |  |  |
|  | Model 1 | 0.87 | **(0.80, 0.94)** | 0.29 | **(0.24, 0.33)** | Ref (0.00) | 0.18 | **(0.14, 0.21)** | 0.87 | **(0.81, 0.92)** | < 0.001 |
|  | Model 2 | 0.31 | **(0.24, 0.37)** | 0.03 | (-0.01, 0.07) | Ref (0.00) | 0.15 | **(0.12, 0.19)** | 0.52 | **(0.47, 0.57)** | < 0.001 |
|  | Model 3 | 0.05 | (-0.01, 0.12) | -0.04 | (-0.08, 0.00) | Ref (0.00) | 0.14 | **(0.11, 0.17)** | 0.35 | **(0.30, 0.40)** | < 0.001 |
|  | Model 4 | 0.08 | **(0.01, 0.14)** | -0.03 | (-0.07, 0.00) | Ref (0.00) | 0.14 | **(0.11, 0.17)** | 0.36 | **(0.31, 0.41)** | < 0.001 |
| KDM residual |  |  |  |  |  |  |  |  |  |  |  |
|  | Model 1 | 0.81 | **(0.71, 0.90)** | 0.28 | **(0.22, 0.33)** | Ref (0.00) | 0.18 | **(0.14, 0.23)** | 0.69 | **(0.61, 0.76)** | < 0.001 |
|  | Model 2 | 0.31 | **(0.22, 0.40)** | 0.04 | (-0.02, 0.09) | Ref (0.00) | 0.15 | **(0.11, 0.20)** | 0.38 | **(0.30, 0.45)** | < 0.001 |
|  | Model 3 | 0.15 | **(0.06, 0.24)** | 0.00 | (-0.05, 0.05) | Ref (0.00) | 0.14 | **(0.10, 0.19)** | 0.27 | **(0.20, 0.34)** | < 0.001 |
|  | Model 4 | 0.21 | **(0.12, 0.30)** | 0.01 | (-0.04, 0.06) | Ref (0.00) | 0.15 | **(0.10, 0.19)** | 0.30 | **(0.23, 0.37)** | < 0.001 |
| Allostatic load |  |  |  |  |  |  |  |  |  |  |  |
|  | Model 1 | 0.03 | **(0.03, 0.04)** | 0.01 | **(0.01, 0.02)** | Ref (0.00) | 0.01 | (0.00, 0.01) | 0.03 | **(0.03, 0.03)** | < 0.001 |
|  | Model 2 | 0.01 | **(0.01, 0.01)** | 0.00 | (0.00, 0.01) | Ref (0.00) | 0.00 | (0.00, 0.01) | 0.01 | **(0.01, 0.02)** | < 0.001 |
|  | Model 3 | 0.00 | (0.00, 0.01) | 0.00 | (0.00, 0.00) | Ref (0.00) | 0.00 | (0.00, 0.01) | 0.01 | **(0.01, 0.01)** | < 0.001 |
|  | Model 4 | 0.01 | (0.00, 0.01) | 0.00 | (0.00, 0.00) | Ref (0.00) | 0.00 | (0.00, 0.01) | 0.01 | **(0.01, 0.01)** | < 0.001 |

^a^Model 1 was adjusted for age, sex, ethnicity. Model 2 further adjusted for BMI, smoking, drinking, regular exercise, education, Townsend deprivation index, diet score. Model 3 further adjusted for self-reported diabetes, hypertension, CVD, cancer, medication for cholesterol, blood pressure or diabetes, family history of diabetes, hypertension, CVD, cancer. Model 4 further adjusted for sleep disorders, depression and shift work.

^b^Data were listed as beta estimates and 95% confidence intervals, with the bold font lower than the adjusted critical level of significance using Bonferroni’s correction.

^c^Ref, reference.

^d^Tests for trends based on the variables containing the median values for each group.

**Supplementary table 13.** Associations between sleep duration and cystatin C (CysC) and gamma glutamyltransferase (GGT)

|  |  | Habitual sleep duration, h/day | | | | | | | | |  |
| --- | --- | --- | --- | --- | --- | --- | --- | --- | --- | --- | --- |
|  |  | ≤ 5 (n = 10,641) | | 6 (n = 43,154) | | 7 (n = 95,469) | 8 (n = 73,640) | | ≥ 9 (n = 18,809) | | *P*_trend_ |
|  |  | β | 95 CI | β | 95 CI | β | β | 95 CI | β | 95 CI |  |
| Cystatin C |  |  |  |  |  |  |  |  |  |  |  |
|  | Model 1 | 0.04 | **(0.04, 0.04)** | 0.01 | **(0.01, 0.01)** | Ref (0.00) | 0.00 | (0.00, 0.01) | 0.03 | **(0.03, 0.03)** | < 0.001 |
|  | Model 2 | 0.02 | **(0.02, 0.02)** | 0.00 | (0.00, 0.00) | Ref (0.00) | 0.00 | (0.00, 0.01) | 0.02 | **(0.02, 0.02)** | < 0.001 |
|  | Model 3 | 0.01 | **(0.01, 0.01)** | 0.00 | (0.00, 0.00) | Ref (0.00) | 0.00 | (0.00, 0.01) | 0.02 | **(0.01, 0.02)** | < 0.001 |
|  | Model 4 | 0.01 | **(0.01, 0.01)** | 0.00 | (0.00, 0.00) | Ref (0.00) | 0.00 | (0.00, 0.01) | 0.02 | **(0.01, 0.02)** | < 0.001 |
| Gamma glutamyltransferase |  |  |  |  |  |  |  |  |  |  |  |
|  | Model 1 | 7.84 | **(7.04, 8.65)** | 2.64 | **(2.19, 3.10)** | Ref (0.00) | 1.07 | **(0.68, 1.45)** | 5.76 | **(5.13, 6.39)** | < 0.001 |
|  | Model 2 | 5.41 | **(4.61, 6.20)** | 1.50 | **(1.05, 1.95)** | Ref (0.00) | 1.00 | **(0.62, 1.38)** | 4.25 | **(3.63, 4.87)** | < 0.001 |
|  | Model 3 | 3.71 | **(2.92, 4.51)** | 1.00 | **(0.55, 1.44)** | Ref (0.00) | 0.95 | **(0.57, 1.33)** | 3.20 | **(2.58, 3.82)** | < 0.001 |
|  | Model 4 | 3.57 | **(2.77, 4.37)** | 0.98 | **(0.53, 1.43)** | Ref (0.00) | 0.93 | **(0.55, 1.30)** | 3.08 | **(2.46, 3.70)** | < 0.001 |

^a^Model 1 was adjusted for age, sex, ethnicity. Model 2 further adjusted for BMI, smoking, drinking, regular exercise, education, Townsend deprivation index, diet score. Model 3 further adjusted for self-reported diabetes, hypertension, CVD, cancer, medication for cholesterol, blood pressure or diabetes, family history of diabetes, hypertension, CVD, cancer. Model 4 further adjusted for sleep disorders, depression and shift work.

^b^Data were listed as beta estimates and 95% confidence intervals, with the bold font lower than the adjusted critical level of significance using Bonferroni’s correction.

^c^Ref, reference.

^d^Tests for trends based on the variables containing the median values for each group.

**Supplementary table 14.** Associations between sleep duration and predicted age metrics among participants who were not employed or employed without night shifts at baseline

|  |  | Habitual sleep duration, h/day | | | | | | | | |  |  |
| --- | --- | --- | --- | --- | --- | --- | --- | --- | --- | --- | --- | --- |
|  |  | ≤ 5 (n = 10,641) | | 6 (n = 43,154) | | 7 (n = 95,469) | 8 (n = 73,640) | | ≥ 9 (n = 18,809) | | *P*trend | *P*interaction |
|  |  | β | 95 CI | β | 95 CI | β | β | 95 CI | β | 95 CI |  |  |
| Homeostatic dysregulation |  |  |  |  |  |  |  |  |  |  |  |  |
|  | Model 1 | 0.15 | **(0.13, 0.17)** | 0.03 | **(0.02, 0.05)** | Ref (0.00) | 0.02 | **(0.01, 0.03)** | 0.11 | **(0.09, 0.12)** | < 0.001 | < 0.001 |
|  | Model 2 | 0.09 | **(0.07, 0.11)** | 0.01 | (0.00, 0.02) | Ref (0.00) | 0.02 | **(0.01, 0.03)** | 0.07 | **(0.05, 0.09)** | < 0.001 | 0.015 |
|  | Model 3 | 0.04 | **(0.02, 0.06)** | 0.00 | (-0.02, 0.01) | Ref (0.00) | 0.01 | (0.00, 0.02) | 0.03 | **(0.02, 0.05)** | 0.001 | 0.106 |
|  | Model 4 | 0.05 | **(0.03, 0.07)** | 0.00 | (-0.01, 0.01) | Ref (0.00) | 0.01 | (0.00, 0.02) | 0.04 | **(0.02, 0.05)** | < 0.001 | 0.105 |
| PA residual |  |  |  |  |  |  |  |  |  |  |  |  |
|  | Model 1 | 0.85 | **(0.76, 0.93)** | 0.25 | **(0.21, 0.30)** | Ref (0.00) | 0.20 | **(0.16, 0.24)** | 0.92 | **(0.85, 0.98)** | < 0.001 | < 0.001 |
|  | Model 2 | 0.29 | **(0.21, 0.36)** | 0.01 | (-0.04, 0.05) | Ref (0.00) | 0.16 | **(0.12, 0.20)** | 0.55 | **(0.49, 0.61)** | < 0.001 | < 0.001 |
|  | Model 3 | 0.02 | (-0.05, 0.09) | -0.06 | **(-0.10, -0.02)** | Ref (0.00) | 0.15 | **(0.11, 0.18)** | 0.36 | **(0.31, 0.42)** | < 0.001 | < 0.001 |
|  | Model 4 | 0.05 | (-0.03, 0.12) | -0.06 | **(-0.10, -0.01)** | Ref (0.00) | 0.15 | **(0.11, 0.18)** | 0.38 | **(0.32, 0.43)** | < 0.001 | < 0.001 |
| KDM residual |  |  |  |  |  |  |  |  |  |  |  |  |
|  | Model 1 | 0.74 | **(0.63, 0.85)** | 0.22 | **(0.16, 0.29)** | Ref (0.00) | 0.20 | **(0.15, 0.26)** | 0.72 | **(0.64, 0.81)** | < 0.001 | < 0.001 |
|  | Model 2 | 0.27 | **(0.17, 0.38)** | 0.01 | (-0.05, 0.07) | Ref (0.00) | 0.16 | **(0.11, 0.21)** | 0.41 | **(0.33, 0.49)** | < 0.001 | < 0.001 |
|  | Model 3 | 0.11 | **(0.01, 0.21)** | -0.03 | (-0.09, 0.03) | Ref (0.00) | 0.15 | **(0.10, 0.20)** | 0.30 | **(0.22, 0.38)** | < 0.001 | < 0.001 |
|  | Model 4 | 0.17 | **(0.07, 0.28)** | -0.02 | (-0.08, 0.04) | Ref (0.00) | 0.16 | **(0.11, 0.20)** | 0.33 | **(0.25, 0.41)** | < 0.001 | < 0.001 |
| Allostatic load |  |  |  |  |  |  |  |  |  |  |  |  |
|  | Model 1 | 0.03 | **(0.03, 0.04)** | 0.01 | **(0.01, 0.01)** | Ref (0.00) | 0.01 | (0.00, 0.01) | 0.03 | **(0.03, 0.03)** | < 0.001 | < 0.001 |
|  | Model 2 | 0.01 | **(0.01, 0.01)** | 0.00 | (0.00, 0.00) | Ref (0.00) | 0.00 | (0.00, 0.01) | 0.02 | **(0.01, 0.02)** | < 0.001 | < 0.001 |
|  | Model 3 | 0.00 | (0.00, 0.01) | 0.00 | (0.00, 0.00) | Ref (0.00) | 0.00 | (0.00, 0.01) | 0.01 | **(0.01, 0.01)** | < 0.001 | 0.002 |
|  | Model 4 | 0.00 | (0.00, 0.01) | 0.00 | (0.00, 0.00) | Ref (0.00) | 0.00 | (0.00, 0.01) | 0.01 | **(0.01, 0.01)** | < 0.001 | 0.002 |

^a^Model 1 was adjusted for age, sex, ethnicity. Model 2 further adjusted for BMI, smoking, drinking, regular exercise, education, Townsend deprivation index, diet score. Model 3 further adjusted for self-reported diabetes, hypertension, CVD, cancer, medication for cholesterol, blood pressure or diabetes, family history of diabetes, hypertension, CVD, cancer. Model 4 further adjusted for sleep disorders, depression and shift work.

^b^Data were listed as beta estimates and 95% confidence intervals, with the bold font lower than the adjusted critical level of significance using Bonferroni’s correction.

^c^Ref, reference.

^d^Tests for trends based on the variables containing the median values for each group.

**Supplementary table 15.** Associations between sleep duration and predicted age metrics among participants who were not depression at baseline

|  |  | Habitual sleep duration, h/day | | | | | | | | |  |  |
| --- | --- | --- | --- | --- | --- | --- | --- | --- | --- | --- | --- | --- |
|  |  | ≤ 5 (n = 10,641) | | 6 (n = 43,154) | | 7 (n = 95,469) | 8 (n = 73,640) | | ≥ 9 (n = 18,809) | | *P*_trend_ | *P*_interaction_ |
|  |  | β | 95 CI | β | 95 CI | β | β | 95 CI | β | 95 CI |  |  |
| Homeostatic dysregulation |  |  |  |  |  |  |  |  |  |  |  |  |
|  | Model 1 | 0.13 | **(0.11, 0.15)** | 0.04 | **(0.03, 0.05)** | Ref (0.00) | 0.02 | **(0.01, 0.03)** | 0.09 | **(0.08, 0.11)** | < 0.001 | 0.889 |
|  | Model 2 | 0.08 | **(0.06, 0.10)** | 0.01 | (0.00, 0.02) | Ref (0.00) | 0.02 | **(0.01, 0.03)** | 0.06 | **(0.05, 0.08)** | < 0.001 | 0.351 |
|  | Model 3 | 0.04 | **(0.02, 0.06)** | 0.00 | (-0.01, 0.01) | Ref (0.00) | 0.01 | **(0.01, 0.02)** | 0.03 | **(0.02, 0.05)** | < 0.001 | 0.312 |
|  | Model 4 | 0.04 | **(0.02, 0.06)** | 0.00 | (-0.01, 0.01) | Ref (0.00) | 0.01 | **(0.01, 0.02)** | 0.03 | **(0.02, 0.05)** | < 0.001 | 0.325 |
| PA residual |  |  |  |  |  |  |  |  |  |  |  |  |
|  | Model 1 | 0.77 | **(0.70, 0.85)** | 0.26 | **(0.21, 0.30)** | Ref (0.00) | 0.17 | **(0.13, 0.20)** | 0.79 | **(0.73, 0.85)** | < 0.001 | 0.516 |
|  | Model 2 | 0.29 | **(0.21, 0.36)** | 0.02 | (-0.02, 0.06) | Ref (0.00) | 0.15 | **(0.12, 0.18)** | 0.52 | **(0.46, 0.57)** | < 0.001 | 0.114 |
|  | Model 3 | 0.07 | (0.00, 0.14) | -0.03 | (-0.07, 0.00) | Ref (0.00) | 0.14 | **(0.11, 0.18)** | 0.37 | **(0.31, 0.42)** | < 0.001 | 0.062 |
|  | Model 4 | 0.08 | **(0.01, 0.15)** | -0.03 | (-0.07, 0.01) | Ref (0.00) | 0.14 | **(0.11, 0.17)** | 0.37 | **(0.31, 0.42)** | < 0.001 | 0.063 |
| KDM residual |  |  |  |  |  |  |  |  |  |  |  |  |
|  | Model 1 | 0.78 | **(0.67, 0.88)** | 0.26 | **(0.20, 0.32)** | Ref (0.00) | 0.17 | **(0.13, 0.22)** | 0.65 | **(0.57, 0.73)** | < 0.001 | 0.556 |
|  | Model 2 | 0.33 | **(0.23, 0.43)** | 0.04 | (-0.02, 0.09) | Ref (0.00) | 0.15 | **(0.11, 0.20)** | 0.39 | **(0.32, 0.47)** | < 0.001 | 0.305 |
|  | Model 3 | 0.18 | **(0.09, 0.28)** | 0.00 | (-0.05, 0.06) | Ref (0.00) | 0.15 | **(0.10, 0.19)** | 0.30 | **(0.22, 0.37)** | < 0.001 | 0.326 |
|  | Model 4 | 0.20 | **(0.10, 0.30)** | 0.01 | (-0.04, 0.06) | Ref (0.00) | 0.14 | **(0.10, 0.19)** | 0.30 | **(0.22, 0.37)** | < 0.001 | 0.324 |
| Allostatic load |  |  |  |  |  |  |  |  |  |  |  |  |
|  | Model 1 | 0.03 | **(0.02, 0.03)** | 0.01 | **(0.01, 0.01)** | Ref (0.00) | 0.01 | (0.00, 0.01) | 0.02 | **(0.02, 0.03)** | < 0.001 | 0.429 |
|  | Model 2 | 0.01 | **(0.01, 0.01)** | 0.00 | (0.00, 0.01) | Ref (0.00) | 0.00 | (0.00, 0.01) | 0.01 | **(0.01, 0.02)** | < 0.001 | 0.169 |
|  | Model 3 | 0.00 | (0.00, 0.01) | 0.00 | (0.00, 0.00) | Ref (0.00) | 0.00 | (0.00, 0.01) | 0.01 | **(0.01, 0.01)** | < 0.001 | 0.139 |
|  | Model 4 | 0.00 | (0.00, 0.01) | 0.00 | (0.00, 0.00) | Ref (0.00) | 0.00 | (0.00, 0.01) | 0.01 | **(0.01, 0.01)** | < 0.001 | 0.142 |

^a^Model 1 was adjusted for age, sex, ethnicity. Model 2 further adjusted for BMI, smoking, drinking, regular exercise, education, Townsend deprivation index, diet score. Model 3 further adjusted for self-reported diabetes, hypertension, CVD, cancer, medication for cholesterol, blood pressure or diabetes, family history of diabetes, hypertension, CVD, cancer. Model 4 further adjusted for sleep disorders, depression and shift work.

^b^Data were listed as beta estimates and 95% confidence intervals, with the bold font lower than the adjusted critical level of significance using Bonferroni’s correction.

^c^Ref, reference.

^d^Tests for trends based on the variables containing the median values for each group.

**Supplementary table 16.** Associations between sleep duration and predicted age metrics among participants without sleep disorder at baseline

|  |  | Habitual sleep duration, h/day | | | | | | | | |  |  |
| --- | --- | --- | --- | --- | --- | --- | --- | --- | --- | --- | --- | --- |
|  |  | ≤ 5 (n = 10,641) | | 6 (n = 43,154) | | 7 (n = 95,469) | 8 (n = 73,640) | | ≥ 9 (n = 18,809) | | *P*_trend_ | *P*_interaction_ |
|  |  | β | 95 CI | β | 95 CI | β | β | 95 CI | β | 95 CI |  |  |
| Homeostatic dysregulation |  |  |  |  |  |  |  |  |  |  |  |  |
|  | Model 1 | 0.14 | **(0.12, 0.16)** | 0.04 | **(0.03, 0.05)** | Ref (0.00) | 0.02 | **(0.01, 0.03)** | 0.09 | **(0.08, 0.11)** | < 0.001 | 0.364 |
|  | Model 2 | 0.09 | **(0.07, 0.11)** | 0.01 | (0.00, 0.02) | Ref (0.00) | 0.02 | **(0.01, 0.03)** | 0.06 | **(0.05, 0.08)** | < 0.001 | 0.308 |
|  | Model 3 | 0.04 | **(0.02, 0.06)** | 0.00 | (-0.01, 0.01) | Ref (0.00) | 0.01 | **(0.01, 0.02)** | 0.03 | **(0.01, 0.04)** | < 0.001 | 0.624 |
|  | Model 4 | 0.05 | **(0.03, 0.07)** | 0.00 | (-0.01, 0.01) | Ref (0.00) | 0.01 | **(0.01, 0.02)** | 0.03 | **(0.02, 0.04)** | < 0.001 | 0.595 |
| PA residual |  |  |  |  |  |  |  |  |  |  |  |  |
|  | Model 1 | 0.80 | **(0.73, 0.88)** | 0.28 | **(0.23, 0.32)** | Ref (0.00) | 0.17 | **(0.14, 0.21)** | 0.81 | **(0.76, 0.87)** | < 0.001 | 0.003 |
|  | Model 2 | 0.29 | **(0.22, 0.36)** | 0.04 | (0.00, 0.07) | Ref (0.00) | 0.15 | **(0.12, 0.18)** | 0.51 | **(0.45, 0.56)** | < 0.001 | 0.211 |
|  | Model 3 | 0.06 | (-0.01, 0.13) | -0.03 | (-0.07, 0.01) | Ref (0.00) | 0.14 | **(0.11, 0.17)** | 0.34 | **(0.29, 0.39)** | < 0.001 | 0.547 |
|  | Model 4 | 0.08 | **(0.01, 0.14)** | -0.03 | (-0.06, 0.01) | Ref (0.00) | 0.14 | **(0.11, 0.17)** | 0.35 | **(0.30, 0.40)** | < 0.001 | 0.528 |
| KDM residual |  |  |  |  |  |  |  |  |  |  |  |  |
|  | Model 1 | 0.78 | **(0.68, 0.88)** | 0.28 | **(0.22, 0.33)** | Ref (0.00) | 0.18 | **(0.14, 0.23)** | 0.65 | **(0.57, 0.72)** | < 0.001 | 0.024 |
|  | Model 2 | 0.32 | **(0.23, 0.42)** | 0.05 | (0.00, 0.10) | Ref (0.00) | 0.15 | **(0.11, 0.20)** | 0.36 | **(0.29, 0.43)** | < 0.001 | 0.272 |
|  | Model 3 | 0.17 | **(0.07, 0.26)** | 0.01 | (-0.04, 0.06) | Ref (0.00) | 0.15 | **(0.10, 0.19)** | 0.26 | **(0.19, 0.33)** | < 0.001 | 0.496 |
|  | Model 4 | 0.20 | **(0.10, 0.29)** | 0.02 | (-0.03, 0.07) | Ref (0.00) | 0.15 | **(0.11, 0.19)** | 0.28 | **(0.21, 0.35)** | < 0.001 | 0.470 |
| Allostatic load |  |  |  |  |  |  |  |  |  |  |  |  |
|  | Model 1 | 0.03 | **(0.03, 0.03)** | 0.01 | **(0.01, 0.02)** | Ref (0.00) | 0.01 | (0.00, 0.01) | 0.03 | **(0.02, 0.03)** | < 0.001 | 0.004 |
|  | Model 2 | 0.01 | **(0.01, 0.01)** | 0.00 | (0.00, 0.01) | Ref (0.00) | 0.00 | (0.00, 0.01) | 0.01 | **(0.01, 0.02)** | < 0.001 | 0.284 |
|  | Model 3 | 0.01 | (0.00, 0.01) | 0.00 | (0.00, 0.00) | Ref (0.00) | 0.00 | (0.00, 0.01) | 0.01 | **(0.01, 0.01)** | < 0.001 | 0.491 |
|  | Model 4 | 0.01 | (0.00, 0.01) | 0.00 | (0.00, 0.00) | Ref (0.00) | 0.00 | (0.00, 0.01) | 0.01 | **(0.01, 0.01)** | < 0.001 | 0.482 |

^a^Model 1 was adjusted for age, sex, ethnicity. Model 2 further adjusted for BMI, smoking, drinking, regular exercise, education, Townsend deprivation index, diet score. Model 3 further adjusted for self-reported diabetes, hypertension, CVD, cancer, medication for cholesterol, blood pressure or diabetes, family history of diabetes, hypertension, CVD, cancer. Model 4 further adjusted for sleep disorders, depression and shift work.

^b^Data were listed as beta estimates and 95% confidence intervals, with the bold font lower than the adjusted critical level of significance using Bonferroni’s correction.

^c^Ref, reference.

^d^Tests for trends based on the variables containing the median values for each group.

**Supplementary table 17.** Associations between sleep duration and predicted age metrics among participants who did not self-report poor health at baseline

|  |  | Habitual sleep duration, h/day | | | | | | | | |  |  |
| --- | --- | --- | --- | --- | --- | --- | --- | --- | --- | --- | --- | --- |
|  |  | ≤ 5 (n = 10,641) | | 6 (n = 43,154) | | 7 (n = 95,469) | 8 (n = 73,640) | | ≥ 9 (n = 18,809) | | *P*_trend_ | *P*_interaction_ |
|  |  | β | 95 CI | β | 95 CI | β | β | 95 CI | β | 95 CI |  |  |
| Homeostatic dysregulation |  |  |  |  |  |  |  |  |  |  |  |  |
|  | Model 1 | 0.12 | **(0.10, 0.13)** | 0.03 | **(0.02, 0.04)** | Ref (0.00) | 0.02 | **(0.01, 0.03)** | 0.07 | **(0.05, 0.08)** | < 0.001 | 0.568 |
|  | Model 2 | 0.07 | **(0.05, 0.09)** | 0.01 | (0.00, 0.02) | Ref (0.00) | 0.01 | **(0.01, 0.02)** | 0.04 | **(0.02, 0.05)** | < 0.001 | 0.948 |
|  | Model 3 | 0.04 | **(0.02, 0.06)** | 0.00 | (-0.01, 0.01) | Ref (0.00) | 0.01 | (0.00, 0.02) | 0.02 | (0.00, 0.03) | 0.007 | 0.902 |
|  | Model 4 | 0.05 | **(0.03, 0.07)** | 0.01 | (-0.01, 0.02) | Ref (0.00) | 0.01 | (0.00, 0.02) | 0.02 | **(0.01, 0.03)** | 0.004 | 0.866 |
| PA residual |  |  |  |  |  |  |  |  |  |  |  |  |
|  | Model 1 | 0.63 | **(0.56, 0.71)** | 0.23 | **(0.19, 0.27)** | Ref (0.00) | 0.16 | **(0.13, 0.20)** | 0.70 | **(0.64, 0.76)** | < 0.001 | 0.357 |
|  | Model 2 | 0.19 | **(0.12, 0.26)** | 0.01 | (-0.03, 0.05) | Ref (0.00) | 0.14 | **(0.11, 0.17)** | 0.43 | **(0.38, 0.49)** | < 0.001 | 0.738 |
|  | Model 3 | 0.07 | (0.00, 0.14) | -0.02 | (-0.05, 0.02) | Ref (0.00) | 0.13 | **(0.10, 0.16)** | 0.34 | **(0.29, 0.39)** | < 0.001 | 0.839 |
|  | Model 4 | 0.09 | **(0.02, 0.16)** | -0.01 | (-0.05, 0.03) | Ref (0.00) | 0.13 | **(0.10, 0.16)** | 0.35 | **(0.29, 0.40)** | < 0.001 | 0.877 |
| KDM residual |  |  |  |  |  |  |  |  |  |  |  |  |
|  | Model 1 | 0.67 | **(0.57, 0.78)** | 0.24 | **(0.18, 0.29)** | Ref (0.00) | 0.17 | **(0.12, 0.21)** | 0.56 | **(0.48, 0.64)** | < 0.001 | 0.029 |
|  | Model 2 | 0.25 | **(0.16, 0.35)** | 0.03 | (-0.03, 0.08) | Ref (0.00) | 0.14 | **(0.10, 0.19)** | 0.31 | **(0.23, 0.38)** | < 0.001 | 0.179 |
|  | Model 3 | 0.14 | **(0.04, 0.23)** | 0.00 | (-0.05, 0.05) | Ref (0.00) | 0.14 | **(0.09, 0.18)** | 0.24 | **(0.16, 0.31)** | < 0.001 | 0.180 |
|  | Model 4 | 0.19 | **(0.10, 0.29)** | 0.01 | (-0.04, 0.06) | Ref (0.00) | 0.14 | **(0.10, 0.18)** | 0.26 | **(0.19, 0.33)** | < 0.001 | 0.153 |
| Allostatic load |  |  |  |  |  |  |  |  |  |  |  |  |
|  | Model 1 | 0.03 | **(0.02, 0.03)** | 0.01 | **(0.01, 0.01)** | Ref (0.00) | 0.01 | (0.00, 0.01) | 0.02 | **(0.02, 0.03)** | < 0.001 | 0.332 |
|  | Model 2 | 0.01 | **(0.01, 0.01)** | 0.00 | (0.00, 0.00) | Ref (0.00) | 0.00 | (0.00, 0.01) | 0.01 | **(0.01, 0.01)** | < 0.001 | 0.808 |
|  | Model 3 | 0.01 | (0.00, 0.01) | 0.00 | (0.00, 0.00) | Ref (0.00) | 0.00 | (0.00, 0.01) | 0.01 | **(0.01, 0.01)** | < 0.001 | 0.831 |
|  | Model 4 | 0.01 | (0.00, 0.01) | 0.00 | (0.00, 0.00) | Ref (0.00) | 0.00 | (0.00, 0.01) | 0.01 | **(0.01, 0.01)** | < 0.001 | 0.842 |

^a^Model 1 was adjusted for age, sex, ethnicity. Model 2 further adjusted for BMI, smoking, drinking, regular exercise, education, Townsend deprivation index, diet score. Model 3 further adjusted for self-reported diabetes, hypertension, CVD, cancer, medication for cholesterol, blood pressure or diabetes, family history of diabetes, hypertension, CVD, cancer. Model 4 further adjusted for sleep disorders, depression and shift work.

^b^Data were listed as beta estimates and 95% confidence intervals, with the bold font lower than the adjusted critical level of significance using Bonferroni’s correction.

^c^Ref, reference.

^d^Tests for trends based on the variables containing the median values for each group.

**Supplementary table 18.** Stratified analysis of the associations between sleep duration and homeostatic dysregulation (HD)

|  |  |  | Habitual sleep duration, h/day | | | | | | | | |  |  |
| --- | --- | --- | --- | --- | --- | --- | --- | --- | --- | --- | --- | --- | --- |
|  |  |  | ≤ 5 (n = 10,641) | | 6 (n = 43,154) | | 7 (n = 95,469) | 8 (n = 73,640) | | ≥ 9 (n = 18,809) | | *P*_trend_ | *P*_interaction_ |
|  |  |  | β | 95 CI | β | 95 CI | β | β | 95 CI | β | 95 CI |  |  |
| Age |  |  |  |  |  |  |  |  |  |  |  |  |  |
|  | Model 1 | ＞ 60 | 0.10 | **(0.07, 0.14)** | 0.00 | (-0.02, 0.02) | Ref (0.00) | 0.03 | **(0.02, 0.05)** | 0.10 | **(0.08, 0.12)** | < 0.001 | < 0.001 |
|  |  | ≤ 60 | 0.21 | **(0.19, 0.23)** | 0.07 | **(0.06, 0.09)** | Ref (0.00) | 0.04 | **(0.03, 0.05)** | 0.17 | **(0.15, 0.19)** | < 0.001 |  |
|  | Model 2 | ＞ 60 | 0.07 | **(0.04, 0.10)** | -0.01 | (-0.03, 0.01) | Ref (0.00) | 0.03 | **(0.01, 0.04)** | 0.07 | **(0.05, 0.10)** | < 0.001 | < 0.001 |
|  |  | ≤ 60 | 0.13 | **(0.11, 0.15)** | 0.03 | **(0.02, 0.05)** | Ref (0.00) | 0.03 | **(0.02, 0.04)** | 0.12 | **(0.10, 0.14)** | < 0.001 |  |
|  | Model 3 | ＞ 60 | 0.04 | **(0.01, 0.07)** | -0.02 | (-0.04, 0.00) | Ref (0.00) | 0.03 | **(0.01, 0.04)** | 0.05 | **(0.03, 0.07)** | < 0.001 | < 0.001 |
|  |  | ≤ 60 | 0.05 | **(0.03, 0.07)** | 0.01 | (0.00, 0.02) | Ref (0.00) | 0.02 | **(0.01, 0.03)** | 0.06 | **(0.04, 0.08)** | < 0.001 |  |
|  | Model 4 | ＞ 60 | 0.04 | **(0.01, 0.08)** | -0.02 | (-0.04, 0.00) | Ref (0.00) | 0.03 | **(0.01, 0.04)** | 0.05 | **(0.03, 0.07)** | < 0.001 | < 0.001 |
|  |  | ≤ 60 | 0.06 | **(0.04, 0.08)** | 0.02 | (0.00, 0.03) | Ref (0.00) | 0.02 | **(0.01, 0.03)** | 0.07 | **(0.05, 0.08)** | < 0.001 |  |
| Sex |  |  |  |  |  |  |  |  |  |  |  |  |  |
|  | Model 1 | Male | 0.16 | **(0.13, 0.19)** | 0.03 | **(0.01, 0.04)** | Ref (0.00) | 0.04 | **(0.02, 0.05)** | 0.14 | **(0.11, 0.16)** | < 0.001 | 0.501 |
|  |  | Female | 0.13 | **(0.11, 0.15)** | 0.04 | **(0.02, 0.05)** | Ref (0.00) | 0.02 | **(0.01, 0.04)** | 0.10 | **(0.08, 0.12)** | < 0.001 |  |
|  | Model 2 | Male | 0.09 | **(0.07, 0.12)** | -0.01 | (-0.02, 0.01) | Ref (0.00) | 0.03 | **(0.02, 0.05)** | 0.10 | **(0.08, 0.12)** | < 0.001 | 0.804 |
|  |  | Female | 0.08 | **(0.05, 0.10)** | 0.02 | (0.00, 0.03) | Ref (0.00) | 0.02 | **(0.01, 0.03)** | 0.06 | **(0.04, 0.08)** | < 0.001 |  |
|  | Model 3 | Male | 0.04 | **(0.01, 0.06)** | -0.02 | **(-0.03, -0.01)** | Ref (0.00) | 0.03 | **(0.02, 0.04)** | 0.05 | **(0.03, 0.07)** | < 0.001 | 0.318 |
|  |  | Female | 0.03 | **(0.01, 0.05)** | 0.00 | (-0.01, 0.02) | Ref (0.00) | 0.02 | **(0.01, 0.03)** | 0.04 | **(0.02, 0.06)** | < 0.001 |  |
|  | Model 4 | Male | 0.05 | **(0.02, 0.07)** | -0.02 | (-0.03, 0.00) | Ref (0.00) | 0.03 | **(0.02, 0.04)** | 0.06 | **(0.04, 0.08)** | < 0.001 | 0.276 |
|  |  | Female | 0.04 | **(0.01, 0.06)** | 0.01 | (-0.01, 0.02) | Ref (0.00) | 0.02 | **(0.01, 0.03)** | 0.05 | **(0.03, 0.06)** | < 0.001 |  |
| Body mass index |  |  |  |  |  |  |  |  |  |  |  |  |  |
|  | Model 1 | ＞ 30 | 0.16 | **(0.12, 0.19)** | 0.02 | (0.00, 0.04) | Ref (0.00) | 0.02 | (0.00, 0.04) | 0.09 | **(0.06, 0.12)** | 0.001 | 0.026 |
|  |  | ≤ 30 | 0.10 | **(0.08, 0.13)** | 0.03 | **(0.01, 0.04)** | Ref (0.00) | 0.02 | **(0.01, 0.03)** | 0.09 | **(0.07, 0.10)** | < 0.001 |  |
|  | Model 2 | ＞ 30 | 0.14 | **(0.10, 0.17)** | 0.01 | (-0.01, 0.04) | Ref (0.00) | 0.02 | (0.00, 0.04) | 0.07 | **(0.04, 0.10)** | 0.007 | 0.016 |
|  |  | ≤ 30 | 0.08 | **(0.06, 0.11)** | 0.02 | **(0.01, 0.03)** | Ref (0.00) | 0.02 | **(0.01, 0.03)** | 0.07 | **(0.06, 0.09)** | < 0.001 |  |
|  | Model 3 | ＞ 30 | 0.06 | **(0.03, 0.09)** | -0.01 | (-0.03, 0.01) | Ref (0.00) | 0.01 | (-0.01, 0.03) | 0.01 | (-0.02, 0.04) | 0.497 | 0.009 |
|  |  | ≤ 30 | 0.03 | **(0.01, 0.06)** | 0.00 | (-0.01, 0.02) | Ref (0.00) | 0.02 | **(0.01, 0.03)** | 0.04 | **(0.02, 0.05)** | < 0.001 |  |
|  | Model 4 | ＞ 30 | 0.07 | **(0.04, 0.11)** | -0.01 | (-0.03, 0.02) | Ref (0.00) | 0.01 | (-0.01, 0.03) | 0.02 | (-0.01, 0.05) | 0.486 | 0.010 |
|  |  | ≤ 30 | 0.04 | **(0.02, 0.06)** | 0.01 | (-0.01, 0.02) | Ref (0.00) | 0.02 | **(0.01, 0.03)** | 0.04 | **(0.03, 0.06)** | < 0.001 |  |
| Smoking |  |  |  |  |  |  |  |  |  |  |  |  |  |
|  | Model 1 | Yes | 0.20 | **(0.15, 0.25)** | 0.05 | **(0.02, 0.08)** | Ref (0.00) | 0.02 | (-0.01, 0.05) | 0.12 | **(0.08, 0.17)** | 0.001 | 0.080 |
|  |  | No | 0.14 | **(0.12, 0.16)** | 0.04 | **(0.02, 0.05)** | Ref (0.00) | 0.02 | **(0.01, 0.03)** | 0.10 | **(0.08, 0.11)** | < 0.001 |  |
|  | Model 2 | Yes | 0.14 | **(0.09, 0.19)** | 0.02 | (-0.01, 0.06) | Ref (0.00) | 0.02 | (-0.01, 0.05) | 0.10 | **(0.05, 0.14)** | 0.008 | 0.208 |
|  |  | No | 0.08 | **(0.06, 0.10)** | 0.01 | (0.00, 0.02) | Ref (0.00) | 0.02 | **(0.01, 0.03)** | 0.06 | **(0.04, 0.08)** | < 0.001 |  |
|  | Model 3 | Yes | 0.06 | **(0.01, 0.11)** | 0.00 | (-0.03, 0.03) | Ref (0.00) | 0.01 | (-0.02, 0.04) | 0.05 | **(0.01, 0.09)** | 0.165 | 0.494 |
|  |  | No | 0.04 | **(0.02, 0.06)** | 0.00 | (-0.01, 0.01) | Ref (0.00) | 0.01 | (0.00, 0.02) | 0.03 | **(0.01, 0.04)** | 0.001 |  |
|  | Model 4 | Yes | 0.07 | **(0.02, 0.11)** | 0.00 | (-0.03, 0.03) | Ref (0.00) | 0.01 | (-0.02, 0.04) | 0.05 | **(0.01, 0.10)** | 0.131 | 0.489 |
|  |  | No | 0.05 | **(0.03, 0.06)** | 0.00 | (-0.01, 0.01) | Ref (0.00) | 0.01 | (0.00, 0.02) | 0.03 | **(0.01, 0.04)** | 0.001 |  |
| Drinking |  |  |  |  |  |  |  |  |  |  |  |  |  |
|  | Model 1 | Yes | 0.15 | **(0.13, 0.17)** | 0.04 | **(0.03, 0.05)** | Ref (0.00) | 0.02 | **(0.01, 0.03)** | 0.09 | **(0.08, 0.11)** | < 0.001 | 0.062 |
|  |  | No | 0.20 | **(0.13, 0.26)** | 0.08 | **(0.03, 0.13)** | Ref (0.00) | 0.07 | **(0.03, 0.11)** | 0.19 | **(0.14, 0.25)** | < 0.001 |  |
|  | Model 2 | Yes | 0.09 | **(0.07, 0.11)** | 0.01 | (0.00, 0.02) | Ref (0.00) | 0.01 | **(0.01, 0.02)** | 0.06 | **(0.05, 0.07)** | < 0.001 | 0.067 |
|  |  | No | 0.10 | **(0.04, 0.17)** | 0.04 | (-0.01, 0.08) | Ref (0.00) | 0.06 | **(0.02, 0.10)** | 0.13 | **(0.08, 0.19)** | < 0.001 |  |
|  | Model 3 | Yes | 0.05 | **(0.03, 0.06)** | 0.00 | (-0.01, 0.01) | Ref (0.00) | 0.01 | (0.00, 0.02) | 0.03 | **(0.01, 0.04)** | 0.002 | 0.055 |
|  |  | No | 0.02 | (-0.04, 0.08) | 0.01 | (-0.04, 0.05) | Ref (0.00) | 0.04 | (0.00, 0.08) | 0.07 | **(0.02, 0.12)** | 0.007 |  |
|  | Model 4 | Yes | 0.05 | **(0.04, 0.07)** | 0.00 | (-0.01, 0.01) | Ref (0.00) | 0.01 | (0.00, 0.02) | 0.03 | **(0.01, 0.04)** | 0.001 | 0.046 |
|  |  | No | 0.03 | (-0.03, 0.09) | 0.01 | (-0.04, 0.05) | Ref (0.00) | 0.04 | (0.00, 0.08) | 0.07 | **(0.02, 0.13)** | 0.007 |  |
| Exercise |  |  |  |  |  |  |  |  |  |  |  |  |  |
|  | Model 1 | High | 0.13 | **(0.11, 0.16)** | 0.02 | **(0.01, 0.04)** | Ref (0.00) | 0.01 | (-0.01, 0.02) | 0.06 | **(0.04, 0.09)** | 0.006 | 0.002 |
|  |  | Moderate | 0.13 | **(0.10, 0.16)** | 0.04 | **(0.02, 0.06)** | Ref (0.00) | 0.03 | **(0.01, 0.04)** | 0.09 | **(0.07, 0.12)** | < 0.001 |  |
|  |  | Low | 0.21 | **(0.17, 0.25)** | 0.07 | **(0.04, 0.09)** | Ref (0.00) | 0.04 | **(0.02, 0.07)** | 0.18 | **(0.14, 0.21)** | < 0.001 |  |
|  | Model 2 | High | 0.09 | **(0.06, 0.12)** | 0.00 | (-0.01, 0.02) | Ref (0.00) | 0.00 | (-0.01, 0.02) | 0.04 | **(0.02, 0.06)** | 0.186 | 0.008 |
|  |  | Moderate | 0.07 | **(0.04, 0.10)** | 0.01 | (0.00, 0.03) | Ref (0.00) | 0.02 | **(0.01, 0.03)** | 0.06 | **(0.04, 0.08)** | < 0.001 |  |
|  |  | Low | 0.12 | **(0.08, 0.17)** | 0.03 | (0.00, 0.05) | Ref (0.00) | 0.04 | **(0.01, 0.06)** | 0.12 | **(0.09, 0.16)** | < 0.001 |  |
|  | Model 3 | High | 0.05 | **(0.03, 0.08)** | -0.01 | (-0.02, 0.01) | Ref (0.00) | 0.00 | (-0.01, 0.02) | 0.02 | (-0.01, 0.04) | 0.547 | 0.098 |
|  |  | Moderate | 0.03 | (0.00, 0.06) | 0.00 | (-0.01, 0.02) | Ref (0.00) | 0.02 | (0.00, 0.03) | 0.03 | (0.00, 0.05) | 0.005 |  |
|  |  | Low | 0.04 | (0.00, 0.09) | 0.01 | (-0.02, 0.03) | Ref (0.00) | 0.03 | (0.00, 0.05) | 0.05 | **(0.02, 0.09)** | 0.003 |  |
|  | Model 4 | High | 0.06 | **(0.03, 0.09)** | -0.01 | (-0.02, 0.01) | Ref (0.00) | 0.00 | (-0.01, 0.02) | 0.02 | (0.00, 0.04) | 0.515 | 0.063 |
|  |  | Moderate | 0.04 | **(0.01, 0.07)** | 0.01 | (-0.01, 0.02) | Ref (0.00) | 0.02 | (0.00, 0.03) | 0.03 | **(0.01, 0.05)** | 0.003 |  |
|  |  | Low | 0.05 | **(0.01, 0.10)** | 0.01 | (-0.02, 0.03) | Ref (0.00) | 0.03 | **(0.01, 0.05)** | 0.06 | **(0.03, 0.09)** | 0.002 |  |
| Education |  |  |  |  |  |  |  |  |  |  |  |  |  |
|  | Model 1 | Above high school | 0.14 | **(0.12, 0.17)** | 0.04 | **(0.02, 0.05)** | Ref (0.00) | 0.02 | **(0.01, 0.03)** | 0.10 | **(0.08, 0.11)** | < 0.001 | 0.224 |
|  |  | High school and below | 0.13 | **(0.10, 0.16)** | 0.03 | **(0.01, 0.05)** | Ref (0.00) | 0.02 | **(0.01, 0.04)** | 0.09 | **(0.07, 0.11)** | < 0.001 |  |
|  | Model 2 | Above high school | 0.09 | **(0.07, 0.12)** | 0.01 | (0.00, 0.03) | Ref (0.00) | 0.02 | **(0.01, 0.03)** | 0.07 | **(0.05, 0.09)** | < 0.001 | 0.669 |
|  |  | High school and below | 0.09 | **(0.06, 0.12)** | 0.01 | (-0.01, 0.03) | Ref (0.00) | 0.02 | **(0.01, 0.04)** | 0.07 | **(0.04, 0.09)** | < 0.001 |  |
|  | Model 3 | Above high school | 0.04 | **(0.02, 0.06)** | 0.00 | (-0.01, 0.01) | Ref (0.00) | 0.01 | (0.00, 0.02) | 0.03 | **(0.01, 0.05)** | 0.005 | 0.455 |
|  |  | High school and below | 0.05 | **(0.02, 0.07)** | 0.00 | (-0.02, 0.02) | Ref (0.00) | 0.02 | (0.00, 0.03) | 0.03 | **(0.01, 0.05)** | 0.011 |  |
|  | Model 4 | Above high school | 0.05 | **(0.02, 0.07)** | 0.00 | (-0.01, 0.02) | Ref (0.00) | 0.01 | (0.00, 0.02) | 0.03 | **(0.01, 0.05)** | 0.002 | 0.517 |
|  |  | High school and below | 0.05 | **(0.03, 0.08)** | 0.00 | (-0.02, 0.02) | Ref (0.00) | 0.02 | (0.00, 0.03) | 0.04 | **(0.01, 0.06)** | 0.008 |  |
| Townsend deprivation index |  |  |  |  |  |  |  |  |  |  |  |  |  |
|  | Model 1 | ≤ -3.27 | 0.10 | **(0.06, 0.13)** | 0.02 | (0.00, 0.04) | Ref (0.00) | 0.01 | (-0.01, 0.03) | 0.08 | **(0.05, 0.10)** | < 0.001 | 0.053 |
|  |  | -3.26 - -0.99 | 0.12 | **(0.09, 0.15)** | 0.02 | (0.00, 0.04) | Ref (0.00) | 0.02 | (0.00, 0.03) | 0.08 | **(0.06, 0.11)** | < 0.001 |  |
|  |  | ＞ -0.98 | 0.20 | **(0.17, 0.23)** | 0.07 | **(0.05, 0.09)** | Ref (0.00) | 0.04 | **(0.02, 0.05)** | 0.14 | **(0.11, 0.16)** | < 0.001 |  |
|  | Model 2 | ≤ -3.27 | 0.06 | **(0.02, 0.09)** | 0.00 | (-0.02, 0.02) | Ref (0.00) | 0.01 | (-0.01, 0.02) | 0.05 | **(0.03, 0.08)** | 0.021 | 0.087 |
|  |  | -3.26 - -0.99 | 0.08 | **(0.05, 0.11)** | 0.00 | (-0.02, 0.02) | Ref (0.00) | 0.01 | (0.00, 0.03) | 0.06 | **(0.03, 0.08)** | 0.003 |  |
|  |  | ＞ -0.98 | 0.12 | **(0.09, 0.15)** | 0.03 | **(0.02, 0.05)** | Ref (0.00) | 0.03 | **(0.01, 0.04)** | 0.08 | **(0.06, 0.11)** | < 0.001 |  |
|  | Model 3 | ≤ -3.27 | 0.02 | (-0.01, 0.05) | -0.01 | (-0.03, 0.01) | Ref (0.00) | 0.01 | (-0.01, 0.02) | 0.02 | (0.00, 0.05) | 0.301 | 0.278 |
|  |  | -3.26 - -0.99 | 0.03 | (0.00, 0.06) | -0.01 | (-0.03, 0.01) | Ref (0.00) | 0.01 | (0.00, 0.03) | 0.02 | (0.00, 0.05) | 0.136 |  |
|  |  | ＞ -0.98 | 0.06 | **(0.03, 0.09)** | 0.02 | (0.00, 0.03) | Ref (0.00) | 0.03 | **(0.01, 0.04)** | 0.04 | **(0.02, 0.06)** | < 0.001 |  |
|  | Model 4 | ≤ -3.27 | 0.03 | (-0.01, 0.06) | -0.01 | (-0.03, 0.01) | Ref (0.00) | 0.01 | (-0.01, 0.02) | 0.02 | (0.00, 0.05) | 0.265 | 0.267 |
|  |  | -3.26 - -0.99 | 0.04 | **(0.01, 0.07)** | -0.01 | (-0.03, 0.01) | Ref (0.00) | 0.01 | (0.00, 0.03) | 0.03 | (0.00, 0.05) | 0.094 |  |
|  |  | ＞ -0.98 | 0.07 | **(0.04, 0.10)** | 0.02 | (0.00, 0.04) | Ref (0.00) | 0.03 | **(0.01, 0.04)** | 0.04 | **(0.02, 0.07)** | < 0.001 |  |

^a^The variables adjusted in each model were the factors mentioned above except the stratification variables.

^b^Data were listed as beta estimates and 95% confidence intervals, with the bold font lower than the adjusted critical level of significance using Bonferroni’s correction.

^c^Ref, reference.

^d^Tests for trends based on the variables containing the median values for each group.

**Supplementary table 19.** Stratified analysis of the associations between sleep duration and PA residual

|  |  |  | Habitual sleep duration, h/day | | | | | | | | |  |  |
| --- | --- | --- | --- | --- | --- | --- | --- | --- | --- | --- | --- | --- | --- |
|  |  |  | ≤ 5 (n = 10,641) | | 6 (n = 43,154) | | 7 (n = 95,469) | 8 (n = 73,640) | | ≥ 9 (n = 18,809) | | *P*_trend_ | *P*_interaction_ |
|  |  |  | β | 95 CI | β | 95 CI | β | β | 95 CI | β | 95 CI |  |  |
| Age |  |  |  |  |  |  |  |  |  |  |  |  |  |
|  | Model 1 | ＞ 60 | 0.70 | **(0.58, 0.82)** | 0.19 | **(0.11, 0.26)** | Ref (0.00) | 0.22 | **(0.16, 0.28)** | 0.92 | **(0.83, 1.01)** | < 0.001 | < 0.001 |
|  |  | ≤ 60 | 0.96 | **(0.87, 1.05)** | 0.33 | **(0.28, 0.38)** | Ref (0.00) | 0.12 | **(0.08, 0.16)** | 0.73 | **(0.65, 0.80)** | < 0.001 |  |
|  | Model 2 | ＞ 60 | 0.24 | **(0.12, 0.35)** | -0.01 | (-0.08, 0.06) | Ref (0.00) | 0.17 | **(0.12, 0.23)** | 0.58 | **(0.50, 0.66)** | < 0.001 | < 0.001 |
|  |  | ≤ 60 | 0.33 | **(0.25, 0.41)** | 0.04 | (0.00, 0.09) | Ref (0.00) | 0.11 | **(0.07, 0.15)** | 0.37 | **(0.30, 0.44)** | < 0.001 |  |
|  | Model 3 | ＞ 60 | 0.00 | (-0.11, 0.11) | -0.08 | **(-0.14, -0.01)** | Ref (0.00) | 0.16 | **(0.11, 0.22)** | 0.42 | **(0.34, 0.50)** | < 0.001 | < 0.001 |
|  |  | ≤ 60 | 0.07 | (-0.02, 0.15) | -0.03 | (-0.08, 0.01) | Ref (0.00) | 0.09 | **(0.05, 0.13)** | 0.18 | **(0.11, 0.24)** | < 0.001 |  |
|  | Model 4 | ＞ 60 | 0.02 | (-0.09, 0.13) | -0.07 | **(-0.14, -0.01)** | Ref (0.00) | 0.16 | **(0.11, 0.22)** | 0.42 | **(0.35, 0.50)** | < 0.001 | < 0.001 |
|  |  | ≤ 60 | 0.09 | **(0.01, 0.17)** | -0.03 | (-0.08, 0.01) | Ref (0.00) | 0.09 | **(0.05, 0.13)** | 0.19 | **(0.12, 0.26)** | < 0.001 |  |
| Sex |  |  |  |  |  |  |  |  |  |  |  |  |  |
|  | Model 1 | Male | 0.96 | **(0.85, 1.06)** | 0.31 | **(0.25, 0.37)** | Ref (0.00) | 0.17 | **(0.12, 0.22)** | 0.91 | **(0.83, 0.99)** | < 0.001 | 0.587 |
|  |  | Female | 0.81 | **(0.71, 0.91)** | 0.28 | **(0.22, 0.34)** | Ref (0.00) | 0.17 | **(0.12, 0.22)** | 0.80 | **(0.72, 0.87)** | < 0.001 |  |
|  | Model 2 | Male | 0.41 | **(0.31, 0.51)** | 0.05 | (-0.01, 0.10) | Ref (0.00) | 0.16 | **(0.11, 0.21)** | 0.59 | **(0.51, 0.67)** | < 0.001 | 0.005 |
|  |  | Female | 0.24 | **(0.15, 0.33)** | 0.04 | (-0.02, 0.09) | Ref (0.00) | 0.11 | **(0.07, 0.16)** | 0.41 | **(0.33, 0.48)** | < 0.001 |  |
|  | Model 3 | Male | 0.12 | **(0.03, 0.22)** | -0.03 | (-0.08, 0.03) | Ref (0.00) | 0.14 | **(0.09, 0.18)** | 0.38 | **(0.31, 0.46)** | < 0.001 | 0.053 |
|  |  | Female | 0.02 | (-0.07, 0.10) | -0.03 | (-0.08, 0.03) | Ref (0.00) | 0.11 | **(0.07, 0.16)** | 0.27 | **(0.20, 0.33)** | < 0.001 |  |
|  | Model 4 | Male | 0.15 | **(0.05, 0.25)** | -0.02 | (-0.07, 0.03) | Ref (0.00) | 0.14 | **(0.09, 0.18)** | 0.39 | **(0.32, 0.47)** | < 0.001 | 0.061 |
|  |  | Female | 0.04 | (-0.05, 0.13) | -0.02 | (-0.07, 0.03) | Ref (0.00) | 0.11 | **(0.07, 0.16)** | 0.28 | **(0.21, 0.35)** | < 0.001 |  |
| Body mass index |  |  |  |  |  |  |  |  |  |  |  |  |  |
|  | Model 1 | ＞ 30 | 0.84 | **(0.71, 0.98)** | 0.18 | **(0.09, 0.26)** | Ref (0.00) | 0.17 | **(0.09, 0.25)** | 0.79 | **(0.67, 0.90)** | < 0.001 | 0.494 |
|  |  | ≤ 30 | 0.47 | **(0.39, 0.55)** | 0.15 | **(0.10, 0.19)** | Ref (0.00) | 0.17 | **(0.13, 0.21)** | 0.67 | **(0.61, 0.73)** | < 0.001 |  |
|  | Model 2 | ＞ 30 | 0.63 | **(0.50, 0.77)** | 0.10 | **(0.01, 0.18)** | Ref (0.00) | 0.15 | **(0.08, 0.23)** | 0.64 | **(0.53, 0.75)** | < 0.001 | 0.273 |
|  |  | ≤ 30 | 0.30 | **(0.22, 0.38)** | 0.08 | **(0.04, 0.12)** | Ref (0.00) | 0.16 | **(0.12, 0.20)** | 0.56 | **(0.49, 0.62)** | < 0.001 |  |
|  | Model 3 | ＞ 30 | 0.21 | **(0.08, 0.33)** | -0.04 | (-0.12, 0.04) | Ref (0.00) | 0.13 | **(0.05, 0.20)** | 0.32 | **(0.21, 0.42)** | < 0.001 | 0.100 |
|  |  | ≤ 30 | 0.02 | (-0.06, 0.10) | 0.00 | (-0.04, 0.04) | Ref (0.00) | 0.15 | **(0.11, 0.18)** | 0.38 | **(0.32, 0.44)** | < 0.001 |  |
|  | Model 4 | ＞ 30 | 0.23 | **(0.10, 0.36)** | -0.04 | (-0.12, 0.04) | Ref (0.00) | 0.13 | **(0.06, 0.20)** | 0.33 | **(0.23, 0.44)** | < 0.001 | 0.111 |
|  |  | ≤ 30 | 0.04 | (-0.04, 0.12) | 0.00 | (-0.04, 0.05) | Ref (0.00) | 0.15 | **(0.11, 0.18)** | 0.39 | **(0.33, 0.45)** | < 0.001 |  |
| Smoking |  |  |  |  |  |  |  |  |  |  |  |  |  |
|  | Model 1 | Yes | 1.15 | **(0.95, 1.35)** | 0.43 | **(0.30, 0.56)** | Ref (0.00) | 0.25 | **(0.13, 0.37)** | 0.82 | **(0.64, 1.00)** | < 0.001 | < 0.001 |
|  |  | No | 0.75 | **(0.67, 0.83)** | 0.24 | **(0.19, 0.28)** | Ref (0.00) | 0.17 | **(0.13, 0.21)** | 0.84 | **(0.78, 0.90)** | < 0.001 |  |
|  | Model 2 | Yes | 0.61 | **(0.42, 0.81)** | 0.20 | **(0.07, 0.32)** | Ref (0.00) | 0.24 | **(0.12, 0.35)** | 0.59 | **(0.43, 0.76)** | < 0.001 | 0.005 |
|  |  | No | 0.22 | **(0.15, 0.29)** | 0.00 | (-0.04, 0.04) | Ref (0.00) | 0.14 | **(0.11, 0.18)** | 0.50 | **(0.44, 0.55)** | < 0.001 |  |
|  | Model 3 | Yes | 0.20 | **(0.01, 0.38)** | 0.05 | (-0.07, 0.17) | Ref (0.00) | 0.21 | **(0.10, 0.32)** | 0.36 | **(0.19, 0.52)** | < 0.001 | 0.042 |
|  |  | No | 0.00 | (-0.07, 0.07) | -0.06 | **(-0.10, -0.02)** | Ref (0.00) | 0.13 | **(0.10, 0.16)** | 0.33 | **(0.28, 0.39)** | < 0.001 |  |
|  | Model 4 | Yes | 0.23 | **(0.05, 0.42)** | 0.06 | (-0.06, 0.18) | Ref (0.00) | 0.21 | **(0.10, 0.32)** | 0.38 | **(0.22, 0.54)** | < 0.001 | 0.040 |
|  |  | No | 0.03 | (-0.04, 0.10) | -0.05 | **(-0.09, -0.01)** | Ref (0.00) | 0.13 | **(0.10, 0.16)** | 0.35 | **(0.29, 0.40)** | < 0.001 |  |
| Drinking |  |  |  |  |  |  |  |  |  |  |  |  |  |
|  | Model 1 | Yes | 0.79 | **(0.71, 0.86)** | 0.26 | **(0.22, 0.30)** | Ref (0.00) | 0.16 | **(0.13, 0.20)** | 0.82 | **(0.76, 0.88)** | < 0.001 | 0.749 |
|  |  | No | 1.26 | **(1.01, 1.52)** | 0.54 | **(0.36, 0.72)** | Ref (0.00) | 0.38 | **(0.22, 0.54)** | 1.19 | **(0.96, 1.41)** | < 0.001 |  |
|  | Model 2 | Yes | 0.29 | **(0.22, 0.36)** | 0.02 | (-0.02, 0.06) | Ref (0.00) | 0.15 | **(0.11, 0.18)** | 0.51 | **(0.46, 0.56)** | < 0.001 | 0.751 |
|  |  | No | 0.42 | **(0.19, 0.65)** | 0.13 | (-0.03, 0.30) | Ref (0.00) | 0.26 | **(0.11, 0.41)** | 0.65 | **(0.45, 0.86)** | < 0.001 |  |
|  | Model 3 | Yes | 0.05 | (-0.02, 0.12) | -0.04 | **(-0.08, -0.01)** | Ref (0.00) | 0.14 | **(0.11, 0.17)** | 0.34 | **(0.29, 0.40)** | < 0.001 | 0.689 |
|  |  | No | 0.05 | (-0.18, 0.27) | 0.01 | (-0.14, 0.17) | Ref (0.00) | 0.20 | **(0.06, 0.34)** | 0.38 | **(0.19, 0.58)** | < 0.001 |  |
|  | Model 4 | Yes | 0.08 | **(0.01, 0.15)** | -0.04 | (-0.07, 0.00) | Ref (0.00) | 0.14 | **(0.11, 0.17)** | 0.36 | **(0.30, 0.41)** | < 0.001 | 0.730 |
|  |  | No | 0.07 | (-0.15, 0.30) | 0.02 | (-0.14, 0.18) | Ref (0.00) | 0.20 | **(0.06, 0.34)** | 0.41 | **(0.21, 0.60)** | < 0.001 |  |
| Exercise |  |  |  |  |  |  |  |  |  |  |  |  |  |
|  | Model 1 | High | 0.69 | **(0.59, 0.80)** | 0.21 | **(0.15, 0.27)** | Ref (0.00) | 0.15 | **(0.10, 0.21)** | 0.66 | **(0.57, 0.75)** | < 0.001 | 0.065 |
|  |  | Moderate | 0.80 | **(0.68, 0.92)** | 0.26 | **(0.20, 0.33)** | Ref (0.00) | 0.18 | **(0.13, 0.24)** | 0.85 | **(0.76, 0.94)** | < 0.001 |  |
|  |  | Low | 1.22 | **(1.05, 1.39)** | 0.46 | **(0.36, 0.56)** | Ref (0.00) | 0.25 | **(0.16, 0.34)** | 1.14 | **(1.01, 1.28)** | < 0.001 |  |
|  | Model 2 | High | 0.28 | **(0.18, 0.38)** | 0.00 | (-0.05, 0.06) | Ref (0.00) | 0.14 | **(0.09, 0.19)** | 0.42 | **(0.34, 0.50)** | < 0.001 | 0.419 |
|  |  | Moderate | 0.22 | **(0.11, 0.33)** | 0.01 | (-0.05, 0.07) | Ref (0.00) | 0.15 | **(0.10, 0.20)** | 0.53 | **(0.45, 0.62)** | < 0.001 |  |
|  |  | Low | 0.45 | **(0.29, 0.60)** | 0.11 | **(0.01, 0.20)** | Ref (0.00) | 0.18 | **(0.10, 0.26)** | 0.65 | **(0.52, 0.77)** | < 0.001 |  |
|  | Model 3 | High | 0.10 | (0.00, 0.19) | -0.05 | (-0.10, 0.01) | Ref (0.00) | 0.14 | **(0.10, 0.19)** | 0.32 | **(0.24, 0.40)** | < 0.001 | 0.468 |
|  |  | Moderate | -0.03 | (-0.14, 0.08) | -0.05 | (-0.11, 0.00) | Ref (0.00) | 0.13 | **(0.09, 0.18)** | 0.36 | **(0.29, 0.44)** | < 0.001 |  |
|  |  | Low | 0.07 | (-0.08, 0.22) | 0.00 | (-0.09, 0.09) | Ref (0.00) | 0.14 | **(0.06, 0.22)** | 0.32 | **(0.20, 0.44)** | < 0.001 |  |
|  | Model 4 | High | 0.12 | **(0.02, 0.22)** | -0.04 | (-0.10, 0.02) | Ref (0.00) | 0.14 | **(0.10, 0.19)** | 0.33 | **(0.25, 0.41)** | < 0.001 | 0.557 |
|  |  | Moderate | -0.01 | (-0.11, 0.10) | -0.05 | (-0.11, 0.01) | Ref (0.00) | 0.13 | **(0.09, 0.18)** | 0.37 | **(0.29, 0.45)** | < 0.001 |  |
|  |  | Low | 0.11 | (-0.04, 0.26) | 0.01 | (-0.08, 0.10) | Ref (0.00) | 0.14 | **(0.07, 0.22)** | 0.35 | **(0.23, 0.47)** | < 0.001 |  |
| Education |  |  |  |  |  |  |  |  |  |  |  |  |  |
|  | Model 1 | Above high school | 0.86 | **(0.76, 0.96)** | 0.27 | **(0.22, 0.32)** | Ref (0.00) | 0.15 | **(0.11, 0.19)** | 0.75 | **(0.68, 0.83)** | < 0.001 | < 0.001 |
|  |  | High school and below | 0.68 | **(0.58, 0.79)** | 0.22 | **(0.15, 0.29)** | Ref (0.00) | 0.18 | **(0.12, 0.24)** | 0.86 | **(0.77, 0.95)** | < 0.001 |  |
|  | Model 2 | Above high school | 0.38 | **(0.29, 0.47)** | 0.04 | (-0.01, 0.08) | Ref (0.00) | 0.15 | **(0.11, 0.19)** | 0.48 | **(0.42, 0.55)** | < 0.001 | 0.030 |
|  |  | High school and below | 0.22 | **(0.12, 0.32)** | 0.01 | (-0.05, 0.08) | Ref (0.00) | 0.16 | **(0.10, 0.21)** | 0.57 | **(0.49, 0.65)** | < 0.001 |  |
|  | Model 3 | Above high school | 0.12 | **(0.03, 0.21)** | -0.03 | (-0.07, 0.02) | Ref (0.00) | 0.14 | **(0.10, 0.18)** | 0.31 | **(0.24, 0.37)** | < 0.001 | 0.010 |
|  |  | High school and below | -0.03 | (-0.12, 0.07) | -0.06 | **(-0.12, 0.00)** | Ref (0.00) | 0.15 | **(0.09, 0.20)** | 0.40 | **(0.32, 0.48)** | < 0.001 |  |
|  | Model 4 | Above high school | 0.14 | **(0.05, 0.23)** | -0.02 | (-0.07, 0.02) | Ref (0.00) | 0.14 | **(0.10, 0.18)** | 0.32 | **(0.25, 0.38)** | < 0.001 | 0.013 |
|  |  | High school and below | 0.01 | (-0.09, 0.10) | -0.05 | (-0.12, 0.01) | Ref (0.00) | 0.15 | **(0.09, 0.20)** | 0.41 | **(0.33, 0.49)** | < 0.001 |  |
| Townsend deprivation index |  |  |  |  |  |  |  |  |  |  |  |  |  |
|  | Model 1 | ≤ -3.27 | 0.45 | **(0.31, 0.58)** | 0.15 | **(0.08, 0.22)** | Ref (0.00) | 0.16 | **(0.10, 0.22)** | 0.69 | **(0.59, 0.79)** | < 0.001 | < 0.001 |
|  |  | -3.26 - -0.99 | 0.58 | **(0.45, 0.71)** | 0.17 | **(0.10, 0.24)** | Ref (0.00) | 0.16 | **(0.10, 0.22)** | 0.73 | **(0.64, 0.83)** | < 0.001 |  |
|  |  | ＞ -0.98 | 1.23 | **(1.11, 1.35)** | 0.48 | **(0.40, 0.55)** | Ref (0.00) | 0.23 | **(0.17, 0.30)** | 1.11 | **(1.01, 1.21)** | < 0.001 |  |
|  | Model 2 | ≤ -3.27 | 0.09 | (-0.03, 0.22) | -0.04 | (-0.10, 0.03) | Ref (0.00) | 0.14 | **(0.09, 0.19)** | 0.46 | **(0.37, 0.55)** | < 0.001 | < 0.001 |
|  |  | -3.26 - -0.99 | 0.18 | **(0.06, 0.31)** | -0.05 | (-0.11, 0.02) | Ref (0.00) | 0.15 | **(0.09, 0.20)** | 0.48 | **(0.39, 0.57)** | < 0.001 |  |
|  |  | ＞ -0.98 | 0.51 | **(0.41, 0.62)** | 0.16 | **(0.09, 0.22)** | Ref (0.00) | 0.16 | **(0.10, 0.22)** | 0.61 | **(0.52, 0.70)** | < 0.001 |  |
|  | Model 3 | ≤ -3.27 | -0.08 | (-0.21, 0.04) | -0.09 | **(-0.15, -0.03)** | Ref (0.00) | 0.13 | **(0.08, 0.18)** | 0.33 | **(0.24, 0.41)** | < 0.001 | < 0.001 |
|  |  | -3.26 - -0.99 | -0.06 | (-0.17, 0.06) | -0.10 | **(-0.16, -0.04)** | Ref (0.00) | 0.13 | **(0.08, 0.18)** | 0.30 | **(0.22, 0.39)** | < 0.001 |  |
|  |  | ＞ -0.98 | 0.20 | **(0.09, 0.30)** | 0.06 | (-0.01, 0.12) | Ref (0.00) | 0.16 | **(0.10, 0.21)** | 0.40 | **(0.31, 0.49)** | < 0.001 |  |
|  | Model 4 | ≤ -3.27 | -0.06 | (-0.18, 0.06) | -0.09 | **(-0.15, -0.02)** | Ref (0.00) | 0.13 | **(0.08, 0.19)** | 0.33 | **(0.25, 0.42)** | < 0.001 | < 0.001 |
|  |  | -3.26 - -0.99 | -0.02 | (-0.14, 0.09) | -0.09 | **(-0.15, -0.03)** | Ref (0.00) | 0.13 | **(0.08, 0.18)** | 0.32 | **(0.23, 0.41)** | < 0.001 |  |
|  |  | ＞ -0.98 | 0.22 | **(0.12, 0.32)** | 0.07 | (0.00, 0.13) | Ref (0.00) | 0.16 | **(0.10, 0.21)** | 0.41 | **(0.32, 0.50)** | < 0.001 |  |

^a^The variables adjusted in each model were the factors mentioned above except the stratification variables.

^b^Data were listed as beta estimates and 95% confidence intervals, with the bold font lower than the adjusted critical level of significance using Bonferroni’s correction.

^c^Ref, reference.

^d^Tests for trends based on the variables containing the median values for each group.

**Supplementary table 20.** Stratified analysis of the associations between sleep duration and KDM residual

|  |  |  | Habitual sleep duration, h/day | | | | | | | | |  |  |
| --- | --- | --- | --- | --- | --- | --- | --- | --- | --- | --- | --- | --- | --- |
|  |  |  | ≤ 5 (n = 10,641) | | 6 (n = 43,154) | | 7 (n = 95,469) | 8 (n = 73,640) | | ≥ 9 (n = 18,809) | | *P*_trend_ | *P*_interaction_ |
|  |  |  | β | 95 CI | β | 95 CI | β | β | 95 CI | β | 95 CI |  |  |
| Age |  |  |  |  |  |  |  |  |  |  |  |  |  |
|  | Model 1 | ＞ 60 | 0.48 | **(0.32, 0.64)** | 0.02 | (-0.08, 0.12) | Ref (0.00) | 0.24 | **(0.16, 0.32)** | 0.67 | **(0.56, 0.78)** | < 0.001 | < 0.001 |
|  |  | ≤ 60 | 1.02 | **(0.90, 1.13)** | 0.41 | **(0.34, 0.47)** | Ref (0.00) | 0.17 | **(0.11, 0.22)** | 0.76 | **(0.66, 0.87)** | < 0.001 |  |
|  | Model 2 | ＞ 60 | 0.17 | **(0.01, 0.33)** | -0.12 | **(-0.21, -0.03)** | Ref (0.00) | 0.19 | **(0.11, 0.27)** | 0.41 | **(0.30, 0.52)** | < 0.001 | < 0.001 |
|  |  | ≤ 60 | 0.38 | **(0.26, 0.49)** | 0.10 | **(0.04, 0.16)** | Ref (0.00) | 0.15 | **(0.09, 0.20)** | 0.40 | **(0.30, 0.49)** | < 0.001 |  |
|  | Model 3 | ＞ 60 | 0.11 | (-0.05, 0.26) | -0.13 | **(-0.23, -0.04)** | Ref (0.00) | 0.20 | **(0.13, 0.28)** | 0.36 | **(0.26, 0.47)** | < 0.001 | < 0.001 |
|  |  | ≤ 60 | 0.12 | **(0.01, 0.22)** | 0.02 | (-0.04, 0.08) | Ref (0.00) | 0.13 | **(0.08, 0.18)** | 0.23 | **(0.14, 0.32)** | < 0.001 |  |
|  | Model 4 | ＞ 60 | 0.16 | (0.00, 0.31) | -0.12 | **(-0.21, -0.03)** | Ref (0.00) | 0.20 | **(0.13, 0.28)** | 0.38 | **(0.27, 0.49)** | < 0.001 | < 0.001 |
|  |  | ≤ 60 | 0.19 | **(0.08, 0.29)** | 0.04 | (-0.03, 0.10) | Ref (0.00) | 0.13 | **(0.08, 0.18)** | 0.27 | **(0.18, 0.36)** | < 0.001 |  |
| Sex |  |  |  |  |  |  |  |  |  |  |  |  |  |
|  | Model 1 | Male | 0.88 | **(0.74, 1.02)** | 0.25 | **(0.17, 0.32)** | Ref (0.00) | 0.21 | **(0.14, 0.28)** | 0.82 | **(0.71, 0.92)** | < 0.001 | 0.626 |
|  |  | Female | 0.71 | **(0.58, 0.84)** | 0.27 | **(0.19, 0.34)** | Ref (0.00) | 0.20 | **(0.14, 0.27)** | 0.65 | **(0.54, 0.75)** | < 0.001 |  |
|  | Model 2 | Male | 0.36 | **(0.23, 0.50)** | -0.01 | (-0.09, 0.06) | Ref (0.00) | 0.20 | **(0.14, 0.27)** | 0.52 | **(0.42, 0.63)** | < 0.001 | 0.155 |
|  |  | Female | 0.23 | **(0.11, 0.36)** | 0.05 | (-0.02, 0.13) | Ref (0.00) | 0.14 | **(0.08, 0.20)** | 0.29 | **(0.20, 0.39)** | < 0.001 |  |
|  | Model 3 | Male | 0.17 | **(0.04, 0.30)** | -0.06 | (-0.13, 0.01) | Ref (0.00) | 0.19 | **(0.13, 0.25)** | 0.37 | **(0.27, 0.47)** | < 0.001 | 0.839 |
|  |  | Female | 0.07 | (-0.05, 0.20) | 0.02 | (-0.05, 0.09) | Ref (0.00) | 0.16 | **(0.10, 0.22)** | 0.28 | **(0.18, 0.37)** | < 0.001 |  |
|  | Model 4 | Male | 0.23 | **(0.09, 0.36)** | -0.05 | (-0.12, 0.02) | Ref (0.00) | 0.19 | **(0.13, 0.25)** | 0.39 | **(0.29, 0.49)** | < 0.001 | 0.908 |
|  |  | Female | 0.13 | **(0.01, 0.25)** | 0.03 | (-0.04, 0.10) | Ref (0.00) | 0.16 | **(0.10, 0.22)** | 0.31 | **(0.21, 0.40)** | < 0.001 |  |
| Body mass index |  |  |  |  |  |  |  |  |  |  |  |  |  |
|  | Model 1 | ＞ 30 | 0.62 | **(0.44, 0.79)** | 0.09 | (-0.02, 0.20) | Ref (0.00) | 0.17 | **(0.07, 0.27)** | 0.38 | **(0.24, 0.53)** | < 0.001 | 0.001 |
|  |  | ≤ 30 | 0.45 | **(0.34, 0.57)** | 0.15 | **(0.08, 0.21)** | Ref (0.00) | 0.18 | **(0.13, 0.23)** | 0.58 | **(0.50, 0.67)** | < 0.001 |  |
|  | Model 2 | ＞ 30 | 0.57 | **(0.40, 0.75)** | 0.07 | (-0.04, 0.18) | Ref (0.00) | 0.16 | **(0.06, 0.26)** | 0.34 | **(0.19, 0.48)** | < 0.001 | 0.001 |
|  |  | ≤ 30 | 0.35 | **(0.24, 0.46)** | 0.10 | **(0.04, 0.16)** | Ref (0.00) | 0.16 | **(0.11, 0.21)** | 0.50 | **(0.42, 0.59)** | < 0.001 |  |
|  | Model 3 | ＞ 30 | 0.31 | **(0.14, 0.48)** | 0.00 | (-0.11, 0.11) | Ref (0.00) | 0.14 | **(0.04, 0.24)** | 0.17 | **(0.03, 0.31)** | 0.011 | 0.001 |
|  |  | ≤ 30 | 0.12 | **(0.01, 0.23)** | 0.04 | (-0.02, 0.09) | Ref (0.00) | 0.16 | **(0.11, 0.20)** | 0.35 | **(0.27, 0.44)** | < 0.001 |  |
|  | Model 4 | ＞ 30 | 0.36 | **(0.19, 0.53)** | 0.01 | (-0.09, 0.12) | Ref (0.00) | 0.14 | **(0.05, 0.24)** | 0.20 | **(0.06, 0.35)** | 0.005 | 0.001 |
|  |  | ≤ 30 | 0.17 | **(0.06, 0.28)** | 0.05 | (-0.01, 0.11) | Ref (0.00) | 0.16 | **(0.11, 0.21)** | 0.38 | **(0.29, 0.46)** | < 0.001 |  |
| Smoking |  |  |  |  |  |  |  |  |  |  |  |  |  |
|  | Model 1 | Yes | 0.91 | **(0.65, 1.17)** | 0.27 | **(0.10, 0.44)** | Ref (0.00) | 0.16 | (0.00, 0.31) | 0.53 | **(0.30, 0.76)** | 0.001 | 0.016 |
|  |  | No | 0.76 | **(0.66, 0.87)** | 0.26 | **(0.20, 0.32)** | Ref (0.00) | 0.18 | **(0.14, 0.23)** | 0.69 | **(0.61, 0.77)** | < 0.001 |  |
|  | Model 2 | Yes | 0.52 | **(0.27, 0.77)** | 0.09 | (-0.07, 0.25) | Ref (0.00) | 0.16 | **(0.01, 0.31)** | 0.38 | **(0.16, 0.61)** | 0.005 | 0.104 |
|  |  | No | 0.25 | **(0.16, 0.35)** | 0.02 | (-0.04, 0.07) | Ref (0.00) | 0.15 | **(0.10, 0.20)** | 0.36 | **(0.29, 0.44)** | < 0.001 |  |
|  | Model 3 | Yes | 0.22 | (-0.03, 0.47) | -0.01 | (-0.16, 0.15) | Ref (0.00) | 0.13 | (-0.02, 0.27) | 0.20 | (-0.02, 0.41) | 0.065 | 0.136 |
|  |  | No | 0.11 | **(0.02, 0.21)** | -0.01 | (-0.07, 0.04) | Ref (0.00) | 0.14 | **(0.10, 0.19)** | 0.27 | **(0.20, 0.34)** | < 0.001 |  |
|  | Model 4 | Yes | 0.29 | **(0.05, 0.54)** | 0.01 | (-0.15, 0.17) | Ref (0.00) | 0.14 | (-0.01, 0.28) | 0.25 | **(0.03, 0.46)** | 0.035 | 0.134 |
|  |  | No | 0.17 | **(0.08, 0.27)** | 0.00 | (-0.05, 0.06) | Ref (0.00) | 0.14 | **(0.10, 0.19)** | 0.30 | **(0.22, 0.37)** | < 0.001 |  |
| Drinking |  |  |  |  |  |  |  |  |  |  |  |  |  |
|  | Model 1 | Yes | 0.80 | **(0.69, 0.90)** | 0.26 | **(0.21, 0.32)** | Ref (0.00) | 0.17 | **(0.12, 0.22)** | 0.67 | **(0.59, 0.75)** | < 0.001 | 0.940 |
|  |  | No | 1.00 | **(0.68, 1.32)** | 0.46 | **(0.23, 0.69)** | Ref (0.00) | 0.38 | **(0.17, 0.58)** | 0.96 | **(0.68, 1.24)** | < 0.001 |  |
|  | Model 2 | Yes | 0.32 | **(0.22, 0.42)** | 0.03 | (-0.02, 0.08) | Ref (0.00) | 0.15 | **(0.10, 0.19)** | 0.37 | **(0.29, 0.44)** | < 0.001 | 0.813 |
|  |  | No | 0.35 | **(0.04, 0.65)** | 0.14 | (-0.08, 0.35) | Ref (0.00) | 0.27 | **(0.08, 0.46)** | 0.50 | **(0.24, 0.77)** | < 0.001 |  |
|  | Model 3 | Yes | 0.16 | **(0.06, 0.25)** | -0.01 | (-0.06, 0.05) | Ref (0.00) | 0.14 | **(0.10, 0.18)** | 0.26 | **(0.19, 0.34)** | < 0.001 | 0.880 |
|  |  | No | 0.15 | (-0.15, 0.45) | 0.04 | (-0.17, 0.26) | Ref (0.00) | 0.23 | **(0.05, 0.42)** | 0.36 | **(0.10, 0.63)** | 0.003 |  |
|  | Model 4 | Yes | 0.22 | **(0.12, 0.31)** | 0.01 | (-0.04, 0.06) | Ref (0.00) | 0.14 | **(0.10, 0.19)** | 0.29 | **(0.22, 0.36)** | < 0.001 | 0.947 |
|  |  | No | 0.20 | (-0.10, 0.50) | 0.05 | (-0.16, 0.27) | Ref (0.00) | 0.24 | **(0.05, 0.43)** | 0.40 | **(0.14, 0.66)** | 0.002 |  |
| Exercise |  |  |  |  |  |  |  |  |  |  |  |  |  |
|  | Model 1 | High | 0.75 | **(0.60, 0.90)** | 0.20 | **(0.11, 0.28)** | Ref (0.00) | 0.14 | **(0.07, 0.21)** | 0.57 | **(0.45, 0.69)** | < 0.001 | 0.416 |
|  |  | Moderate | 0.74 | **(0.58, 0.89)** | 0.28 | **(0.20, 0.37)** | Ref (0.00) | 0.21 | **(0.14, 0.28)** | 0.66 | **(0.54, 0.78)** | < 0.001 |  |
|  |  | Low | 0.96 | **(0.74, 1.18)** | 0.39 | **(0.26, 0.52)** | Ref (0.00) | 0.23 | **(0.11, 0.34)** | 0.87 | **(0.70, 1.04)** | < 0.001 |  |
|  | Model 2 | High | 0.37 | **(0.23, 0.51)** | 0.00 | (-0.08, 0.08) | Ref (0.00) | 0.12 | **(0.06, 0.19)** | 0.33 | **(0.22, 0.44)** | < 0.001 | 0.748 |
|  |  | Moderate | 0.21 | **(0.06, 0.36)** | 0.04 | (-0.04, 0.13) | Ref (0.00) | 0.18 | **(0.11, 0.24)** | 0.37 | **(0.25, 0.48)** | < 0.001 |  |
|  |  | Low | 0.35 | **(0.14, 0.55)** | 0.08 | (-0.04, 0.21) | Ref (0.00) | 0.17 | **(0.06, 0.28)** | 0.46 | **(0.30, 0.63)** | < 0.001 |  |
|  | Model 3 | High | 0.24 | **(0.10, 0.38)** | -0.04 | (-0.11, 0.04) | Ref (0.00) | 0.12 | **(0.06, 0.19)** | 0.27 | **(0.16, 0.38)** | < 0.001 | 0.851 |
|  |  | Moderate | 0.05 | (-0.09, 0.20) | 0.01 | (-0.07, 0.09) | Ref (0.00) | 0.17 | **(0.10, 0.23)** | 0.26 | **(0.15, 0.37)** | < 0.001 |  |
|  |  | Low | 0.09 | (-0.11, 0.30) | 0.03 | (-0.09, 0.15) | Ref (0.00) | 0.13 | **(0.03, 0.24)** | 0.27 | **(0.11, 0.43)** | 0.001 |  |
|  | Model 4 | High | 0.29 | **(0.15, 0.43)** | -0.02 | (-0.10, 0.06) | Ref (0.00) | 0.12 | **(0.06, 0.19)** | 0.29 | **(0.18, 0.40)** | < 0.001 | 0.996 |
|  |  | Moderate | 0.12 | (-0.03, 0.26) | 0.02 | (-0.06, 0.10) | Ref (0.00) | 0.17 | **(0.10, 0.23)** | 0.28 | **(0.17, 0.39)** | < 0.001 |  |
|  |  | Low | 0.16 | (-0.04, 0.36) | 0.04 | (-0.08, 0.16) | Ref (0.00) | 0.14 | **(0.04, 0.25)** | 0.31 | **(0.15, 0.48)** | < 0.001 |  |
| Education |  |  |  |  |  |  |  |  |  |  |  |  |  |
|  | Model 1 | Above high school | 0.87 | **(0.74, 1.00)** | 0.27 | **(0.20, 0.33)** | Ref (0.00) | 0.15 | **(0.09, 0.20)** | 0.63 | **(0.54, 0.73)** | < 0.001 | 0.008 |
|  |  | High school and below | 0.53 | **(0.39, 0.68)** | 0.19 | **(0.10, 0.28)** | Ref (0.00) | 0.19 | **(0.11, 0.27)** | 0.60 | **(0.49, 0.72)** | < 0.001 |  |
|  | Model 2 | Above high school | 0.40 | **(0.28, 0.53)** | 0.03 | (-0.04, 0.09) | Ref (0.00) | 0.15 | **(0.09, 0.20)** | 0.37 | **(0.28, 0.47)** | < 0.001 | 0.281 |
|  |  | High school and below | 0.21 | **(0.07, 0.35)** | 0.04 | (-0.05, 0.13) | Ref (0.00) | 0.17 | **(0.09, 0.24)** | 0.38 | **(0.27, 0.50)** | < 0.001 |  |
|  | Model 3 | Above high school | 0.22 | **(0.09, 0.34)** | -0.01 | (-0.07, 0.05) | Ref (0.00) | 0.14 | **(0.08, 0.19)** | 0.25 | **(0.16, 0.34)** | < 0.001 | 0.101 |
|  |  | High school and below | 0.08 | (-0.06, 0.22) | 0.00 | (-0.09, 0.09) | Ref (0.00) | 0.16 | **(0.09, 0.24)** | 0.31 | **(0.19, 0.42)** | < 0.001 |  |
|  | Model 4 | Above high school | 0.27 | **(0.15, 0.39)** | 0.00 | (-0.06, 0.07) | Ref (0.00) | 0.14 | **(0.09, 0.19)** | 0.28 | **(0.19, 0.37)** | < 0.001 | 0.139 |
|  |  | High school and below | 0.14 | **(0.01, 0.28)** | 0.02 | (-0.07, 0.10) | Ref (0.00) | 0.17 | **(0.09, 0.24)** | 0.33 | **(0.22, 0.45)** | < 0.001 |  |
| Townsend deprivation index |  |  |  |  |  |  |  |  |  |  |  |  |  |
|  | Model 1 | ≤ -3.27 | 0.50 | **(0.31, 0.68)** | 0.17 | **(0.08, 0.27)** | Ref (0.00) | 0.17 | **(0.09, 0.24)** | 0.62 | **(0.49, 0.75)** | < 0.001 | 0.001 |
|  |  | -3.26 - -0.99 | 0.64 | **(0.47, 0.81)** | 0.15 | **(0.06, 0.25)** | Ref (0.00) | 0.13 | **(0.05, 0.21)** | 0.48 | **(0.35, 0.61)** | < 0.001 |  |
|  |  | ＞ -0.98 | 1.10 | **(0.95, 1.25)** | 0.47 | **(0.37, 0.56)** | Ref (0.00) | 0.25 | **(0.17, 0.34)** | 0.93 | **(0.80, 1.06)** | < 0.001 |  |
|  | Model 2 | ≤ -3.27 | 0.11 | (-0.07, 0.28) | -0.03 | (-0.12, 0.06) | Ref (0.00) | 0.14 | **(0.07, 0.22)** | 0.39 | **(0.26, 0.51)** | < 0.001 | 0.002 |
|  |  | -3.26 - -0.99 | 0.25 | **(0.08, 0.41)** | -0.06 | (-0.15, 0.03) | Ref (0.00) | 0.11 | **(0.04, 0.19)** | 0.23 | **(0.10, 0.35)** | 0.001 |  |
|  |  | ＞ -0.98 | 0.45 | **(0.31, 0.60)** | 0.17 | **(0.08, 0.26)** | Ref (0.00) | 0.20 | **(0.12, 0.28)** | 0.48 | **(0.36, 0.61)** | < 0.001 |  |
|  | Model 3 | ≤ -3.27 | -0.01 | (-0.18, 0.16) | -0.06 | (-0.15, 0.03) | Ref (0.00) | 0.14 | **(0.06, 0.21)** | 0.29 | **(0.16, 0.41)** | < 0.001 | 0.004 |
|  |  | -3.26 - -0.99 | 0.09 | (-0.08, 0.25) | -0.08 | (-0.17, 0.01) | Ref (0.00) | 0.11 | **(0.03, 0.18)** | 0.15 | **(0.02, 0.27)** | 0.006 |  |
|  |  | ＞ -0.98 | 0.25 | **(0.11, 0.39)** | 0.10 | **(0.02, 0.19)** | Ref (0.00) | 0.19 | **(0.11, 0.26)** | 0.35 | **(0.23, 0.48)** | < 0.001 |  |
|  | Model 4 | ≤ -3.27 | 0.05 | (-0.12, 0.22) | -0.05 | (-0.14, 0.04) | Ref (0.00) | 0.14 | **(0.07, 0.21)** | 0.31 | **(0.18, 0.43)** | < 0.001 | 0.003 |
|  |  | -3.26 - -0.99 | 0.15 | (-0.01, 0.31) | -0.06 | (-0.15, 0.03) | Ref (0.00) | 0.11 | **(0.04, 0.18)** | 0.17 | **(0.05, 0.30)** | 0.002 |  |
|  |  | ＞ -0.98 | 0.31 | **(0.17, 0.45)** | 0.12 | **(0.03, 0.21)** | Ref (0.00) | 0.19 | **(0.11, 0.27)** | 0.39 | **(0.27, 0.51)** | < 0.001 |  |

^a^The variables adjusted in each model were the factors mentioned above except the stratification variables.

^b^Data were listed as beta estimates and 95% confidence intervals, with the bold font lower than the adjusted critical level of significance using Bonferroni’s correction.

^c^Ref, reference.

^d^Tests for trends based on the variables containing the median values for each group.

**Supplementary table 21.** Stratified analysis of the associations between sleep duration and allostatic load (AL)

|  |  |  | Habitual sleep duration, h/day | | | | | | | | |  |  |
| --- | --- | --- | --- | --- | --- | --- | --- | --- | --- | --- | --- | --- | --- |
|  |  |  | ≤ 5 (n = 10,641) | | 6 (n = 43,154) | | 7 (n = 95,469) | 8 (n = 73,640) | | ≥ 9 (n = 18,809) | | *P*_trend_ | *P*_interaction_ |
|  |  |  | β | 95 CI | β | 95 CI | β | β | 95 CI | β | 95 CI |  |  |
| Age |  |  |  |  |  |  |  |  |  |  |  |  |  |
|  | Model 1 | ＞ 60 | 0.02 | **(0.02, 0.03)** | 0.01 | **(0.01, 0.01)** | Ref (0.00) | 0.01 | **(0.01, 0.01)** | 0.03 | **(0.03, 0.04)** | < 0.001 | < 0.001 |
|  |  | ≤ 60 | 0.04 | **(0.04, 0.05)** | 0.02 | **(0.02, 0.02)** | Ref (0.00) | 0.01 | (0.00, 0.01) | 0.03 | **(0.03, 0.03)** | < 0.001 |  |
|  | Model 2 | ＞ 60 | 0.01 | (0.00, 0.01) | 0.00 | (0.00, 0.00) | Ref (0.00) | 0.01 | **(0.01, 0.01)** | 0.02 | **(0.02, 0.02)** | < 0.001 | < 0.001 |
|  |  | ≤ 60 | 0.02 | **(0.01, 0.02)** | 0.01 | (0.00, 0.01) | Ref (0.00) | 0.01 | (0.00, 0.01) | 0.02 | **(0.01, 0.02)** | < 0.001 |  |
|  | Model 3 | ＞ 60 | 0.00 | (0.00, 0.01) | 0.00 | (0.00, 0.00) | Ref (0.00) | 0.01 | **(0.01, 0.01)** | 0.02 | **(0.01, 0.02)** | < 0.001 | < 0.001 |
|  |  | ≤ 60 | 0.01 | **(0.01, 0.01)** | 0.00 | (0.00, 0.01) | Ref (0.00) | 0.00 | (0.00, 0.01) | 0.01 | **(0.01, 0.01)** | < 0.001 |  |
|  | Model 4 | ＞ 60 | 0.00 | (0.00, 0.01) | 0.00 | (0.00, 0.00) | Ref (0.00) | 0.01 | **(0.01, 0.01)** | 0.02 | **(0.01, 0.02)** | < 0.001 | < 0.001 |
|  |  | ≤ 60 | 0.01 | **(0.01, 0.01)** | 0.00 | (0.00, 0.01) | Ref (0.00) | 0.00 | (0.00, 0.01) | 0.01 | **(0.01, 0.01)** | < 0.001 |  |
| Sex |  |  |  |  |  |  |  |  |  |  |  |  |  |
|  | Model 1 | Male | 0.03 | **(0.02, 0.03)** | 0.01 | **(0.01, 0.01)** | Ref (0.00) | 0.01 | (0.00, 0.01) | 0.03 | **(0.03, 0.04)** | < 0.001 | 0.989 |
|  |  | Female | 0.03 | **(0.03, 0.04)** | 0.02 | **(0.01, 0.02)** | Ref (0.00) | 0.01 | **(0.01, 0.01)** | 0.03 | **(0.02, 0.03)** | < 0.001 |  |
|  | Model 2 | Male | 0.01 | (0.00, 0.01) | 0.00 | (0.00, 0.00) | Ref (0.00) | 0.01 | (0.00, 0.01) | 0.02 | **(0.02, 0.02)** | < 0.001 | 0.048 |
|  |  | Female | 0.01 | **(0.01, 0.02)** | 0.01 | (0.00, 0.01) | Ref (0.00) | 0.01 | (0.00, 0.01) | 0.01 | **(0.01, 0.02)** | < 0.001 |  |
|  | Model 3 | Male | 0.00 | (0.00, 0.01) | 0.00 | (0.00, 0.00) | Ref (0.00) | 0.01 | (0.00, 0.01) | 0.02 | **(0.01, 0.02)** | < 0.001 | 0.175 |
|  |  | Female | 0.01 | (0.00, 0.01) | 0.00 | (0.00, 0.01) | Ref (0.00) | 0.01 | (0.00, 0.01) | 0.01 | **(0.01, 0.01)** | < 0.001 |  |
|  | Model 4 | Male | 0.00 | (0.00, 0.01) | 0.00 | (0.00, 0.00) | Ref (0.00) | 0.01 | (0.00, 0.01) | 0.02 | **(0.01, 0.02)** | < 0.001 | 0.189 |
|  |  | Female | 0.01 | (0.00, 0.01) | 0.00 | (0.00, 0.01) | Ref (0.00) | 0.01 | (0.00, 0.01) | 0.01 | **(0.01, 0.01)** | < 0.001 |  |
| Body mass index |  |  |  |  |  |  |  |  |  |  |  |  |  |
|  | Model 1 | ＞ 30 | 0.03 | **(0.02, 0.03)** | 0.01 | (0.00, 0.01) | Ref (0.00) | 0.01 | (0.00, 0.01) | 0.02 | **(0.02, 0.03)** | < 0.001 | 0.897 |
|  |  | ≤ 30 | 0.02 | **(0.02, 0.02)** | 0.01 | **(0.01, 0.01)** | Ref (0.00) | 0.01 | (0.00, 0.01) | 0.02 | **(0.02, 0.02)** | < 0.001 |  |
|  | Model 2 | ＞ 30 | 0.02 | **(0.02, 0.03)** | 0.01 | (0.00, 0.01) | Ref (0.00) | 0.01 | (0.00, 0.01) | 0.02 | **(0.01, 0.02)** | < 0.001 | 0.584 |
|  |  | ≤ 30 | 0.01 | **(0.01, 0.01)** | 0.01 | (0.00, 0.01) | Ref (0.00) | 0.00 | (0.00, 0.01) | 0.02 | **(0.01, 0.02)** | < 0.001 |  |
|  | Model 3 | ＞ 30 | 0.01 | **(0.01, 0.02)** | 0.00 | (0.00, 0.01) | Ref (0.00) | 0.01 | (0.00, 0.01) | 0.01 | **(0.01, 0.01)** | < 0.001 | 0.455 |
|  |  | ≤ 30 | 0.00 | (0.00, 0.01) | 0.00 | (0.00, 0.01) | Ref (0.00) | 0.00 | (0.00, 0.01) | 0.01 | **(0.01, 0.01)** | < 0.001 |  |
|  | Model 4 | ＞ 30 | 0.01 | **(0.01, 0.02)** | 0.00 | (0.00, 0.01) | Ref (0.00) | 0.01 | (0.00, 0.01) | 0.01 | **(0.01, 0.01)** | < 0.001 | 0.455 |
|  |  | ≤ 30 | 0.00 | (0.00, 0.01) | 0.00 | (0.00, 0.01) | Ref (0.00) | 0.00 | (0.00, 0.01) | 0.01 | **(0.01, 0.01)** | < 0.001 |  |
| Smoking |  |  |  |  |  |  |  |  |  |  |  |  |  |
|  | Model 1 | Yes | 0.04 | **(0.03, 0.05)** | 0.02 | **(0.02, 0.03)** | Ref (0.00) | 0.01 | (0.00, 0.01) | 0.03 | **(0.02, 0.03)** | < 0.001 | < 0.001 |
|  |  | No | 0.03 | **(0.02, 0.03)** | 0.01 | **(0.01, 0.01)** | Ref (0.00) | 0.01 | (0.00, 0.01) | 0.03 | **(0.02, 0.03)** | < 0.001 |  |
|  | Model 2 | Yes | 0.02 | **(0.01, 0.03)** | 0.01 | **(0.01, 0.02)** | Ref (0.00) | 0.01 | (0.00, 0.01) | 0.02 | **(0.01, 0.02)** | < 0.001 | 0.009 |
|  |  | No | 0.01 | (0.00, 0.01) | 0.00 | (0.00, 0.00) | Ref (0.00) | 0.00 | (0.00, 0.01) | 0.01 | **(0.01, 0.02)** | < 0.001 |  |
|  | Model 3 | Yes | 0.01 | (0.00, 0.02) | 0.01 | (0.00, 0.01) | Ref (0.00) | 0.01 | (0.00, 0.01) | 0.01 | (0.00, 0.02) | 0.003 | 0.033 |
|  |  | No | 0.00 | (0.00, 0.01) | 0.00 | (0.00, 0.00) | Ref (0.00) | 0.00 | (0.00, 0.01) | 0.01 | **(0.01, 0.01)** | < 0.001 |  |
|  | Model 4 | Yes | 0.01 | (0.00, 0.02) | 0.01 | (0.00, 0.01) | Ref (0.00) | 0.01 | (0.00, 0.01) | 0.01 | (0.00, 0.02) | 0.002 | 0.035 |
|  |  | No | 0.00 | (0.00, 0.01) | 0.00 | (0.00, 0.00) | Ref (0.00) | 0.00 | (0.00, 0.01) | 0.01 | **(0.01, 0.01)** | < 0.001 |  |
| Drinking |  |  |  |  |  |  |  |  |  |  |  |  |  |
|  | Model 1 | Yes | 0.03 | **(0.03, 0.03)** | 0.01 | **(0.01, 0.01)** | Ref (0.00) | 0.01 | (0.00, 0.01) | 0.03 | **(0.02, 0.03)** | < 0.001 | 0.127 |
|  |  | No | 0.05 | **(0.04, 0.06)** | 0.02 | **(0.02, 0.03)** | Ref (0.00) | 0.01 | **(0.01, 0.02)** | 0.04 | **(0.03, 0.04)** | < 0.001 |  |
|  | Model 2 | Yes | 0.01 | **(0.01, 0.01)** | 0.00 | (0.00, 0.01) | Ref (0.00) | 0.00 | (0.00, 0.01) | 0.01 | **(0.01, 0.02)** | < 0.001 | 0.126 |
|  |  | No | 0.02 | **(0.01, 0.03)** | 0.01 | (0.00, 0.01) | Ref (0.00) | 0.01 | (0.00, 0.01) | 0.02 | **(0.01, 0.02)** | < 0.001 |  |
|  | Model 3 | Yes | 0.00 | (0.00, 0.01) | 0.00 | (0.00, 0.00) | Ref (0.00) | 0.00 | (0.00, 0.01) | 0.01 | **(0.01, 0.01)** | < 0.001 | 0.119 |
|  |  | No | 0.01 | (0.00, 0.02) | 0.00 | (0.00, 0.01) | Ref (0.00) | 0.01 | (0.00, 0.01) | 0.01 | (0.00, 0.02) | 0.008 |  |
|  | Model 4 | Yes | 0.00 | (0.00, 0.01) | 0.00 | (0.00, 0.00) | Ref (0.00) | 0.00 | (0.00, 0.01) | 0.01 | **(0.01, 0.01)** | < 0.001 | 0.127 |
|  |  | No | 0.01 | (0.00, 0.02) | 0.00 | (0.00, 0.01) | Ref (0.00) | 0.01 | (0.00, 0.01) | 0.01 | (0.00, 0.02) | 0.007 |  |
| Exercise |  |  |  |  |  |  |  |  |  |  |  |  |  |
|  | Model 1 | High | 0.03 | **(0.02, 0.03)** | 0.01 | **(0.01, 0.01)** | Ref (0.00) | 0.00 | (0.00, 0.01) | 0.02 | **(0.02, 0.02)** | < 0.001 | 0.014 |
|  |  | Moderate | 0.03 | **(0.03, 0.04)** | 0.02 | **(0.01, 0.02)** | Ref (0.00) | 0.01 | (0.00, 0.01) | 0.03 | **(0.02, 0.03)** | < 0.001 |  |
|  |  | Low | 0.04 | **(0.04, 0.05)** | 0.02 | **(0.02, 0.02)** | Ref (0.00) | 0.01 | **(0.01, 0.01)** | 0.04 | **(0.03, 0.04)** | < 0.001 |  |
|  | Model 2 | High | 0.01 | **(0.01, 0.02)** | 0.00 | (0.00, 0.00) | Ref (0.00) | 0.00 | (0.00, 0.01) | 0.01 | **(0.01, 0.01)** | < 0.001 | 0.118 |
|  |  | Moderate | 0.01 | (0.00, 0.01) | 0.01 | (0.00, 0.01) | Ref (0.00) | 0.01 | (0.00, 0.01) | 0.02 | **(0.01, 0.02)** | < 0.001 |  |
|  |  | Low | 0.02 | **(0.01, 0.02)** | 0.01 | (0.00, 0.01) | Ref (0.00) | 0.01 | (0.00, 0.01) | 0.02 | **(0.02, 0.03)** | < 0.001 |  |
|  | Model 3 | High | 0.01 | (0.00, 0.01) | 0.00 | (0.00, 0.00) | Ref (0.00) | 0.00 | (0.00, 0.01) | 0.01 | (0.00, 0.01) | < 0.001 | 0.509 |
|  |  | Moderate | 0.00 | (0.00, 0.01) | 0.00 | (0.00, 0.01) | Ref (0.00) | 0.00 | (0.00, 0.01) | 0.01 | **(0.01, 0.01)** | < 0.001 |  |
|  |  | Low | 0.01 | (0.00, 0.01) | 0.00 | (0.00, 0.01) | Ref (0.00) | 0.01 | (0.00, 0.01) | 0.01 | **(0.01, 0.02)** | < 0.001 |  |
|  | Model 4 | High | 0.01 | (0.00, 0.01) | 0.00 | (0.00, 0.00) | Ref (0.00) | 0.00 | (0.00, 0.01) | 0.01 | (0.00, 0.01) | < 0.001 | 0.459 |
|  |  | Moderate | 0.00 | (0.00, 0.01) | 0.00 | (0.00, 0.01) | Ref (0.00) | 0.00 | (0.00, 0.01) | 0.01 | **(0.01, 0.01)** | < 0.001 |  |
|  |  | Low | 0.01 | (0.00, 0.01) | 0.00 | (0.00, 0.01) | Ref (0.00) | 0.01 | (0.00, 0.01) | 0.01 | **(0.01, 0.02)** | < 0.001 |  |
| Education |  |  |  |  |  |  |  |  |  |  |  |  |  |
|  | Model 1 | Above high school | 0.03 | **(0.03, 0.04)** | 0.01 | **(0.01, 0.02)** | Ref (0.00) | 0.00 | (0.00, 0.01) | 0.03 | **(0.02, 0.03)** | < 0.001 | 0.033 |
|  |  | High school and below | 0.03 | **(0.02, 0.03)** | 0.01 | **(0.01, 0.01)** | Ref (0.00) | 0.01 | (0.00, 0.01) | 0.03 | **(0.02, 0.03)** | < 0.001 |  |
|  | Model 2 | Above high school | 0.01 | **(0.01, 0.02)** | 0.00 | (0.00, 0.01) | Ref (0.00) | 0.00 | (0.00, 0.01) | 0.01 | **(0.01, 0.02)** | < 0.001 | 0.639 |
|  |  | High school and below | 0.01 | **(0.01, 0.01)** | 0.00 | (0.00, 0.01) | Ref (0.00) | 0.01 | (0.00, 0.01) | 0.02 | **(0.01, 0.02)** | < 0.001 |  |
|  | Model 3 | Above high school | 0.01 | (0.00, 0.01) | 0.00 | (0.00, 0.00) | Ref (0.00) | 0.00 | (0.00, 0.01) | 0.01 | **(0.01, 0.01)** | < 0.001 | 0.535 |
|  |  | High school and below | 0.00 | (0.00, 0.01) | 0.00 | (0.00, 0.00) | Ref (0.00) | 0.00 | (0.00, 0.01) | 0.01 | **(0.01, 0.01)** | < 0.001 |  |
|  | Model 4 | Above high school | 0.01 | (0.00, 0.01) | 0.00 | (0.00, 0.00) | Ref (0.00) | 0.00 | (0.00, 0.01) | 0.01 | **(0.01, 0.01)** | < 0.001 | 0.562 |
|  |  | High school and below | 0.00 | (0.00, 0.01) | 0.00 | (0.00, 0.00) | Ref (0.00) | 0.00 | (0.00, 0.01) | 0.01 | **(0.01, 0.01)** | < 0.001 |  |
| Townsend deprivation index |  |  |  |  |  |  |  |  |  |  |  |  |  |
|  | Model 1 | ≤ -3.27 | 0.02 | **(0.01, 0.03)** | 0.01 | **(0.01, 0.01)** | Ref (0.00) | 0.00 | (0.00, 0.01) | 0.02 | **(0.02, 0.03)** | < 0.001 | 0.029 |
|  |  | -3.26 - -0.99 | 0.02 | **(0.02, 0.03)** | 0.01 | **(0.01, 0.01)** | Ref (0.00) | 0.00 | (0.00, 0.01) | 0.02 | **(0.02, 0.02)** | < 0.001 |  |
|  |  | ＞ -0.98 | 0.04 | **(0.04, 0.05)** | 0.02 | **(0.02, 0.02)** | Ref (0.00) | 0.01 | **(0.01, 0.01)** | 0.04 | **(0.04, 0.04)** | < 0.001 |  |
|  | Model 2 | ≤ -3.27 | 0.01 | (0.00, 0.01) | 0.00 | (0.00, 0.01) | Ref (0.00) | 0.00 | (0.00, 0.01) | 0.01 | **(0.01, 0.02)** | < 0.001 | 0.043 |
|  |  | -3.26 - -0.99 | 0.01 | (0.00, 0.01) | 0.00 | (0.00, 0.00) | Ref (0.00) | 0.00 | (0.00, 0.01) | 0.01 | **(0.01, 0.01)** | < 0.001 |  |
|  |  | ＞ -0.98 | 0.02 | **(0.01, 0.02)** | 0.01 | (0.00, 0.01) | Ref (0.00) | 0.01 | (0.00, 0.01) | 0.02 | **(0.02, 0.02)** | < 0.001 |  |
|  | Model 3 | ≤ -3.27 | 0.00 | (-0.01, 0.01) | 0.00 | (0.00, 0.00) | Ref (0.00) | 0.00 | (0.00, 0.01) | 0.01 | **(0.01, 0.01)** | < 0.001 | 0.120 |
|  |  | -3.26 - -0.99 | 0.00 | (0.00, 0.01) | 0.00 | (0.00, 0.00) | Ref (0.00) | 0.00 | (0.00, 0.01) | 0.01 | (0.00, 0.01) | 0.003 |  |
|  |  | ＞ -0.98 | 0.01 | (0.00, 0.01) | 0.00 | (0.00, 0.01) | Ref (0.00) | 0.01 | (0.00, 0.01) | 0.01 | **(0.01, 0.02)** | < 0.001 |  |
|  | Model 4 | ≤ -3.27 | 0.00 | (0.00, 0.01) | 0.00 | (0.00, 0.00) | Ref (0.00) | 0.00 | (0.00, 0.01) | 0.01 | **(0.01, 0.01)** | < 0.001 | 0.120 |
|  |  | -3.26 - -0.99 | 0.00 | (0.00, 0.01) | 0.00 | (0.00, 0.00) | Ref (0.00) | 0.00 | (0.00, 0.01) | 0.01 | (0.00, 0.01) | 0.002 |  |
|  |  | ＞ -0.98 | 0.01 | (0.01, 0.01) | 0.00 | (0.00, 0.01) | Ref (0.00) | 0.01 | (0.00, 0.01) | 0.02 | **(0.01, 0.02)** | < 0.001 |  |

^a^The variables adjusted in each model were the factors mentioned above except the stratification variables.

^b^Data were listed as beta estimates and 95% confidence intervals, with the bold font lower than the adjusted critical level of significance using Bonferroni’s correction.

^c^Ref, reference.

^d^Tests for trends based on the variables containing the median values for each group.

**Supplementary figure 1.** Correlation matrix of chronological age and predicted age metrics (Pearson correlation).


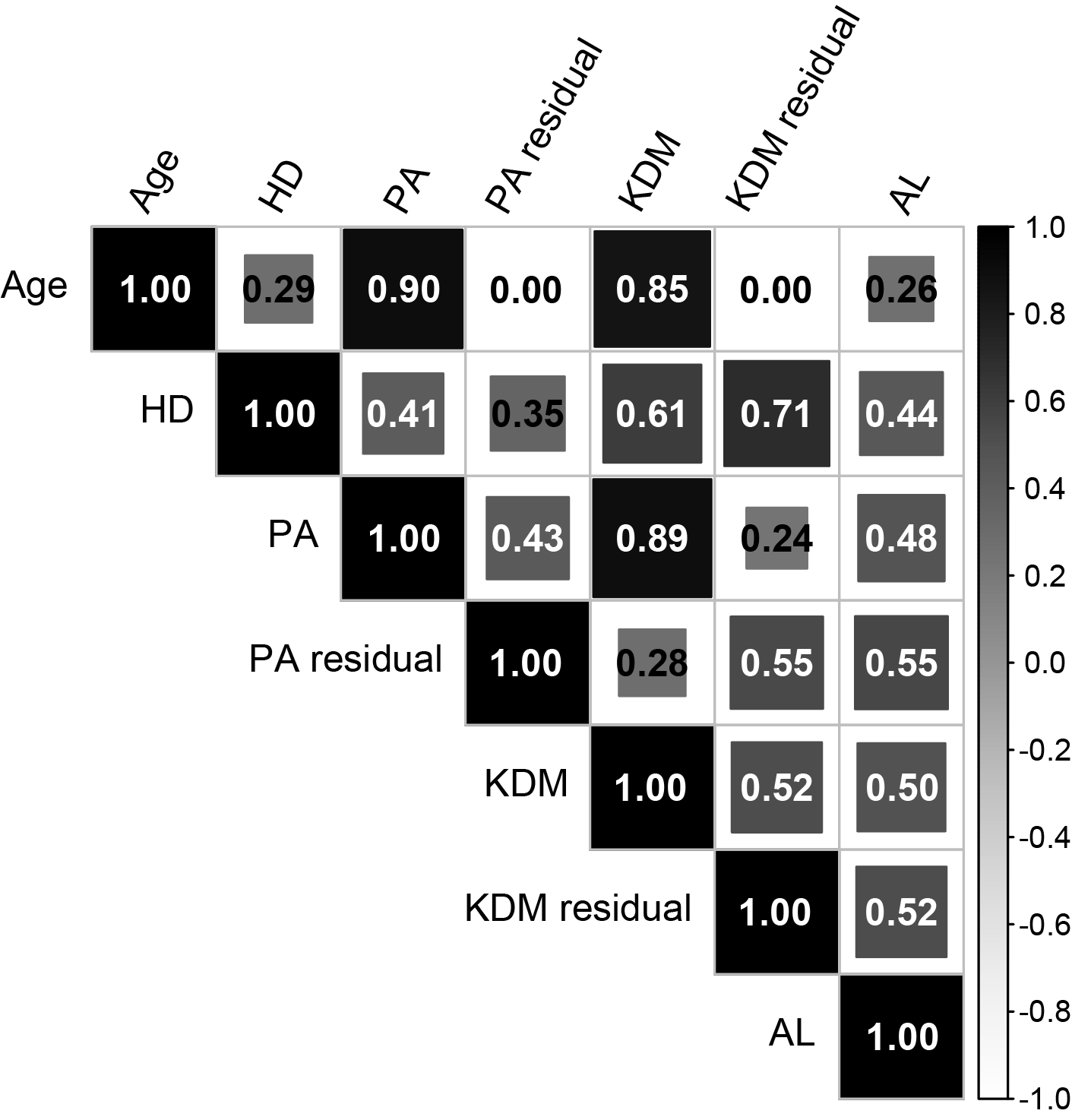


**Supplementary figure 2.** Associations between sleep duration and predicted age metrics among participants with PRSs for different predicted age metrics at baseline. Data were beta estimates and 95% CI calculated using multivariate linear regression models. Results were adjusted for age, sex, ethnicity, BMI, smoking, drinking, regular exercise, education, Townsend deprivation index, diet score, overall health rating, self-reported diabetes, hypertension, CVD, cancer, medication for cholesterol, blood pressure or diabetes, family history of diabetes, hypertension, CVD, cancer, sleep disorders, depression and shift work. Those who slept for 7 hours/day were the reference.

**
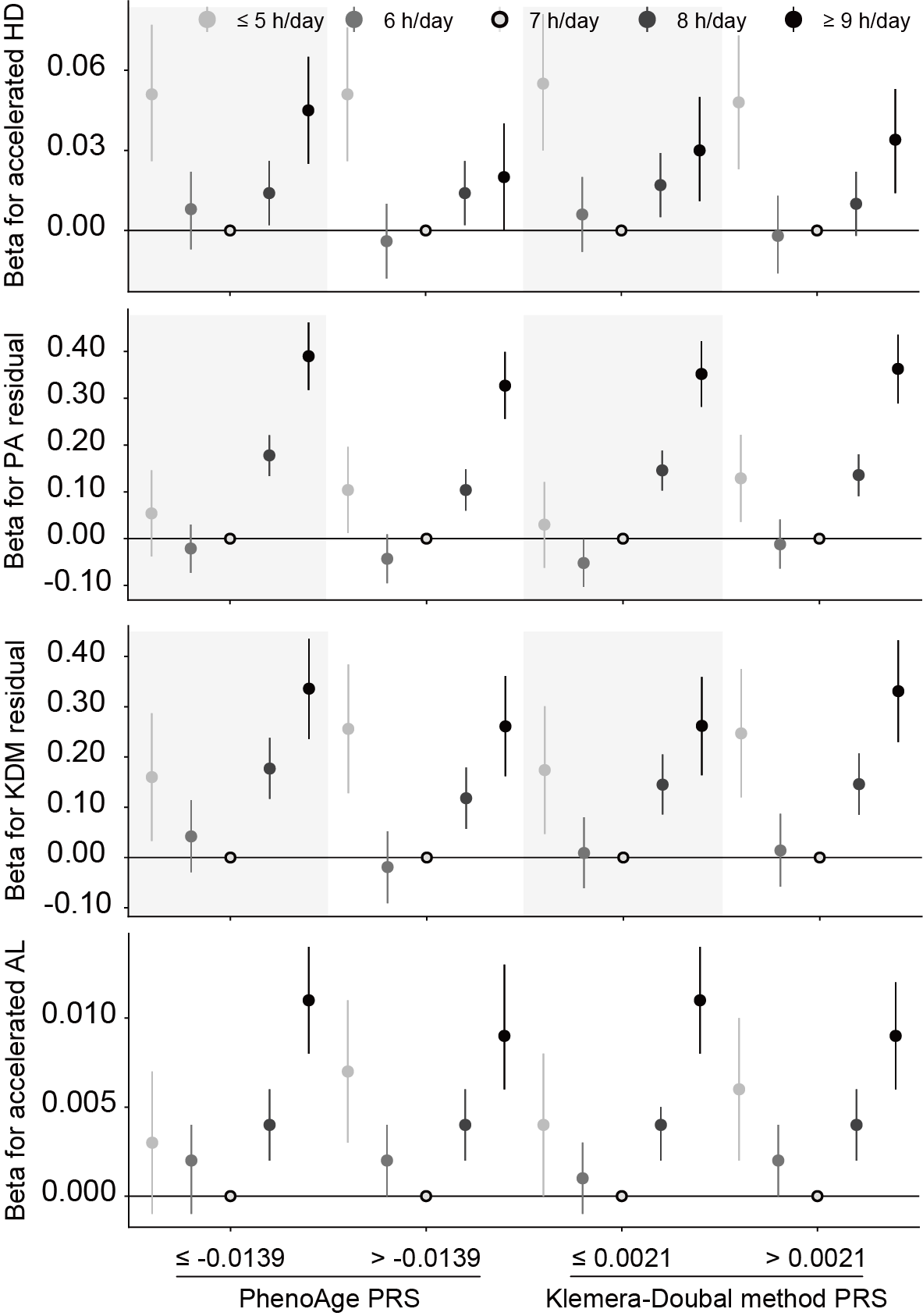
**

**Supplementary figure 3.** Smoothing curve for the association of sleep duration expressed as hours/day with CysC and GGT. Results were adjusted for age, sex, ethnicity, BMI, smoking, drinking, regular exercise, education, Townsend deprivation index, diet score, overall health rating, self-reported diabetes, hypertension, CVD, cancer, medication for cholesterol, blood pressure or diabetes, family history of diabetes, hypertension, CVD, cancer, sleep disorders, depression and shift work. P for overall was calculated using the joint Wald test for both the linear and nonlinear terms of RCS, whereas the P for nonlinear was obtained through the Wald test specifically for the non-linear term of RCS.

**
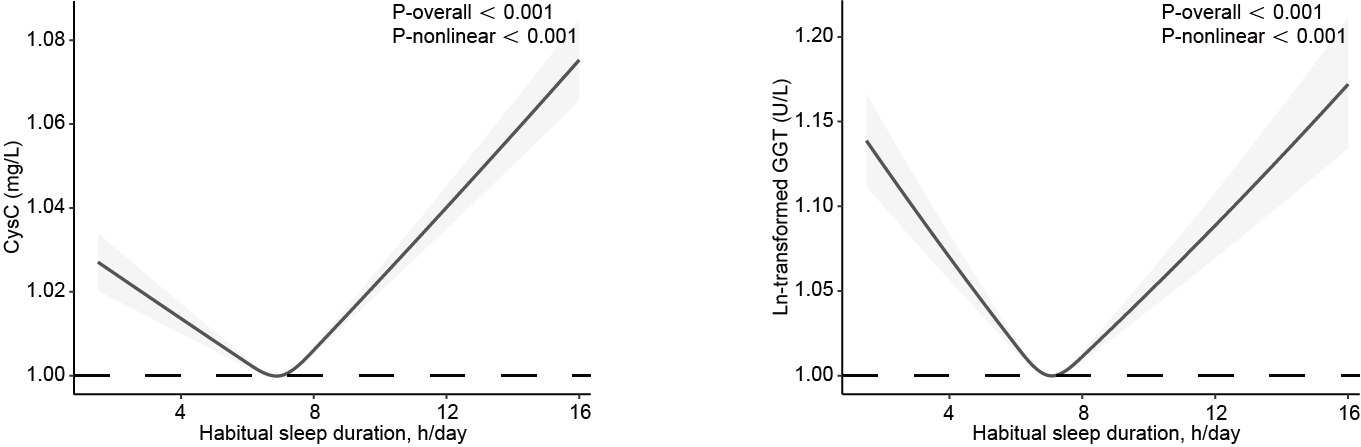
**

**Supplementary figure 4.** Effects mediated by CysC and GGT on the relationship between sleep duration and predicted age metrics. The results were listed as standardized regression coefficients after adjusting for the covariates in the full model of binomial logistic regression. Habitual sleep duration was the predictor variable (X); serum CysC and GGT were the mediators (M); accelerated HD, PA, KDM, and AL were the outcome variables (Y). Briefly, the analysis consisted of four steps: (1) determining the relation between X and Y: Model Y = β_Tot_ X; (2) determining the relation between X and M: Model M = β_indirect 1 or 2_ X; (3) determining the relation of M and Y with X: Model Y = β_indirect 3 or 4_ M + β_dir_ X; (4) calculating the proportion of the effect of the mediators on the outcomes: effect size (%) = (β_indirect 1 or 2_×β_indirect 3 or 4_/β_Tot_)×100%. (β_indirect 1 or 2_: indirect relation 1 or 2, β_indirect 3 or 4_: indirect relation 3 or 4, β_Tot_: total relation, and β_dir_: direct relation). *P < 0.05, **P < 0.01, ***P < 0.001.

**
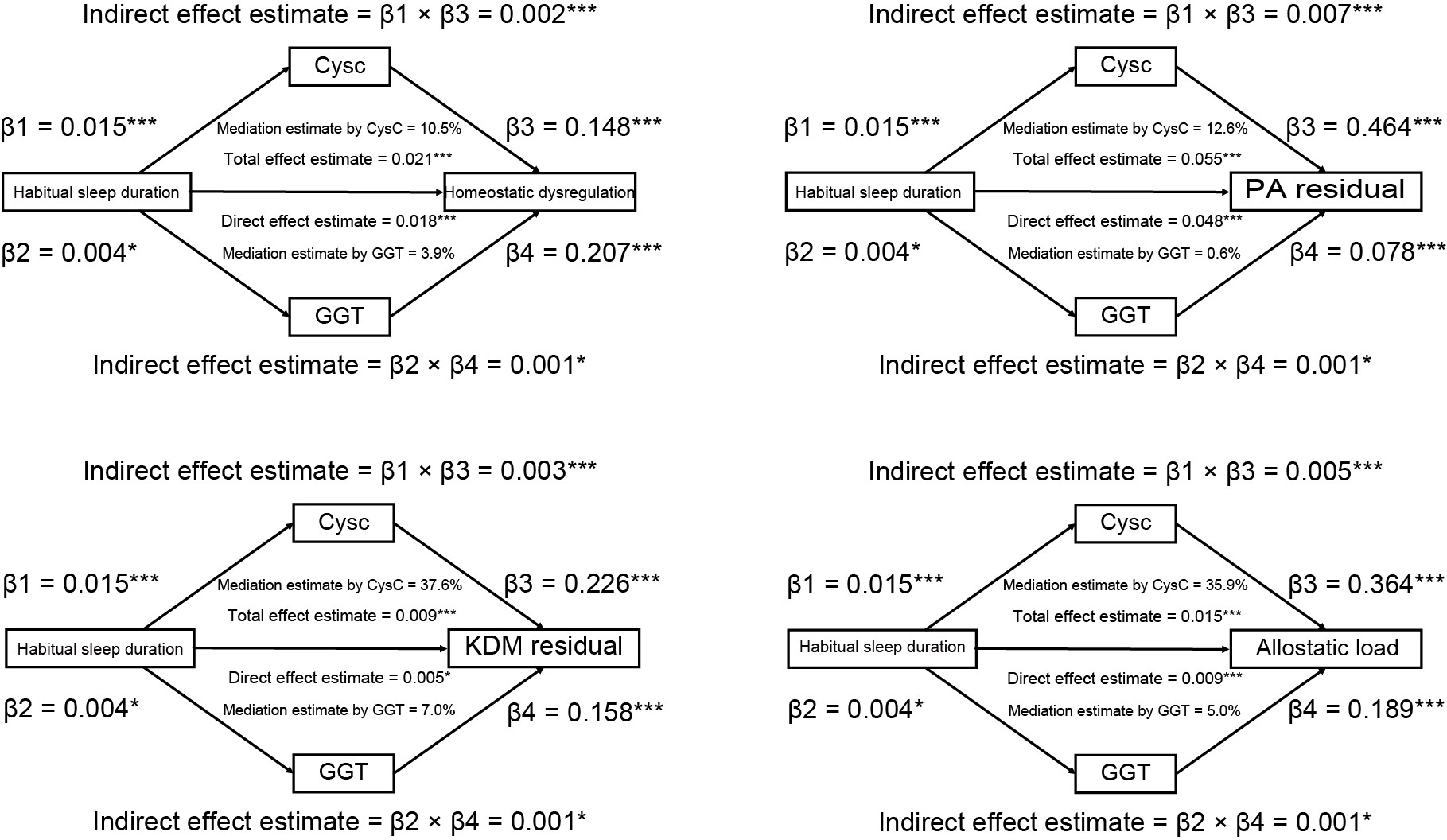
**
